# Supplementary material for: Interventions to reduce inequalities in avoidable hospital admissions: explanatory framework and systematic review protocol
Source: BMJ Open. 2020 Jul 23;10(7):e035429. doi: 10.1136/bmjopen-2019-035429 (PMC7380849; doi:10.1136/bmjopen-2019-035429)
Supplement: Supplementary data [file bmjopen-2019-035429supp002.pdf]

Supplementary file 2 – preliminary MEDLINE search strategy

Element	Search items
Hospital admissions [AND]	Hospitals/statistics & numerical data or Emergency Service, Hospital/statistics & numerical data* or Hospitalization/ec, td [Economics, Trends] or Patient Readmission/ or Patient Admission"/ec, td [Economics, Trends] or Emergency hospital admission*.ti,ab or emergency hospitali#ation.ti,ab or unplanned hospitali#ation.ti,ab or (overnight stay adj5 admission*).ti,ab or (primary care adj5 admission*).ti,ab or (ambulatory care adj5 admission*).ti,ab.or (admission* adj5 emergenc*).ti,ab.or (Emergency Treatment adj5 admission*).ti,ab.or . ((emergency care adj5 admission*) or readmission*).mp or (emergency room adj5 admission*).ti,ab. or emergency admission*.mp.or overnight stay.mp. or emergency medical admission*.mp.or (hospital* adj5 readmission rates).mp.or ((unscheduled or unplanned or un-planned or unanticipated or unexpected) adj5 (admission* or readmission* or hospitali#ation or care)).mp or (admissions adj5 hospital days).mp.or . (hospital admission* adj5 emergenc*).mp.
Equity [AND}	Combined equity filter MEDLINE – see additional file 1[1]
Intervention [AND]	exp."Health Care Quality, Access.mp. or Health Care.mp. or exp "Delivery of Health Care"/ or Chronic Disease/therapy* or State Medicine/statistics & numerical data or programme evaluation.mp or intervention$.tw. or evaluat$.tw. or policy.tw or strategy.tw. or access to care.mp.or access to services.mp. or scheme.tw or program*.tw or initaitve.tw or ambulatory care/ or aftercare/ or after-hours care/ or case management/ or “continuity of patient care”/ or patient education/ or health services accessibility/ or “deliver of health care”/ or Home Care Services, Hospital-Based/ or Managed Care Program*/ or Health Knowledge, Attitudes, Practice/ or *”Outcome Assessment (Health Care)”/ or “Drug Utili$ation Review”/ or Intermediate Care Facvilities/ or *”Self Care”/ or Community Health Services/ or “Patient Discharge”/ or Health Services Research/ or Primary Health Care/ or Physicians, Family/ or Treatment Outcome/ or Risk Assessment/ or Telemedicine/ or Quality of care.mp. or (out-of-hours or OOH).mp. or access to care.mp or access to services.mp or continuity of care.mp or medication review.mp or organisation of care.mp or outreach.mp or community matron.mp or walk in centres.mp or telemonitoring.mp or “hospital at home”.mp or “virtual wards”.mp or self management.mp or assessment units.mp or observation wards.mp or GPS in A&E.mp or ddischarge plan*.or mp primary care.mp or telephone follow-up.mp or home telecare.mp or on-line health.mp (e0health or ehealth).mp or home telemedicine.mp or general practitioner*.mp or health Promotion/ or health promotion.ti,ab. or health behaviour.ti,ab. or health behavior.ti,ab. or (policy and (social or public or urban or environmental or fiscal)).ti,ab. or urban planning.ti,ab. or city planning.ti,ab. or built environment.ti,ab. or social environment.ti,ab. or physical environment.ti,ab. or cultural environment.ti,ab. or urban environment.ti,ab. or neighbourhood.ti,ab. or community.ti,ab. or societal.ti,ab. or social interventions.ti,ab. or community interventions.ti,ab. or individual level.ti,ab. or lifestyle.ti,ab. or individual.ti,ab. or tax$.ti,ab. or subsid$.ti,ab. or price$.ti,ab. or health education.ti,ab. or social marketing.ti,ab. or (diet and (advice or counselling)).ti,ab. or (exercise and (advice or counselling)).ti,ab. or cash transfer$.ti,ab. or lifestyle counselling.ti,ab. or behavioural counselling.ti,ab. or behavioral counselling.ti,ab. or workplace.ti,ab. or campaign$.ti,ab. or (access adj1 facilities).ti,ab
Study type [AND]	(evaluat$ or effective$ or Intervention or RCT or experiment$ or randomi?ed controlled trial$ or clinical randomi?ed controlled trial$ or cluster randomi?ed controlled trial$ or double blind randomi?ed controlled trial$ or randomi?ed consent design or single blind randomi?ed controlled trial$ or randomi?ed or placebo or random$ or trial or quasi?experiment$ or pre$test or post$test or trial or time series or evaluat$ or intervention$ or "before and after" or intervention$ or commun! ity trial or non?randomi?ed or repeat$ or repeat$ measures).ti,ab. or (exp Clinical Trial/ or exp Randomized Controlled Trial/ or exp Randomization/ or exp Double-Blind Method/ or exp Single-Blind Method/ or exp Cross-Over Studies/) or clinical trial.ti,ab. or latin square.ti,ab. or random$.ti,ab. or exp Evaluation/ or clinical trial.ti,ab. or clinical trial.pt. or (before adj1 after adj1 (stud$ or trial$ or design$)).ti,ab. or random$.ti,ab. or (quasi?experimental or pseudo?experimental).ti,ab. or (nonrandomi?ed or non?randomi?ed or pseudo?randomi?sed or quasi?randomi?ed).ti,ab. or ((population level or population based or population orientated or population oriented or

	community level or community based or community orientated or community oriented) adj3 (intervention$ or prevention or policy or policies or program$ or project$)).ti,ab.
OECD countries only [NOT]	(Algeria$ or Egypt$ or Liby$ or Morocc$ or Tunisia$ or Western Sahara$ or Angola$ or Benin or Botswana$ or Burkina Faso or Burundi or Cameroon or Cape Verde or Central African Republic or Chad or Comoros or Congo or Djibouti or Eritrea or Ethiopia$ or Gabon or Gambia$ or Ghana or Guinea or Keny$ or Lesotho or Liberia or Madagasca$ or Malawi or Mali or Mauritania or Mauritius or Mayotte or Mozambiq$ or Namibia$ or Niger or Nigeria$ or Reunion or Rwand$ or Saint Helena or Senegal or Seychelles or Sierra Leone or Somalia or South Africa$ or Sudan or Swaziland or Tanzania or Togo or Ugand$ or Zambia$ or Zimbabw$ or China or Chinese or Hong Kong or Macao or Mongolia$ or Taiwan$ or Belarus or Moldov$ or Russia$ or Ukraine or Afghanistan or Armenia$ or Azerbaijan or Bahrain or Cyprus or Cypriot or Georgia$ or Iran$ or Iraq$ or Israel$ or Jordan$ or Kazakhstan or Kuwait or Kyrgyzstan or Leban$ or Oman or Pakistan$ or Palestin$ or Qatar or Saudi Arabia or Syria$ or Tajikistan or Turkmenistan or United Arab Emirates or Uzbekistan or Yemen or Bangladesh$ or Bhutan or British Indian Ocean Territory or Brunei Darussalam or Cambodia$ or India$ or Indonesia$ or Lao or People's Democratic Republic or Malaysia$ or Maldives or Myanmar or Nepal or Philippin$ or Singapore or Sri Lanka or Thai$ or Timor Leste or Vietnam or Albania$ or Andorra or Bosnia$ or Herzegovina$ or Bulgaria$ or Croatia$ or Estonia or Faroe Islands or Greenland or Liechtenstein or Lithuani$ or Macedonia or Malta or maltese or Romania or Serbia$ or Montenegro or Slovenia or Svalbard or Argentina$ or Belize or Bolivia$ or Brazil$ or chile or Chilean or Colombia$ or Costa Rica$ or Cuba or Ecuador or El Salvador or French Guiana or Guatemala$ or Guyana or Haiti or Honduras or Jamaica$ or Nicaragua$ or Panama or Paraguay or Peru or Puerto Rico or Suriname or Uruguay or Venezuela or developing countr$ or south America$).ti,sh. [mp=title, abstract, original title, name of substance word, subject heading word, floating sub-heading word, keyword heading word, organism supplementary concept word, protocol supplementary concept word, rare disease supplementary concept word, unique identifier, synonyms]
Filter applied	Humans only
Filter applied	1999-2019

1. Prady SL, Uphoff EP, Power M, et al. Development and validation of a search filter to identify equity-focused studies: reducing the number needed to screen. *BMC Medical Research Methodology* 2018;18(1):106. doi: 10.1186/s12874-018-0567-x
