# Supplementary material for: Interventions to reduce inequalities in avoidable hospital admissions: explanatory framework and systematic review protocol
Source: BMJ Open. 2020 Jul 23;10(7):e035429. doi: 10.1136/bmjopen-2019-035429 (PMC7380849; doi:10.1136/bmjopen-2019-035429)
Supplement: Supplementary data [file bmjopen-2019-035429supp003.pdf]

Manuscript ID bmjopen-2019-035429, entitled "Reducing inequalities in avoidable hospital admissions: explanatory framework and systematic review protocol."

DATA EXTRACTION FORM

This is an example of the data extraction form that we will use in the systematic review.
This process will be done in EPPI-reviewer[a].

**STUDY DETAILS**	
Author	
Publication year	
Funding source	
Country	e.g. USA, Mexico, etc.
Further location details / study setting details	e.g. city, state, region etc.
Health care system characteristics	
Most common type health coverage[b]	☐ autonomic coverage<br>☐ compulsory coverage,<br>☐ voluntary coverage<br>☐ no coverage
OECD Healthcare System typology[c]	☐ Supply and choice oriented public type<br>☐ Performance and primary-care oriented public type<br>☐ Regulation-oriented public type<br>☐ Low supply and low performance mixed type<br>☐ Supply and performance oriented private type
Study period	e.g. 3 years
Study type<br>(tick the appropriate option)	☐ Randomised controlled trials<br>☐ Non-randomised or uncontrolled trials<br>☐ Prospective and retrospective cohort studies (with and/or without control groups)<br>☐ Prospective repeat cross-sectional (panel) studies (with and/or with control groups)<br>☐ Ecological studies<br>☐ Economic evaluation<br>☐ Other (Please specify): ______________
Description of study methodology	
Confounders<br>*Other variables controlled for/consider in the analysis*	e.g. sex, ethnicity, age etc.
Blinding<br>*Details of this was done*	☐ No<br>☐ Yes (details): ______________________________<br>☐ N/A
**POPULATION**	
Age range	e.g. adults aged 18 and over
number of included participants/areas	

Clustering	e.g. individuals in areas etc.
Gender	☐ Men<br>☐ Women<br>☐ Other: ______
General study population characteristics (eligibility criteria for participation)	
Targeted or universal intervention?	☐ Targeted intervention<br>☐ Universal intervention
If targeted, explanation of target group	e.g. homeless groups, those in receipt of Medicaid, etc.
If universal, description of subdivision of outcome by SES.<br>*What measure/marker of SES used and what subdivision made.*	e.g. most deprived quintile/decile of population vs rest
Comparison group	e.g. another city, same group before and after, or no comparison etc
**INTERNVETION**	
Intervention Approach*<br>*Please see definitions of each intervention below*	☐ Population health and policy intervention<br>☐ Community based intervention<br>☐ Health and social care service<br>☐ Integrative intervention
Description of intervention *(programme, policy, strategy, initiative, scheme or activity)*	e.g. fluoridation of drinking water, out of hospital support package, etc.
Details of provider of the intervention	e.g. national health provider, local government, private entity, etc.
Were there variations in the intervention between target groups?	☐ No<br>☐ Yes
Who provided the intervention?	e.g. nurse, doctor, social worker, etc.
How was the intervention delivered?	e.g. telephone, face to face, etc.
Intervention date/period	
Other variables e.g. time	
**OUTCOME**	

Outcome reported in study	☐ Hospital admission/hospitalisation<br>☐ Hospital readmission<br>☐ Emergency hospital admission<br>☐ Avoidable hospital admission<br>☐ Unspecified<br>Definition____
Unit of analysis	e.g. Individual patient, lower super output area/neighbourhood, local authority, district etc.
Global or condition specific?	☐ Global<br>☐ Specific condition: ____________________
Detailed description of outcome variable	e.g. admission for complication of diabetes in patents with existing diagnosis of diabetes.
Justification for avoidable classification	e.g. One of ICD10 codes included in list of ambulatory care sensitive conditions
**Outcome Measure:**	
For the targeted interventions…	
No. of admission in intervention group	
Rate of intervention group admissions in Intervention group	
No. of admissions in control group if applicable	
Rate of admissions in control group if applicable	
Has sub-analysis of the outcome by SES and then by e.g. ethnicity, gender etc. been reported? And figures here.	☐ Yes (other variables)_______________________<br>☐ No<br>☐ Data______________________________
For universal interventions:	
What measure of SES is used?	e.g. IMD 2015 quintile of deprivation
No. of admission across SES groups	
Rate of admissions across SES groups	
OR across SES groups	
RR	
SMD	
Pooled proportions	
Measure of gradient	e.g. absolute gradient of inequality, gini coefficient etc
Other measures	

Has sub-analysis of SES and then breakdown by other variables been undertaken (e.g. ethnicity, gender)	☐ Yes (over variables):________<br>☐ No<br>☐ Data_____________________
Outcome measures in control group<br>*if applicable*	
Who benefits the most?	☐ Targeted intervention to those in most disadvantaged socioeconomic group only, focused on closing the health gap (selective: gap focused)<br>☐ Universal intervention, with additional focus on those in the most disadvantaged socioeconomic group to close the health gap (universal: gap focused).<br>☐ Redistributive intervention, a universally applied intervention but where the extent of benefit increases across the social gradient, such that the most socioeconomically advantaged are not expected to benefit at all from the intervention due to lack of need for it (selective: gradient focused).<br>☐ Proportionate universalism, universal intervention benefitting all, but with increasing benefits of intervention across the social gradient (universal: gradient focused).
Evidence for above statement	Interpretation of e.g. ORs etc. extracted
Comments/Additional relevant info	
**IMPLEMENTION**<br>*This information will be useful for assessing generalisability of findings/validity of pooling studies to do metanalysis. Details will be sought from the manuscripts and any associated papers through a linked citation search*	
Date of implementation	
Delivery dates	
Theoretical underpinning for intervention:	
Implementation context:	
Members of intervention team	
Experience level of the intervention team (planners and implementers)	
Consultation and/or collaboration processes (planning and delivery stages)	
Resources (for example time, money, staff and equipment)	

The TIDieR[d] or TIDieR-PHP[e] checklists will be used to extract further relevant information<br>*See checklists below*<br>.

a) EPPI-Centre, Social Science Research Unit, UCL Institute of Education, University of London, UK. Eppi-Reviewer 4 systematic review software, Available from: https://eppi.ioe.ac.uk/CMS/Default.aspx?alias=eppi.ioe.ac.uk/cms/er4& (accessed 30 Oct 2019)
b) 57. OECD, OECD Health System Characeristics survey, 2016, https://qdd.oecd.org/subject.aspx?Subject=hsc (accessed 13 Feb 2020)
c) 56. Reibling N, Ariaans M, Wendt C. Worlds of Healthcare: A Healthcare System Typology of OECD Countries. *Health Policy* 2019;123(7):611-20. doi: https://doi.org/10.1016/j.healthpol.2019.05.001
d) 58. Hoffmann TC, Glasziou PP, Boutron I, et al. Better reporting of interventions: template for intervention description and replication (TIDieR) checklist and guide. *BMJ : British Medical Journal* 2014;348:g1687. doi: 10.1136/bmj.g1687
e) 48. Campbell M, Katikireddi SV, Hoffmann T, et al. TIDieR-PHP: a reporting guideline for population health and policy interventions. *BMJ* 2018;361:k1079. doi: 10.1136/bmj.k1079

*Intervention Approaches

<u>Population health and policy intervention</u>

Legal, fiscal, structural, organisational, environmental, and policy interventions that seek to change health related behaviours or to modify the social and economic determinants of health. This includes interventions with other goals that bring about such changes as a by product.

<u>Community-based intervention</u>
Community resources and assets, for example local area coordination initiatives.

<u>Health and social care service</u>
In this context these might include interventions considered in previous reviews of avoidable admissions (for example, disease and medication management and education programmes, vaccination, structured discharge planning, comprehensive geriatric assessment, virtual wards, hospital at home initiatives) as well as primary care (provision, access, quality, continuity), benchmarking, predictive risk-modelling and case-management interventions.

<u>Integrative intervention</u>
Interventions that create greater synergy and closer working between domains 1 – 3 or within 3 (between for example, primary and secondary healthcare and social care). For example, social prescribing initiatives facilitate closer working between domains 2 and 3.

Campbell M, Katikireddi SV, Hoffmann T, et al. TIDieR-PHP: a reporting guideline for population health and policy interventions. *BMJ* 2018;361:k1079.

Item	Item description	Page in manuscript where item is reported	Other*
1 Brief name	Provide the name or a phrase that describes the intervention		
2 Why	Describe the logic, mechanisms, or rationale of the intervention, clearly linking intervention elements to the expected effects on immediate or longer term outcomes (or both)		
3 What materials	Describe any materials used in the intervention (including online appendices or URLs for further details). For example:<br>—informational materials (may include those provided to recipients of the intervention or in training of intervention providers)<br>—nature and value of any benefit provided (eg, cash, voucher, meal)<br>—any physical resources or infrastructure provided as part of the intervention		
4 What and how	Describe how the intervention was planned, established, and intended to be delivered. Depending on the type of intervention, it may be useful to consider:<br>—how sources of funding for the intervention and the service providers were obtained, how users were enrolled and the		

**Item**	**Item description**	**Page in manuscript where item is reported**	**Other***
	service delivered<br>—how any payments were made or benefits delivered, how qualifying conditions were implemented<br>—the entity being regulated, the scope of the regulation, permitted level of use; procedures for monitoring or enforcing compliance, and any sanctions for non-compliance<br>—how people were exposed to the intervention, whether it was provided to individuals or larger populations<br>—any underpinning legislation including name, date passed, and legislative body		
5 Who provided	Describe the provider of the intervention, including legal status and powers, field organisations and staff responsible for planning, implementation, monitoring and enforcement. Where relevant, describe intervention provider expertise and training (general or specific to the intervention)		
6 Where	Describe the type of location (eg, school, community centre) and the geographical scope of the intervention (eg, national, regional, city-wide). Where relevant, describe the historical, cultural, socioeconomic,		

**Item**	**Item description**	**Page in manuscript where item is reported**	**Other***
	or political background to the intervention		
7 When and how often	Describe when the intervention was implemented, how long it remained in place, and, if applicable, the number, duration, and scheduling of occasions		
8.1 Planned variation	Describe and provide the reason for any variation or tailoring that was planned or allowed for in the design of the intervention. Examples include differences between locations, geographical areas, population subgroups, or over time		
8.2 Unplanned variation	Describe and provide the reason for any unplanned variation or modifications in the intervention (eg, between different locations, geographical areas, population subgroups, or over time) that were made after the intervention commenced		
9.1 How well	Describe any strategies used or actions taken to maintain fidelity of the intervention (ie, to ensure that the intervention was delivered as intended)		
9.2 How well—delivery	Describe the fidelity of the intervention (ie, the extent to which the intervention was delivered as intended)		

- *If the information is not provided in the primary paper, give details of where this information is available (eg, protocol, other published papers (provide citation details), or a website (provide the URL))

Hoffmann TC, Glasziou PP, Boutron I, et al. Better reporting of interventions: template for intervention description and replication (TIDieR) checklist and guide. *BMJ : British Medical Journal* 2014;348:g1687. doi: 10.1136/bmj.g1687

Item No	Item	Pg no. in manuscript where reported
**Brief name**		
1	Provide the name or a phrase that describes the intervention	
**Why**		
2	Describe any rationale, theory, or goal of the elements essential to the intervention	
**What**		
3	Materials: Describe any physical or informational materials used in the intervention, including those provided to participants or used in intervention delivery or in training of intervention providers. Provide information on where the materials can be accessed (such as online appendix, URL)	
4	Procedures: Describe each of the procedures, activities, and/or processes used in the intervention, including any enabling or support activities	
**Who provided**		
5	For each category of intervention provider (such as psychologist, nursing assistant), describe their expertise, background, and any specific training given	
**How**		
6	Describe the modes of delivery (such as face to face or by some other mechanism, such as internet or telephone) of the intervention and whether it was provided individually or in a group	
**Where**		
7	Describe the type(s) of location(s) where the intervention occurred, including any necessary infrastructure or relevant features	
**When and How Much**		
8	Describe the number of times the intervention was delivered and over what period of time including the number of	

Item No	Item	Pg no. in manuscript where reported
	sessions, their schedule, and their duration, intensity, or dose	
**Tailoring**		
9	If the intervention was planned to be personalised, titrated or adapted, then describe what, why, when, and how	
**Modifications**		
10	If the intervention was modified during the course of the study, describe the changes (what, why, when, and how)	
**How well**		
11	Planned: If intervention adherence or fidelity was assessed, describe how and by whom, and if any strategies were used to maintain or improve fidelity, describe them	
12	Actual: If intervention adherence or fidelity was assessed, describe the extent to which the intervention was delivered as planned	
