# Supplementary material for: Interventions to reduce inequalities in avoidable hospital admissions: explanatory framework and systematic review protocol
Source: BMJ Open. 2020 Jul 23;10(7):e035429. doi: 10.1136/bmjopen-2019-035429 (PMC7380849; doi:10.1136/bmjopen-2019-035429)
Supplement: Author's manuscript [file bmjopen-2019-035429.draft_revisions.pdf]

BMJ Open

BMJ Open is committed to open peer review. As part of this commitment we make the peer review history of every article we publish publicly available.

When an article is published we post the peer reviewers' comments and the authors' responses online. We also post the versions of the paper that were used during peer review. These are the versions that the peer review comments apply to.

The versions of the paper that follow are the versions that were submitted during the peer review process. They are not the versions of record or the final published versions. They should not be cited or distributed as the published version of this manuscript.

BMJ Open is an open access journal and the full, final, typeset and author-corrected version of record of the manuscript is available on our site with no access controls, subscription charges or pay-per-view fees (http://bmjopen.bmj.com).

If you have any questions on BMJ Open's open peer review process please email info.bmjopen@bmj.com

BMJ Open

Reducing inequalities in avoidable hospital admissions: explanatory framework and systematic review protocol

Journal:	*BMJ Open*
Manuscript ID	bmjopen-2019-035429
Article Type:	Protocol
Date Submitted by the Author:	31-Oct-2019

Complete List of Authors:	Sowden, Sarah; Newcastle University, Population Health Sciences Institute<br>Nezafat-Maldonado, Behrouz; Newcastle University, Population Health Sciences Institute<br>Cookson, Richard; University of York, Centre for Health Economics<br>Thomson, Richard; Newcastle University, Population Health Sciences Institute<br>Lambert, Mark; Public Health England, North East Centre<br>Beyer, Fiona; Newcastle University, Population Health Sciences Institute<br>Bambra, Clare; Newcastle University, Population Health Sciences Institute

Keywords:	PREVENTIVE MEDICINE, PUBLIC HEALTH, Health policy < HEALTH SERVICES ADMINISTRATION & MANAGEMENT, Quality in health care < HEALTH SERVICES ADMINISTRATION & MANAGEMENT, Organisation of health services < HEALTH SERVICES ADMINISTRATION & MANAGEMENT, ACCIDENT & EMERGENCY MEDICINE

SCHOLARONE™
Manuscripts

For peer review only - http://bmjopen.bmj.com/site/about/guidelines.xhtml

I, the Submitting Author has the right to grant and does grant on behalf of all authors of the Work (as defined in the below author licence), an exclusive licence and/or a non-exclusive licence for contributions from authors who are: i) UK Crown employees; ii) where BMJ has agreed a CC-BY licence shall apply, and/or iii) in accordance with the terms applicable for US Federal Government officers or employees acting as part of their official duties; on a worldwide, perpetual, irrevocable, royalty-free basis to BMJ Publishing Group Ltd ("BMJ") its licensees and where the relevant Journal is co-owned by BMJ to the co-owners of the Journal, to publish the Work in this journal and any other BMJ products and to exploit all rights, as set out in our licence.

The Submitting Author accepts and understands that any supply made under these terms is made by BMJ to the Submitting Author unless you are acting as an employee on behalf of your employer or a postgraduate student of an affiliated institution which is paying any applicable article publishing charge ("APC") for Open Access articles. Where the Submitting Author wishes to make the Work available on an Open Access basis (and intends to pay the relevant APC), the terms of reuse of such Open Access shall be governed by a Creative Commons licence – details of these licences and which Creative Commons licence will apply to this Work are set out in our licence referred to above.

Other than as permitted in any relevant BMJ Author's Self Archiving Policies, I confirm this Work has not been accepted for publication elsewhere, is not being considered for publication elsewhere and does not duplicate material already published. I confirm all authors consent to publication of this Work and authorise the granting of this licence.

For peer review only

Reducing inequalities in avoidable hospital admissions: explanatory framework and systematic review protocol

Dr Sarah Sowden
https://orcid.org/0000-0001-9359-3463
sarah.sowden@ncl.ac.uk
+44 (0)1912083410

Institute of Health & Society
4th Floor, Baddiley-Clark Building
Newcastle University
Newcastle upon Tyne
NE2 4AX

Dr Behrouz Nezafat Maldonado behrouz.nezafat-maldonado@ncl.ac.uk Institute of Health and Society, Newcastle University, Newcastle upon Tyne, UK.
Professor Richard Cookson richard.cookson@york.ac.uk Centre for Health Economics, York University, York, UK.
Professor Richard Thomson richard.thomson@ncl.ac.uk Institute of Health and Society, Newcastle University, Newcastle upon Tyne, UK.
Dr Mark Lambert mark.lambert2@nhs.net Public Health England (North East Centre), Newcastle upon Tyne, UK.
Dr Fiona Beyer fiona.beyer@ncl.ac.uk Institute of Health and Society, Newcastle University, Newcastle upon Tyne, UK.
Professor Clare Bambra clare.bambra@ncl.ac.uk Institute of Health and Society, Newcastle University, Newcastle upon Tyne, UK.

Word count

3998

For peer review only - http://bmjopen.bmj.com/site/about/guidelines.xhtml

ABSTRACT

Introduction

There is pressure to contain costs due to rising numbers of unplanned hospital admissions across OECD countries. Alongside age, socioeconomic disadvantage is the strongest risk factor for avoidable emergency admission. This equity-focused systematic review is required for policy makers to understand what has been shown to work to reduce inequalities in hospital admissions, what doesn't work, and where the current gaps in the evidence-base are.

Methods and analysis

An initial framework shows how interventions are hypothesised to reduce socioeconomic inequalities in avoidable hospital admissions. Studies will be included if the differential effect of the intervention by socioeconomic status (education, income, occupation, social class, deprivation, poverty, or an area-based proxy for deprivation derived from place of residence) with respect to number/rates of hospital admission (unspecified, emergency or avoidable emergency) or readmissions has been reported or the intervention focused exclusively on socioeconomically disadvantaged populations.

Studies involving individuals of any age, undertaken in OECD countries, published since 1999 in any language will be included. Electronic searches will include MEDLINE, Embase, CINAHL, Cochrane CENTRAL and the Web of Knowledge platform. Electronic searches will be supplemented with full citation searches of included studies, website searches and retrieval of relevant unpublished information.

Study inclusion, data extraction and quality appraisal will be conducted by two reviewers. Meta-analysis, where possible, and narrative synthesis will be conducted. The main analysis will examine the effectiveness of interventions at reducing socioeconomic inequalities in hospital admissions. Interventions will be characterised by their domain of action and approach to addressing inequalities. For included studies, contextual information on where and how these interventions are organised, implemented and delivered will also be examined where possible.

Ethics and dissemination

Ethical approval was not required for this protocol. The research will be disseminated via peer-reviewed publication, conferences, and an open-access policy-orientated paper.

PROSPERO registration number: CRD42019153666

STRENGTHS AND LIMITATIONS OF THIS STUDY

- The study employs a rigorous international gold-standard methodology (PRISMA-E) to undertake an equity-focused systematic review and therefore will provide a comprehensive overview of existing literature on interventions that reduce socioeconomic inequalities in avoidable hospital admissions.
- By following PRISMA-E guidelines this study includes a framework to explain how interventions might reduce inequalities in hospital admissions which will be useful to decision makers.
- The review adopts a newly developed and validated comprehensive search strategy for identifying studies focused on equity issues and is therefore at the forefront of developments in equity-based systematic reviewing practice.
- Nevertheless, a limitation remains that by not conducting full-text hand-searching on equity related terms the review may miss studies which have not made any reference to this aspect of their analysis in either the title or abstract.
- Studies may be too heterogeneous to obtain combined effect estimates.

KEYWORDS

Healthcare Disparities, Health Equity, Socioeconomic factors, Hospitalization, Systematic review

For peer review only - http://bmjopen.bmj.com/site/about/guidelines.xhtml

INTRODUCTION

Due to pervasive socioeconomic inequalities in health[1], people in disadvantaged neighbourhoods are more in need of care. Often, however, they have worse access to, experience of, and outcomes from healthcare[2]. Potentially avoidable hospital admission for chronic conditions account for over 37 million bed days each year across the European Union (EU)[3]. Alongside age, socioeconomic disadvantage is the strongest risk factor for avoidable emergency admission[4]. To take England as an example, in 2015 263,894 excess avoidable emergency admissions were associated with socioeconomic inequality[5] and excess hospitalisations associated with socioeconomic inequality cost the NHS £4.8 billion per year[6], much of which is due to avoidable emergency admissions. Reducing pressures on hospital services therefore requires addressing the socioeconomic differences in health risks and behaviours that give rise to inequalities in avoidable hospital admissions[7]. There are national variations in rates of avoidable emergency admissions[3] as well as local variation in avoidable emergency admission inequality[8] in OECD countries. Limited research on the latter in England, suggests this does not appear to be explained by changes in the socioeconomic status of neighbourhoods over time (gentrification), or changes in certain primary care supply or quality metrics[9].

Policy context

There is growing pressure to contain costs and prevent disruptions in elective hospital care due to a rising number of unplanned hospital admissions across OECD countries[10]. Getting health and social care systems working better together to reduce avoidable admissions[7 11 12] is a key priority[13 14]. Addressing inequalities and the better integration of services focused on place based working has a high profile in the UK[15]and internationally[16]. WHO Europe Health 2020 framework aims to improve health for all and reduce health inequalities through investment in health, tacking major disease burdens, strengthening people-centred health systems and public health capacity and creating supportive environments and resilient communities[17].

Some countries have explicit statutory duties to address health inequalities. In Spain the Royal Decree Law 7/2018[18], recognises access to the National Health System as a fundamental right of every person in Spain. In addition, Spanish health regulations such as The General Health Act (1986)[19] and the National Health System Cohesion and Quality Act (2003)[20] aim to overcome health inequalities and guarantee equality of access to public health care services. Similarly, the Health and Social Care Act 2012[21] in England mandates each local area in England to take steps to address inequalities in healthcare outcomes and tackling health inequalities is seen as critical to ensuring the long term sustainability of the UK national health service[22].

There are promising interventions to reduce avoidable hospital admissions in areas including education, self-management, rehabilitation and telemedicine[12]. However, existing systematic reviews have examined *only* the effects of interventions on reducing levels of hospital admissions *overall*, as opposed to the effects on *inequalities* in avoidable hospital admissions. The differential effectiveness of interventions across social groups in unknown, including a lack of evidence on any interventions aimed at reducing inequalities or targeted in their delivery to disadvantaged groups[4 12].

It is critical for policy-making in this area that evidence of the effectiveness of different types of interventions at tackling inequalities is systematically reviewed. There is currently a lack of accessible policy and practice ready evidence on what works in terms of interventions to reduce inequalities in avoidable hospital admissions. Conversely, there is limited understanding of what interventions should be avoided; frequently well intentioned interventions may increase rather than decrease inequalities[23 24]. The organisation and implementation of such interventions is also important to understand. Little is known about the effectiveness of system wide approaches to reducing avoidable admissions[12] or the impact of wider contextual factors outside the direct control of local health and care provision; real world interventions are rarely implemented in isolation, and the complex interaction of interventions with the particular context in which they are embedded determines outcomes[11 25 26]. Internationally, there is a lack of research on potential explanations for the apparent variation in health-care equality performance between local areas required for those working in policy and practice to learn quality improvement lessons[27].

Against this backdrop, this systematic review will address this deficit in the knowledge base by reviewing primary studies of the effectiveness of interventions in reducing inequalities in avoidable admissions. It will consider

For peer review only - http://bmjopen.bmj.com/site/about/guidelines.xhtml

interventions (across population health and policy-level, community, service-based and integrative domains of action) which might reduce inequalities in avoidable admissions, highlight any gaps in the evidence base and, for any interventions identified, seek to establish how such interventions are organised, implemented and delivered.

Intervention framework

In line with PRISMA-E[28-30] guidelines, as the first stage of this equity-focused review we have developed a framework[28-33] for how inequalities in avoidable hospital admissions might be tackled (figure 1). The framework outlines the pathways through which interventions are hypothesised to operate to result in reducing socioeconomic inequalities in avoidable hospital admissions over time. This framework has adopted a system-level focus to capture the complexity of place-based working[34-36]. It has been developed from existing frameworks for avoidable emergency admissions[5] and for addressing health and healthcare inequalities[37-41]. The intention is for this to be further revised iteratively[33] as evidence from the systematic review emerges and in consultation with patient, public, and policy and practice partners. In this systematic review the interventions will be grouped according to this framework (with recognition that some interventions may be cross-cutting). The term intervention is used to refer to any programme, policy, strategy, initiative, scheme or activity that has been implemented where either:

a) the outcome of the action taken has been measured in terms of the numbers or rates of admission across socioeconomic groups, or
b) the intervention was focused specifically on socioeconomically disadvantaged areas or groups and where the outcome of the intervention was measured in terms of numbers or rates of hospital admissions in this group.

Tackling socioeconomic inequality in healthcare is challenging[42 43], requiring action on a broad scale[1 2 38 41 44], and health and social care systems working together with other agencies[27 37 38 45] to bring about change. There is increasing recognition amongst policy-makers and commissioners that to tackle complex issues such as avoidable emergency admissions effectively and to reduce inequalities in health and healthcare requires integrated policy, strategy and interventions across different domains of action (population health and policy-level, community-based, health and social care service-based as well as those focused on integrating activity). Based on this understanding, this framework has characterised interventions by their domain of action (1-4) and their approach to tackling inequalities (a-d). There are four broad domains of action[15] and the orientation of activity to primary, secondary and tertiary prevention[46] shifts accordingly across the domains of action (figure 1):

1. Population health and policy-level interventions. Legal, fiscal, structural, organisational, environmental, and policy interventions that seek to change health related behaviours or to modify the social and economic determinants of health[47]. This includes interventions with other goals that bring about such changes as a by product[47].
2. Community-based interventions. Community resources and assets, for example local area coordination initiatives.
3. Health and social care service-based interventions. In this context these might include interventions considered in previous reviews of avoidable admissions[12] (for example, disease and medication management and education programmes, vaccination, structured discharge planning, comprehensive geriatric assessment, virtual wards, hospital at home initiatives) as well as primary care (provision, access, quality, continuity), benchmarking, predictive risk-modelling and case-management interventions.
4. Integrative interventions – interventions that create greater synergy and closer working between domains 1 – 3 or within 3 (between for example, primary and secondary healthcare and social care). For example, social prescribing initiatives facilitate closer working between domains 2 and 3.

According to Benach et al.[39] interventions to address inequalities may adopt one of four different approaches:

a) Targeted intervention to those in lowest socioeconomic group only, focused on closing the health gap (selective: gap focused).

b) Universal intervention, with additional focus on those in the lowest socioeconomic group to close the health gap (universal: gap focused).

For peer review only - http://bmjopen.bmj.com/site/about/guidelines.xhtml

c) Redistributive intervention, a universally applied intervention but where the extent of benefit increases across the social gradient, such that the most socioeconomically advantaged are not expected to benefit at all from the intervention due to lack of need for it (selective: gradient focused).

d) Proportionate universalism, universal intervention benefitting all, but with increasing benefits of intervention across the social gradient (universal: gradient focused).

Here, we provide three hypothetical illustrative examples of the application of this framework in the context of reducing socioeconomic inequalities in avoidable hospital admissions. Firstly, there may be a social prescribing intervention (domains 2 and 4) which is facilitated through citizens advice services and serves those who have a limiting long-term condition and are in receipt of unemployment or other welfare benefits (approach c). This intervention is designed, financed and implemented in such a way that it is successful in reducing avoidable hospital admissions for these individuals. Secondly, there may be a water fluoridation intervention (domain 1). This intervention benefits the whole population in terms of reductions in hospitalisations resulting from tooth decay but with increasing benefits across the social gradient as need for the intervention is greatest in lower socioeconomic groups where tooth-health is poorest (approach d). Frequently well intentioned interventions may increase rather than decrease inequalities[23 24]. So in a final example, there may be a virtual ward intervention (domain 3) introduced for patients in a local area that fails to adopt any approach to addressing inequalities (a-d), and so whilst the intervention may lead to a reduction in avoidable emergency admissions per se, it may maintain or increase the disparity in avoidable emergency admissions between socioeconomic groups within this locality. The application of the framework to these three hypothetical example interventions would therefore be summarised as follows; social prescribing intervention (2c,4c), water fluoridation (1d), virtual hospital ward intervention (3x), where x represents no approach to addressing inequalities has been adopted for the intervention.

METHODS AND ANALYSIS

The review will be carried out following established criteria for the good conduct and reporting of equity-focused systematic reviews using PRISMA-E guidelines[28-30] and reporting here conforms to the standards of the Preferred Reporting Items for Systematic Reviews and Meta-analysis Protocols (PRISMA-P, see supplementary file 1).

Preliminary searchers were carried out in September and October 2019 and the study registered with the PROSPERO International Prospective Register of Systematic Reviews[48] (registration number CRD42019153666) on 21 October 2019. The review is planned to commence November 2019 and is anticipated to take one year to complete.

Patient and Public Involvement

An advisory panel comprising collaborators from the English public health and healthcare system, national and international research communities, social and voluntary sector, and patient and public representatives will guide the research. The early engagement of patients and members of the public through the NIHR North East Research Design Service consumer panel at the stage of requesting NIHR funding to carry out this research has been instrumental in shaping this systematic review protocol. PPI members helped to strengthen the articulation of the study rationale. We received a powerful mandate from panel members to undertake the research based on their unanimous belief about its critical importance to the healthcare system and wider society. It became apparent during our discussions that the legal commitment to reducing inequality in service provision and outcome in England was not widespread public knowledge, and panel members suggested this was given more prominence in the background rationale.

Through the collaborator advisory panel, patients and the public will contribute, alongside academics, policy and practice partners, to the iterative development of the framework (figure 1). In order to ensure meaningful participation of PPI members as the systematic review develops we will meet regularly highlighting areas for input. Outputs from this systematic review, including a workshop event will be developed in close collaboration with both PPI and policy and practice partners.

For peer review only - http://bmjopen.bmj.com/site/about/guidelines.xhtml

Systematic review questions

- What interventions (population health, community-based, service-based and integrative) reduce socioeconomic inequalities in avoidable hospital admissions?
- What interventions (population health, community-based, service-based and integrative) maintain or increase socioeconomic inequalities in avoidable hospital admissions?

Objectives

This study has two objectives:

1. To systematically review the effectiveness of interventions (population health, community-based, service-based and integrative) in reducing socioeconomic inequalities in avoidable hospital admissions.
2. For interventions identified through the systematic review, establish where possible, how these specific interventions are organised, implemented and delivered.

Table 1 – Study inclusion and exclusion criteria

	Inclusion	Exclusion
Participants (P)	All persons of any age<br>OECD countries	Non-OECD countries
Intervention (I)	Any intervention (programme, policy, strategy, initiative, scheme or activity) across the range of domains of action (population health and policy-level, community-based, service-based and integrative)	
Comparison (C)	No intervention; usual care or practice; other interventions	
Outcomes (O)	Numbers or rates of admission across socioeconomic groups.<br>For interventions focused exclusively on socioeconomically disadvantaged populations, the number or rate of admission in this population	Studies that do not include a sub-analysis by socioeconomic group or a measure of inequality such as an absolute gradient of inequality
Study type	Randomised controlled trials<br>Non-randomised or uncontrolled trials<br>Prospective and retrospective cohort studies (with and/or without control groups)<br>Prospective repeat cross-sectional studies (with and/or with control groups)<br>Ecological studies	Descriptive studies reporting solely on avoidable emergency admission prevalence (by person, place and time)<br>Qualitative studies<br>Editorial<br>Commentary<br>Expert opinion
Study period	Published in the last 20 years (1999-2019)	Literature published before 1999
Study reporting language	Any language	

Eligibility criteria

Studies will be selected according to the criteria outlined in table 1.

Interventions

The review will examine interventions across the range of domains of action (population health and policy-level, community-based, service-based and integrative) which might reduce socioeconomic inequalities in avoidable

For peer review only - http://bmjopen.bmj.com/site/about/guidelines.xhtml

hospital admissions in individuals of any age. It will also uncover those interventions which may serve to maintain or increase inequalities.

The review will utilise the intervention framework and group interventions by their domain of action and by their approach to tackling inequalities (figure 1). Where possible the interventions will be grouped according to these criteria with the acknowledgement that some interventions might be cross-cutting.

Outcomes

Studies will be included if one of the outcome measures reported is the number or rate of hospital admission (unspecified, emergency or avoidable emergency) or readmission sub-divided by socioeconomic grouping. Studies reporting a global measure of admission across all medical conditions and those reporting condition-specific[49] hospital admission rates will be included.

The differential effect of the intervention by socioeconomic status (education, income, occupation, social class, deprivation, poverty, or an area-based proxy for deprivation derived from place of residence) with respect to number/rates of hospital admissions must have been reported. Alternatively, for interventions focused exclusively on socioeconomically disadvantaged populations, the number or rate of hospital admission in the study population is reported (no sub-division required).

Study exclusion

The search will be limited to studies published in the last 20 years (since 1999) that have been carried out in an Organisation for Economic Co-operation and Development (OECD) country to ensure contemporaneity and a degree of commonality in health system maturity and socioeconomic and demographic context.

Study design

A rigorous and inclusive international literature search will be conducted for all randomised and non-randomised controlled trials, non-controlled trials, prospective and retrospective cohort studies (with and/or without control groups), prospective repeat cross-sectional studies (with and/or with control groups) and ecological studies of the effectiveness of interventions at reducing inequalities in emergency admissions to hospital.

Information sources and search strategy

Published studies in any language meeting the inclusion criteria will be sought by searching peer-reviewed literature in electronic databases including (host sites given in parentheses): MEDLINE (Ovid), Embase (Ovid), CINAHL (EBSCO), Cochrane CENTRAL (Wiley), Science Citation Index (Web of Knowledge), Social Science Citation Index (Web of Knowledge), Conference Proceedings Citation Index – Science (Web of Knowledge), Conference Proceedings Citation Index – Social Science (Web of Knowledge). The skills of a trained information scientist (FB) will be used to implement the electronic searches (see supplementary file 2, preliminary search strategy Medline). All searches will be re-run prior to final analyses and any further studies identified retrieved for inclusion.

Web of Knowledge Citation Indices will be used to undertake full citation searches of included studies. Electronic database searches will be supplemented with website searches, included but not restricted to the Kings Fund and the Nuffield Trust. We will request relevant information on unpublished and in-progress research from key experts in the field, identified through the collaborator network for this systematic review.

Study selection

All articles identified through the search will be uploaded into Eppi-reviewer 4 software[50]. Duplicates will be removed automatically and manually. Two reviewers (SS and BN) will screen titles and abstracts (if available) of the retrieved articles to assess eligibility for inclusion. The initial 10% of papers will be double screened independently (SS, BN), and then reviewed to ensure a consistent approach to applying the inclusion criteria is being adopted. After the initial selection based on title and abstract screen, full texts of potentially eligible articles will be obtained and screened independently by both reviewers to assess eligibility for final inclusion. Disagreements at any stage will be resolved through consultation with a third reviewer (FB).

For peer review only - http://bmjopen.bmj.com/site/about/guidelines.xhtml

Data extraction

Data for included studies will be extracted by two reviewers (SS, BN), using a bespoke form within Eppi-reviewer 4 software[50]. The data extraction of one reviewer will be checked by the other and visa-versa. Any discrepancies will be resolved through discussion between the reviewers and, if consensus is not reached, with the wider project team (FB, RT, RC, ML, CB). The data extraction form will be piloted using the first five eligible articles and modified if required. The data extracted from each article will include, but not restricted to, author, publication year, funding source, study design, study setting, study period, number of included participants/areas, study population characteristics, intervention domain (1-4), intervention approach (a-d), intervention description, definition and measurement of socioeconomic status, outcome measure(s), covariates used for adjustment (certain study types), reported limitations, key conclusions. If relevant information cannot be retrieved from the published articles, where possible, the manuscript authors will be contacted to request additional data.

Risk of bias assessment

The methodological quality of the included studies will be appraised independently by two reviewers (SS, BN) using the Effective Public Health Practice Project (EPHPP) tool[51], chosen because the same tool can be used across a range of quantitative study designs. Any disagreements in reviewer quality assessment judgements will be resolved through discussion between the reviewers, and if necessary with a third reviewer (FB). The quality appraisal criteria will be used for descriptive purposes, to highlight variations between studies and to contribute to the assessment of overall strength of evidence in this topic area.

Analyses and synthesis

Data will be presented narratively as well as in tables (where possible) to describe the study designs, population and findings and to address each of the research questions. The main analysis will examine the effects of interventions across the domains of action on socioeconomic inequalities in avoidable hospital admission, categorising the approach adopted to address inequalities for each intervention, using the multi-dimensional framework outlined in Figure 1. Absolute and relative differences in outcome between socioeconomic groups, at baseline and follow-up, or across place and time (depending on study design), will be considered. Data will be summarised statistically using meta-analysis of effect measures across socioeconomic sub-groups, where appropriate, and if studies are deemed sufficiently homogeneous to combine. Similarly, for studies exclusively focussed (in terms of study setting) on socioeconomically disadvantaged population groups, the feasibility of conducting meta-analysis for studies within this category will be assessed. Where there are sufficient numbers of studies, funnel plots and, where applicable, appropriate statistical tests will be used to assess publication bias and small study effects. Eppi Reviewer 4[50] will be used to assist in the analysis.

Where applicable and feasible, interventions will also be grouped according to the acute/chronic condition for which their differential impact across socioeconomic groups on hospital admission rates was considered[49]. Where data permit, we will conduct further demographic subgroup analysis of the socioeconomic patterning of effect by age, gender and ethnicity. We will report our analyses in accordance with the PRISMA-E guidelines[28-30].

Intervention organisation, implementation and delivery

For included studies, data on the organisation, implementation and delivery of these interventions will be sought from the manuscript and any associated papers through a linked citation search. The TIDieR[52] or TIDieR-PHP[47] checklist will be used to extract relevant information. Any associated process evaluations (quantitative and qualitative) exploring how and why such interventions work will be reviewed. Examples of the implementation components that will be examined include theoretical underpinning, implementation context, experience level of the intervention team (planners and implementers), consultation and/or collaboration processes (planning and delivery stages) and resources (for example time, money, staff and equipment). The systematic review evidence combined with the implementation analysis will provide insights into what works to reduce socioeconomic inequalities in avoidable emergency admissions to hospital, why and how.

For peer review only - http://bmjopen.bmj.com/site/about/guidelines.xhtml

DISCUSSION

The review will consider interventions which reduce inequalities in avoidable emergency admission to hospital. The review will also serve as a mapping exercise of the types of interventions that have been evaluated in relation to tackling inequalities in avoidable emergency admissions, thereby highlighting any gaps in the evidence base.

For included studies, further scrutiny of any published information about them, will seek to uncover how these interventions are organised, implemented and delivered. Context is increasingly recognised as important; real world interventions are rarely implemented in isolation, and the complex interaction of interventions with the particular social context in which they are embedded determines outcomes[11 25]. However, the assessment of implementation has not featured strongly in previous reviews[12]. It is essential for policy and practice partners to learn not only that an intervention works to reduce inequalities in avoidable hospital per se, but why and how it managed to do this. This will enable local decision makers to compare the contextual situation to their own to make an assessment of both feasibility and likelihood of success of adopting a similar intervention in their specific socio-political, economic, cultural landscape.

The study design inclusion criteria in the review are broad, given that whilst trials of service-based (domain 3), and even community-based (domain 2) interventions may be likely, we expect a lack of experimental studies in relation to population health and policy-level (domain 1) and many integrative interventions (domain 4) given these tend not to be easily or routinely evaluated using experimental study designs[40].

Only a minority of intervention studies published report any outcome by a social determinant of health and locating these studies has previously been hampered by the absence of validated equity search filters[53]. Reflecting this, the PRISMA-E guidance recommended that systematic reviewers avoid filtering searches on equity terms due to poor indexing which would lead to relevant studies being missed and advised hand-searching on these terms instead[29]. However, in 2018 an equity-focused search strategy was built and assessed for sensitivity against a gold-standard set of equity focused studies[53]. Our review adopts this newly developed and validated comprehensive search strategy for identifying studies focused on equity issues and is therefore at the forefront of developments in equity-based systematic reviewing practice. Nevertheless, a limitation remains that by not conducting full-text hand-searching on equity related terms the review may miss studies which have not made any reference to this aspect of their analysis in either the title or abstract.

Our extensive search strategy, combined with the inclusive study design criteria, will ensure that a sizeable literature will be located for synthesis. A 2012 series of reviews of interventions to reduce unplanned hospital admissions found 1530 controlled trials[12]. Whilst we recognise that the literature on the effects of interventions on socioeconomic inequalities in avoidable hospital admissions is likely to be smaller, we will maximise the likelihood of locating relevant studies by taking a more inclusive approach to study design and evaluate interventions targeted at socioeconomically disadvantaged groups or areas as well as studies that include comparative data on the differential effects of an intervention across socioeconomic groups. The size of the available evidence base will also be extended, because we will adopt a place-based, whole systems perspective, considering several domains of action (population health and policy-level, community-based, service-based and integrative).

ETHICS AND DISSEIMINATION

Ethical approval is not required for this protocol or for the systematic review because it does not involve primary data collection.

We anticipate the findings for this review will contribute to an improved understanding of interventions which reduce socioeconomic inequalities in avoidable hospital admissions. Once the evidence has been synthesised a workshop will be held with invited health and social care commissioners and providers, the public health practice community who hold responsibilities for reducing health and healthcare inequalities; relevant social, voluntary and charitable sector organisations; patient and public representatives as well as national and international research community representatives to discuss the results, further refine the framework for action, aid the write-up of the study findings and facilitate the translation of the findings into practice. The research will be disseminated via

For peer review only - http://bmjopen.bmj.com/site/about/guidelines.xhtml

national and international academic and practitioner cross-over conferences, and a paper submitted to a leading journal in this field. Furthermore, a policy-orientated summary paper will be published on an open access basis so that it is freely available to practitioners and the public.

AUTHOR CONTRIBUTIONS

SS, CB, RC, RT and ML conceived this systematic review and all authors were involved in the planning and design of the study. SS registered the protocol in PROSPERO. BN and SS with support from FB undertook preliminary literature searches in Medline. SS drafted the manuscript, and all authors have read, reviewed and contributed to revising and approving the final manuscript. SS obtained the research funding. SS is the guarantor.

FUNDING STATEMENT

This work was supported by the National Institute for Health Research (NIHR) and Health Education England (HEE) Integrated Clinical Academic Lecturer Fellowship (ref CA-CL-2018-04-ST2-010). The systematic review is part of **UNFAIR**: **UN**derstanding **F**actors that explain **A**voidable hospital admission **I**nequalities - **R**esearch study (http://bit.ly/UNFAIRstudy)

The funder had no role in developing the systematic review protocol. This publication presents independent research funded by the National Institute for Health Research (NIHR) and Health Education England (HEE). The views expressed are those of the author(s) and not necessarily those of the NHS, the NIHR or the Department of Health and Social Care.

COMPETING INTERESTS

None declared.

Figure 1 – Framework for addressing socio-economic (SE) inequalities in avoidable hospital admissions

Supplementary file 1 – PRISMA-P 2015 checklist

Supplementary file 2 – preliminary MEDLINE search strategy

For peer review only - http://bmjopen.bmj.com/site/about/guidelines.xhtml

REFERENCES

1. Marmot M. Fair Society, Healthy Lives: The Marmot Review. London: Marmot Review; 2010
2. Cookson R, Propper C, Asaria M, et al. Socio-Economic Inequalities in Health Care in England. *Fiscal Studies* 2016;37(3-4):371-403. doi: 10.1111/j.1475-5890.2016.12109
3. OECD/EU (2018), Health at a Glance: Europe 2018: State ofHealth in the EU Cycle, OECD Publishing, Paris. Available from: https://doi.org/10.1787/health_glance_eur-2018-en (accessed 30 Oct 2019)
4. Purdy S, Huntley A. Predicting and preventing avoidable hospital admissions: A review. *Journal of the Royal College of Physicians of Edinburgh* 2013;43(4):340-44. doi: 10.4997/JRCPE.2013.415
5. Cookson R, Asaria M, Ali S, et al. Health equity monitoring for healthcare quality assurance. *Social Science & Medicine* 2018;198:148-56. doi: https://doi.org/10.1016/j.socscimed.2018.01.004
6. Asaria M, Doran T, Cookson R. The costs of inequality: whole-population modelling study of lifetime inpatient hospital costs in the English National Health Service by level of neighbourhood deprivation. *Journal of Epidemiology and Community Health* 2016;70(10):990-96. doi: 10.1136/jech-2016-207447
7. Roland M, Abel G. Reducing emergency admissions: are we on the right track? *BMJ : British Medical Journal* 2012;345 doi: 10.1136/bmj.e6017
8. NHS England. CCG Improvement and Assessment Framework (CCG IAF) Data Extract 2016/17 & 2017/18 2018 Available from: https://www.england.nhs.uk/publication/ccg-iaf-data-extract/(accessed 30 Oct 2019)
9. Sheringham J, Asaria M, Barratt H, et al. Are some areas more equal than others? Socioeconomic inequality in potentially avoidable emergency hospital admissions within English local authority areas. *Journal of Health Services Research & Policy* 2017;22(2):83-90. doi: 10.1177/1355819616679198
10. Elias MJ, Le Grand. Health care and cost containment in the European Union: Routledge 2019.
11. Purdy S. Avoiding hopsital admissions: What does the research evidence say? London: Kings Fund; 2010.
12. Purdy S, Paranjothy, S., Huntley, A., Thomas, R., Mann, M., Huws, D., Brindle, P., Elwyn, G. Interventions to reduce unplanned hopsital admissions: a series of systematic reviews. NIHR Research for Patient Benefit no. PB-PG-1208-18013: University of Brisol, NHS Bristol, Cardiff University, 2012.
13. NHS England. Five year forward view. London: NHS England; 2014.
14. NHS England. New care models: Vanguards - developing a blueprint for the future of NHS and care services, 2016.
15. Baker A, Bently C, Carr D, Connolly AM, Heasman M, Johnson C, Laurent C, Tran A. Reducing health inequalities: system, scale and sustainability. London: Public Health England; 2017 PHE publications gateway number: 2017226, Crown copyright. Open Government Licence v3.0;2017.
16. WHO. Ljubljana Statement on Health Equity: Accelerating Progress Towards Health and Prosperous Lives for All in the WHO European Region. WHO Regional Office for Europe High-level conference, Ljubljana, 2019.
17. WHO. Health 2020: A European policy framework and strategy for the 21st century. Copenhagen: World Health Organisation, Regional Office for Europe, 2013.
18. Spain the Royal Decree Law 7/2018. Real Decreto-ley 7/2018, de 27 de julio, sobre el acceso universal al Sistema Nacional de Salud. Published in Boletín Oficial del Estado (Número: 183, 30/07/2018, Disposición nº 10752, Páginas: 76258-76264), 2018
19. The General Health Act (1986). Published in Boletín Oficial del Estado. Ley 14/1986, de 25 de abril, general de sanida, 1986.
20. National Health System Cohesion and Quality Act (2003), Published in Boletín Oficial del Estado. Ley 16/2003, de 28 de mayo, de cohesión y calidad del Sistema Nacional de Salud, 2003.
21. Great Britain. Health and Social Care Act. London: The Stationary Office; 2012.
22. NHS England. The NHS Long Term Plan. London: NHS England; 2019.
23. White MA, Jean; Heywood, Peter. How and why do interventions that increase health overall widen inequalities within populations? In: Babones SJ, ed. Social inequality and public health. Bristol: Policy Press 2009:65-82.
24. Lorenc T, Petticrew M, Welch V, et al. What types of interventions generate inequalities? Evidence from systematic reviews. *Journal of Epidemiology and Community Health* 2013;67(2):190-93. doi: 10.1136/jech-2012-201257
25. Greenhalgh T, Humphrey C, Hughes J, et al. How Do You Modernize a Health Service? A Realist Evaluation of Whole-Scale Transformation in London. *Milbank Quarterly* 2009;87(2):391-416. doi: 10.1111/j.1468-0009.2009.00562.x
26. Varcoe C, Bungay V, Browne AJ, et al. EQUIP Emergency: study protocol for an organizational intervention to promote equity in health care. *BMC Health Serv Res* 2019;19(1):687. doi: 10.1186/s12913-019-4494-2

For peer review only - http://bmjopen.bmj.com/site/about/guidelines.xhtml

27. Cookson R AM, Ali S, Ferguson B, Fleetcroft R, Goddard M, et al. . Health Equity Indicators for the English NHS: a longitudinal whole-population study at the small-area level. *Health Serv Deliv Res* 2016;4(26)
28. Tugwell P, Petticrew M, Kristjansson E, et al. Assessing equity in systematic reviews: realising the recommendations of the Commission on Social Determinants of Health. *BMJ* 2010;341 doi: 10.1136/bmj.c4739
29. Welch V, Petticrew M, Petkovic J, et al. Extending the PRISMA statement to equity-focused systematic reviews (PRISMA-E 2012): explanation and elaboration. *Journal of Clinical Epidemiology*;70:68-89. doi: 10.1016/j.jclinepi.2015.09.001
30. Welch V, Petticrew M, Tugwell P, et al. PRISMA-Equity 2012 Extension: Reporting Guidelines for Systematic Reviews with a Focus on Health Equity. *PLOS Medicine* 2012;9(10):e1001333. doi: 10.1371/journal.pmed.1001333
31. Ray P, Trisha G, Gill H, et al. Realist review - a new method of systematic review designed for complex policy interventions. *Journal of Health Services Research & Policy* 2005;10(1_suppl):21-34. doi:10.1258/1355819054308530
32. Anderson LM, Petticrew M, Rehfuess E, et al. Using logic models to capture complexity in systematic reviews. *Research Synthesis Methods* 2011;2(1):33-42. doi: 10.1002/jrsm.32
33. Kneale D, Thomas J, Harris K. Developing and Optimising the Use of Logic Models in Systematic Reviews: Exploring Practice and Good Practice in the Use of Programme Theory in Reviews. *PLOS ONE* 2015;10(11):e0142187. doi: 10.1371/journal.pone.0142187
34. Adam T. Advancing the application of systems thinking in health. *Health Research Policy and Systems* 2014;12(1):50. doi: 10.1186/1478-4505-12-50
35. Rutter H, Savona N, Glonti K, et al. The need for a complex systems model of evidence for public health. *The Lancet*;390(10112):2602-04. doi: 10.1016/S0140-6736(17)31267-9
36. Raine R FR, Barratt H, et al. . Challenges, solutions and future directions in the evaluation of service innovations in health care and public health. *Health Services and Delivery Research* 2016;4(16) doi: 10.3310/hsdr04160
37. Kilbourne AM, Switzer G, Hyman K, et al. Advancing Health Disparities Research Within the Health Care System: A Conceptual Framework. *American Journal of Public Health* 2006;96(12):2113-21. doi: 10.2105/AJPH.2005.077628
38. Baker A. BC, Carr D., Connolly AM., Heasman M., Johnson C., Laurent C., Tran A. Reducing health inequalities: system, scale and sustainability. London: Public Health England: PHE publications gateway number: 2017226, Crown copyright. Contains information licenced under the Open Government Licence v3.0, 2017.
39. Benach J, Malmusi D, Yasui Y, et al. A new typology of policies to tackle health inequalities and scenarios of impact based on Rose's population approach. *Journal of Epidemiology and Community Health* 2013;67(3):286-91. doi: 10.1136/jech-2011-200363
40. Bambra CL, Hillier FC, Moore HJ, et al. Tackling inequalities in obesity: a protocol for a systematic review of the effectiveness of public health interventions at reducing socioeconomic inequalities in obesity among adults. *Systematic Reviews* 2013;2(1):27. doi: 10.1186/2046-4053-2-27
41. Whitehead M, Dahlgren G. What can be done about inequalities in health? *The Lancet* 1991;338(8774):1059-63. doi: https://doi.org/10.1016/0140-6736(91)91911-D
42. Asaria M, Ali S, Doran T, et al. How a universal health system reduces inequalities: lessons from England. *Journal of Epidemiology and Community Health* 2016;70(7):637-43. doi: 10.1136/jech-2015-206742
43. Wenzl M, McCuskee S, Mossialos E. Commissioning for equity in the NHS: rhetoric and practice. *British Medical Bulletin* 2015;115(1):5-17. doi: 10.1093/bmb/ldv031
44. Dahlgren G, Whitehead, M. Policies and Strategies to Promote Social Equity in Health. Stockholm: Institute for Futures Studies 1991.
45. Lorna A, Mark L, Vikki E, et al. A Systematic Review of the Effectiveness of Health Service Interventions Aimed at Reducing Inequalities in Health. *Journal of Health Services Research & Policy* 1996;1(2):93-103. doi: 10.1177/135581969600100207
46. Geraint L, Sheringham J, Lopez Bernal J, et al. Mastering public health: a postgraduate guide to examinations and revalidation. Second ed. Great Britain: Boca Raton : CRC Press, Taylor & Francis Group 2015.
47. Campbell M, Katikireddi SV, Hoffmann T, et al. TIDieR-PHP: a reporting guideline for population health and policy interventions. *BMJ* 2018;361:k1079. doi: 10.1136/bmj.k1079
48. Sowden S, Nezafat Maldonado B, Beyer F, Cookson R, Thomson R, Lambert M, Bambra C. Reducing inequalities in avoidable hospital admissions: a systematic review. PROSPERO 2019 CRD42019153666 Available from: https://www.crd.york.ac.uk/prospero/display_record.php?ID=CRD42019153666 (accessed 30 Oct 2019)
49. NHS England CCG Improvement and Assessment Framework (CCG IAF) 2017/18: Technical annex (version 1.1). London: NHS England; 2017.

For peer review only - http://bmjopen.bmj.com/site/about/guidelines.xhtml

50. EPPI-Centre, Social Science Research Unit, UCL Institute of Education, University of London, UK. Eppi-Reviewer 4 systematic review software, Available from: https://eppi.ioe.ac.uk/CMS/Default.aspx?alias=eppi.ioe.ac.uk/cms/er4& (accessed 30 Oct 2019)
51. Thomas, B.H., Ciliska, D., Dobbins, M., & Micucci, S. Effective Public Health Practice Project: Quality Assessment tool for quanitative studies: McMaster University, school of nursing; Available from: https://merst.ca/ephpp/ (accessed 30 Oct 2019)
52. Hoffmann TC, Glasziou PP, Boutron I, et al. Better reporting of interventions: template for intervention description and replication (TIDieR) checklist and guide. *BMJ : British Medical Journal* 2014;348:g1687. doi: 10.1136/bmj.g1687
53. Prady SL, Uphoff EP, Power M, et al. Development and validation of a search filter to identify equity-focused studies: reducing the number needed to screen. *BMC Medical Research Methodology* 2018;18(1):106. doi: 10.1186/s12874-018-0567-x

For peer review only

For peer review only - http://bmjopen.bmj.com/site/about/guidelines.xhtml

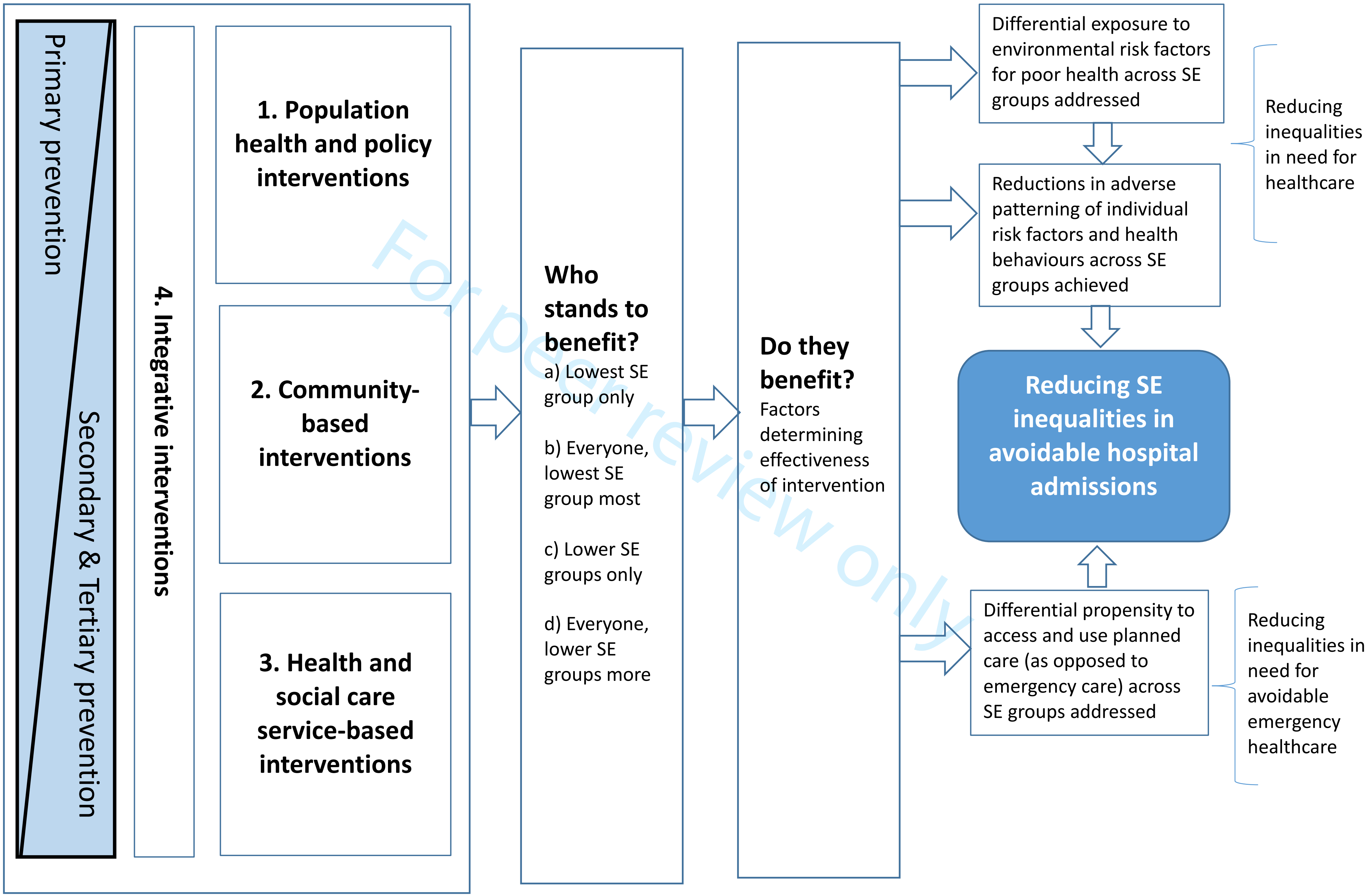
Primary prevention
Secondary & Tertiary prevention
4. Integrative interventions
1. Population health and policy interventions
2. Community-based interventions
3. Health and social care service-based interventions
Who stands to benefit?
a) Lowest SE group only
b) Everyone, lowest SE group most
c) Lower SE groups only
d) Everyone, lower SE groups more
Do they benefit?
Factors determining effectiveness of intervention
Differential exposure to environmental risk factors for poor health across SE groups addressed
Reductions in adverse patterning of individual risk factors and health behaviours across SE groups achieved
Reducing inequalities in need for healthcare
Reducing SE inequalities in avoidable hospital admissions
Differential propensity to access and use planned care (as opposed to emergency care) across SE groups addressed
Reducing inequalities in need for avoidable emergency healthcare

Reporting checklist for protocol of a systematic review.

Based on the PRISMA-P guidelines.

Moher D, Shamseer L, Clarke M, Ghersi D, Liberati A, Petticrew M, Shekelle P, Stewart LA. Preferred Reporting Items for Systematic Review and Meta-Analysis Protocols (PRISMA-P) 2015 statement. Syst Rev. 2015;4(1):1.

		Reporting Item	Page Number
**Title**			
Identification	#1a	Identify the report as a protocol of a systematic review	1
Update	#1b	If the protocol is for an update of a previous systematic review, identify as such	N/A
**Registration**			
	#2	If registered, provide the name of the registry (such as PROSPERO) and registration number	1
**Authors**			
Contact	#3a	Provide name, institutional affiliation, e-mail address of all protocol authors; provide physical mailing address of corresponding author	1
Contribution	#3b	Describe contributions of protocol authors and identify the guarantor of the review	10
**Amendments**			
	#4	If the protocol represents an amendment of a previously completed or published protocol, identify as such and list changes; otherwise, state plan for documenting important protocol amendments	N/A
**Support**			
Sources	#5a	Indicate sources of financial or other support for the review	10

For peer review only

Sponsor	#5b	Provide name for the review funder and / or sponsor	10
Role of sponsor or funder	#5c	Describe roles of funder(s), sponsor(s), and / or institution(s), if any, in developing the protocol	10
**Introduction**			
Rationale	#6	Describe the rationale for the review in the context of what is already known	3-5
Objectives	#7	Provide an explicit statement of the question(s) the review will address with reference to participants, interventions, comparators, and outcomes (PICO)	6
**Methods**			
Eligibility criteria	#8	Specify the study characteristics (such as PICO, study design, setting, time frame) and report characteristics (such as years considered, language, publication status) to be used as criteria for eligibility for the review	6-7
Information sources	#9	Describe all intended information sources (such as electronic databases, contact with study authors, trial registers or other grey literature sources) with planned dates of coverage	7
Search strategy	#10	Present draft of search strategy to be used for at least one electronic database, including planned limits, such that it could be repeated	Suppl file 2
Study records - data management	#11a	Describe the mechanism(s) that will be used to manage records and data throughout the review	8
Study records - selection process	#11b	State the process that will be used for selecting studies (such as two independent reviewers) through each phase of the review (that is, screening, eligibility and inclusion in meta-analysis)	7-8
Study records - data collection process	#11c	Describe planned method of extracting data from reports (such as piloting forms, done independently, in duplicate), any processes for obtaining and confirming data from investigators	8
Data items	#12	List and define all variables for which data will be sought (such as PICO items, funding sources), any pre-planned data	6-8

For peer review only

		assumptions and simplifications	
Outcomes and prioritization	#13	List and define all outcomes for which data will be sought, including prioritization of main and additional outcomes, with rationale	7-8
Risk of bias in individual studies	#14	Describe anticipated methods for assessing risk of bias of individual studies, including whether this will be done at the outcome or study level, or both; state how this information will be used in data synthesis	8
Data synthesis	#15a	Describe criteria under which study data will be quantitatively synthesised	8
Data synthesis	#15b	If data are appropriate for quantitative synthesis, describe planned summary measures, methods of handling data and methods of combining data from studies, including any planned exploration of consistency (such as I2, Kendall's τ)	8
Data synthesis	#15c	Describe any proposed additional analyses (such as sensitivity or subgroup analyses, meta-regression)	8
Data synthesis	#15d	If quantitative synthesis is not appropriate, describe the type of summary planned	8
Meta-bias(es)	#16	Specify any planned assessment of meta-bias(es) (such as publication bias across studies, selective reporting within studies)	8
Confidence in cumulative evidence	#17	Describe how the strength of the body of evidence will be assessed (such as GRADE)	8

Notes:

- 10: Suppl file 2 The PRISMA-P checklist is distributed under the terms of the Creative Commons Attribution License CC-BY 4.0. This checklist was completed on 31. October 2019 using https://www.goodreports.org/, a tool made by the EQUATOR Network in collaboration with Penelope.ai

For peer review only

Supplementary file 2 – preliminary MEDLINE search strategy

Element	Search items
Hospital admissions [AND]	Hospitals/statistics & numerical data or Emergency Service, Hospital/statistics & numerical data* or Hospitalization/ec, td [Economics, Trends] or Patient Readmission/ or Patient Admission"/ec, td [Economics, Trends] or Emergency hospital admission*.ti,ab or emergency hospitali#ation.ti,ab or unplanned hospitali#ation.ti,ab or (overnight stay adj5 admission*).ti,ab or (primary care adj5 admission*).ti,ab or (ambulatory care adj5 admission*).ti,ab.or (admission* adj5 emergenc*).ti,ab.or (Emergency Treatment adj5 admission*).ti,ab.or . ((emergency care adj5 admission*) or readmission*).mp or (emergency room adj5 admission*).ti,ab. or emergency admission*.mp.or overnight stay.mp. or emergency medical admission*.mp.or (hospital* adj5 readmission rates).mp.or ((unscheduled or unplanned or un-planned or unanticipated or unexpected) adj5 (admission* or readmission* or hospitali#ation or care)).mp or (admissions adj5 hospital days).mp.or . (hospital admission* adj5 emergenc*).mp.
Equity [AND}	Combined equity filter MEDLINE – see additional file 1[1]
Intervention [AND]	exp."Health Care Quality, Access.mp. or Health Care.mp. or exp "Delivery of Health Care"/ or Chronic Disease/therapy* or State Medicine/statistics & numerical data or programme evaluation.mp or intervention$.tw. or evaluat$.tw. or policy.tw or strategy.tw. or access to care.mp.or access to services.mp. or scheme.tw or program*.tw or initaitve.tw or ambulatory care/ or aftercare/ or after-hours care/ or case management/ or "continuity of patient care"/ or patient education/ or health services accessibility/ or "deliver of health care"/ or Home Care Services, Hospital-Based/ or Managed Care Program*/ or Health Knowledge, Attitudes, Practice/ or *"Outcome Assessment (Health Care)"/ or "Drug Utili$ation Review"/ or Intermediate Care Facvilities/ or *"Self Care"/ or Community Health Services/ or "Patient Discharge"/ or Health Services Research/ or Primary Health Care/ or Physicians, Family/ or Treatment Outcome/ or Risk Assessment/ or Telemedicine/ or Quality of care.mp. or (out-of-hours or OOH).mp. or access to care.mp or access to services.mp or continuity of care.mp or medication review.mp or organisation of care.mp or outreach.mp or community matron.mp or walk in centres.mp or telemonitoring.mp or "hospital at home".mp or "virtual wards".mp or self management.mp or assessment units.mp or observation wards.mp or GPS in A&E.mp or ddischarge plan*.or mp primary care.mp or telephone follow-up.mp or home telecare.mp or on-line health.mp (e0health or ehealth).mp or home telemedicine.mp or general practitioner*.mp or health Promotion/ or health promotion.ti,ab. or health behaviour.ti,ab. or health behavior.ti,ab. or (policy and (social or public or urban or environmental or fiscal)).ti,ab. or urban planning.ti,ab. or city planning.ti,ab. or built environment.ti,ab. or social environment.ti,ab. or physical environment.ti,ab. or cultural environment.ti,ab. or urban environment.ti,ab. or neighbourhood.ti,ab. or community.ti,ab. or societal.ti,ab. or social interventions.ti,ab. or community interventions.ti,ab. or individual level.ti,ab. or lifestyle.ti,ab. or individual.ti,ab. or tax$.ti,ab. or subsid$.ti,ab. or price$.ti,ab. or health education.ti,ab. or social marketing.ti,ab. or (diet and (advice or counselling)).ti,ab. or (exercise and (advice or counselling)).ti,ab. or cash transfer$.ti,ab. or lifestyle counselling.ti,ab. or behavioural counselling.ti,ab. or behavioral counselling.ti,ab. or workplace.ti,ab. or campaign$.ti,ab. or (access adj1 facilities).ti,ab
Study type [AND]	(evaluat$ or effective$ or Intervention or RCT or experiment$ or randomi?ed controlled trial$ or clinical randomi?ed controlled trial$ or cluster randomi?ed controlled trial$ or double blind randomi?ed controlled trial$ or randomi?ed consent design or single blind randomi?ed controlled trial$ or randomi?ed or placebo or random$ or trial or quasi?experiment$ or pre$test or post$test or trial or time series or evaluat$ or intervention$ or "before and after" or intervention$ or commun! ity trial or non?randomi?ed or repeat$ or repeat$ measures).ti,ab. or (exp Clinical Trial/ or exp Randomized Controlled Trial/ or exp Randomization/ or exp Double-Blind Method/ or exp Single-Blind Method/ or exp Cross-Over Studies/) or clinical trial.ti,ab. or latin square.ti,ab. or random$.ti,ab. or exp Evaluation/ or clinical trial.ti,ab. or clinical trial.pt. or (before adj1 after adj1 (stud$ or trial$ or design$)).ti,ab. or random$.ti,ab. or (quasi?experimental or pseudo?experimental).ti,ab. or (nonrandomi?ed or non?randomi?ed or pseudo?randomi?sed or quasi?randomi?ed).ti,ab. or ((population level or population based or population orientated or population oriented or

	community level or community based or community orientated or community oriented) adj3 (intervention$ or prevention or policy or policies or program$ or project$)).ti,ab.
OECD countries only [NOT]	(Algeria$ or Egypt$ or Liby$ or Morocc$ or Tunisia$ or Western Sahara$ or Angola$ or Benin or Botswana$ or Burkina Faso or Burundi or Cameroon or Cape Verde or Central African Republic or Chad or Comoros or Congo or Djibouti or Eritrea or Ethiopia$ or Gabon or Gambia$ or Ghana or Guinea or Keny$ or Lesotho or Liberia or Madagasca$ or Malawi or Mali or Mauritania or Mauritius or Mayotte or Mozambiq$ or Namibia$ or Niger or Nigeria$ or Reunion or Rwand$ or Saint Helena or Senegal or Seychelles or Sierra Leone or Somalia or South Africa$ or Sudan or Swaziland or Tanzania or Togo or Ugand$ or Zambia$ or Zimbabw$ or China or Chinese or Hong Kong or Macao or Mongolia$ or Taiwan$ or Belarus or Moldov$ or Russia$ or Ukraine or Afghanistan or Armenia$ or Azerbaijan or Bahrain or Cyprus or Cypriot or Georgia$ or Iran$ or Iraq$ or Israel$ or Jordan$ or Kazakhstan or Kuwait or Kyrgyzstan or Leban$ or Oman or Pakistan$ or Palestin$ or Qatar or Saudi Arabia or Syria$ or Tajikistan or Turkmenistan or United Arab Emirates or Uzbekistan or Yemen or Bangladesh$ or Bhutan or British Indian Ocean Territory or Brunei Darussalam or Cambodia$ or India$ or Indonesia$ or Lao or People's Democratic Republic or Malaysia$ or Maldives or Myanmar or Nepal or Philippin$ or Singapore or Sri Lanka or Thai$ or Timor Leste or Vietnam or Albania$ or Andorra or Bosnia$ or Herzegovina$ or Bulgaria$ or Croatia$ or Estonia or Faroe Islands or Greenland or Liechtenstein or Lithuani$ or Macedonia or Malta or maltese or Romania or Serbia$ or Montenegro or Slovenia or Svalbard or Argentina$ or Belize or Bolivia$ or Brazil$ or chile or Chilean or Colombia$ or Costa Rica$ or Cuba or Ecuador or El Salvador or French Guiana or Guatemala$ or Guyana or Haiti or Honduras or Jamaica$ or Nicaragua$ or Panama or Paraguay or Peru or Puerto Rico or Suriname or Uruguay or Venezuela or developing countr$ or south America$).ti,sh. [mp=title, abstract, original title, name of substance word, subject heading word, floating sub-heading word, keyword heading word, organism supplementary concept word, protocol supplementary concept word, rare disease supplementary concept word, unique identifier, synonyms]
Filter applied	Humans only
Filter applied	1999-2019

1. Prady SL, Uphoff EP, Power M, et al. Development and validation of a search filter to identify equity-focused studies: reducing the number needed to screen. *BMC Medical Research Methodology* 2018;18(1):106. doi: 10.1186/s12874-018-0567-x

For peer review only

BMJ Open

Interventions to reduce inequalities in avoidable hospital admissions: explanatory framework and systematic review protocol

Journal:	*BMJ Open*
Manuscript ID	bmjopen-2019-035429.R1
Article Type:	Protocol
Date Submitted by the Author:	28-Feb-2020
Complete List of Authors:	Sowden, Sarah; Newcastle University, Population Health Sciences Institute<br>Nezafat-Maldonado, Behrouz; Newcastle University, Population Health Sciences Institute<br>Cookson, Richard; University of York, Centre for Health Economics<br>Thomson, Richard; Newcastle University, Population Health Sciences Institute<br>Lambert, Mark; Public Health England, North East Centre<br>Beyer, Fiona; Newcastle University, Population Health Sciences Institute<br>Bambra, Clare; Newcastle University, Population Health Sciences Institute
**Primary Subject Heading**:	Public health
Secondary Subject Heading:	Health policy, Health services research
Keywords:	PREVENTIVE MEDICINE, PUBLIC HEALTH, Health policy < HEALTH SERVICES ADMINISTRATION & MANAGEMENT, Quality in health care < HEALTH SERVICES ADMINISTRATION & MANAGEMENT, Organisation of health services < HEALTH SERVICES ADMINISTRATION & MANAGEMENT, ACCIDENT & EMERGENCY MEDICINE

SCHOLARONE™
Manuscripts

I, the Submitting Author has the right to grant and does grant on behalf of all authors of the Work (as defined in the below author licence), an exclusive licence and/or a non-exclusive licence for contributions from authors who are: i) UK Crown employees; ii) where BMJ has agreed a CC-BY licence shall apply, and/or iii) in accordance with the terms applicable for US Federal Government officers or employees acting as part of their official duties; on a worldwide, perpetual, irrevocable, royalty-free basis to BMJ Publishing Group Ltd ("BMJ") its licensees and where the relevant Journal is co-owned by BMJ to the co-owners of the Journal, to publish the Work in this journal and any other BMJ products and to exploit all rights, as set out in our licence.

The Submitting Author accepts and understands that any supply made under these terms is made by BMJ to the Submitting Author unless you are acting as an employee on behalf of your employer or a postgraduate student of an affiliated institution which is paying any applicable article publishing charge ("APC") for Open Access articles. Where the Submitting Author wishes to make the Work available on an Open Access basis (and intends to pay the relevant APC), the terms of reuse of such Open Access shall be governed by a Creative Commons licence – details of these licences and which Creative Commons licence will apply to this Work are set out in our licence referred to above.

Other than as permitted in any relevant BMJ Author's Self Archiving Policies, I confirm this Work has not been accepted for publication elsewhere, is not being considered for publication elsewhere and does not duplicate material already published. I confirm all authors consent to publication of this Work and authorise the granting of this licence.

For peer review only

Interventions to reduce inequalities in avoidable hospital admissions: explanatory framework and systematic review protocol

Dr Sarah Sowden
https://orcid.org/0000-0001-9359-3463
sarah.sowden@newcastle.ac.uk
+44 (0)1912083410

Population Health Sciences Institute
4th Floor, Baddiley-Clark Building
Newcastle University
Newcastle upon Tyne
NE2 4AX

Dr Behrouz Nezafat Maldonado behrouz.nezafat-maldonado@ncl.ac.uk Population Health Sciences Institute, Newcastle University, Newcastle upon Tyne, UK.
Professor Richard Cookson richard.cookson@york.ac.uk Centre for Health Economics, York University, York, UK.
Professor Richard Thomson richard.thomson@ncl.ac.uk Population Health Sciences Institute, Newcastle University, Newcastle upon Tyne, UK.
Dr Mark Lambert mark.lambert2@nhs.net Public Health England (North East Centre), Newcastle upon Tyne, UK.
Dr Fiona Beyer fiona.beyer@ncl.ac.uk Population Health Sciences Institute, Newcastle University, Newcastle upon Tyne, UK.
Professor Clare Bambra clare.bambra@ncl.ac.uk Population Health Sciences Institute, Newcastle University, Newcastle upon Tyne, UK.

For peer review only - http://bmjopen.bmj.com/site/about/guidelines.xhtml

ABSTRACT

Introduction

There is pressure to contain costs due to rising numbers of hospital admissions across OECD countries. Alongside age, socioeconomic disadvantage is the strongest risk factor for avoidable hospital admission. This equity-focused systematic review is required for policy makers to understand what has been shown to work to reduce inequalities in hospital admissions, what doesn't work, and where the current gaps in the evidence-base are.

Methods and analysis

An initial framework shows how interventions are hypothesised to reduce socioeconomic inequalities in avoidable hospital admissions. Studies will be included if the differential effect of the intervention by socioeconomic status (education, income, occupation, social class, deprivation, poverty, or an area-based proxy for deprivation derived from place of residence) with respect to hospital admission (overall or condition-specific for those classified as ambulatory care sensitive) or readmissions has been reported or the intervention focused exclusively on socioeconomically disadvantaged populations.

Studies involving individuals of any age, undertaken in OECD countries, published from 2000 to 28 February 2020 in any language will be included. Electronic searches will include MEDLINE, Embase, CINAHL, Cochrane CENTRAL and the Web of Knowledge platform. Electronic searches will be supplemented with full citation searches of included studies, website searches and retrieval of relevant unpublished information.

Study inclusion, data extraction and quality appraisal will be conducted by two reviewers. Meta-analysis, where possible, and narrative synthesis will be conducted. The main analysis will examine the effectiveness of interventions at reducing socioeconomic inequalities in hospital admissions. Interventions will be characterised by their domain of action and approach to addressing inequalities. For included studies, contextual information on where, for whom and how these interventions are organised, implemented and delivered will be examined where possible.

Ethics and dissemination

Ethical approval was not required for this protocol. The research will be disseminated via peer-reviewed publication, conferences, and an open-access policy-orientated paper.

PROSPERO registration number: CRD42019153666

STRENGTHS AND LIMITATIONS OF THIS STUDY

- The study employs a rigorous international gold-standard methodology (PRISMA-E) to undertake an equity-focused systematic review and therefore will provide a comprehensive overview of existing literature on interventions that reduce socioeconomic inequalities in avoidable hospital admissions.
- By following PRISMA-E guidelines this study includes a framework to explain how interventions might reduce inequalities in hospital admissions which will be useful to decision makers.

For peer review only - http://bmjopen.bmj.com/site/about/guidelines.xhtml

- The review adopts a newly developed and validated comprehensive search strategy for identifying studies focused on equity issues and is therefore at the forefront of developments in equity-based systematic reviewing practice.
- Nevertheless, a limitation remains that by not conducting full-text hand-searching on equity related terms the review may miss studies which have not made any reference to this aspect of their analysis in either the title or abstract.
- Studies may be too heterogeneous to obtain combined effect estimates.

KEYWORDS

Healthcare Disparities, Health Equity, Socioeconomic factors, Hospitalization, Systematic review

INTRODUCTION

Due to pervasive socioeconomic inequalities in health[1], people in disadvantaged neighbourhoods are more in need of care. Often, however, they have worse access to, experience of, and outcomes from healthcare[2]. Potentially avoidable hospital admission for chronic conditions account for over 37 million bed days each year across the European Union (EU)[3]. Alongside age, socioeconomic disadvantage is the strongest risk factor for avoidable hospital admission[4]. To take England as an example, in 2015 263,894 excess avoidable emergency admissions were associated with socioeconomic inequality[5] and excess hospitalisations associated with socioeconomic inequality cost the NHS £4.8 billion per year[6]. Reducing pressures on hospital services therefore requires addressing the socioeconomic differences in health risks and behaviours that give rise to inequalities in avoidable hospital admissions[7]. Avoidable hospital admissions occur in all OECD countries[3]. Social inequality is a likely contributor to such admissions in all countries regardless of the health system financing scheme or population characteristics. Different ethnic groups experience different avoidable admission rates, and the interrelationship between ethnicity and socioeconomic status is complex and context specific[8]. There are national variations in rates of avoidable admissions[3] as well as local variation in avoidable admission socioeconomic inequality[9] in OECD countries. Limited research on the latter in England, suggests this does not appear to be explained by changes in the socioeconomic status of neighbourhoods over time (gentrification), or changes in certain primary care supply or quality metrics[10].

Policy context

There is growing pressure to contain costs and prevent disruptions in elective hospital care due to a rising number of hospital admissions across OECD countries[11]. Getting health and social care systems working better together to reduce avoidable admissions[7 12 13] is a key priority[14 15]. Addressing inequalities and the better integration of services focused on place based working has a high profile in the UK[16]and internationally[17]. WHO Europe Health 2020 framework aims to improve health for all and reduce health inequalities through investment in health, tacking major disease burdens, strengthening people-centred health systems and public health capacity and creating supportive environments and resilient communities[18].

Some countries have explicit statutory duties to address health and healthcare inequalities. In Spain, the Royal Decree Law 7/2018[19], recognises access to the National Health System as a fundamental right of every person in Spain. In addition, Spanish health regulations such as The General Health Act (1986)[20] and the National Health System Cohesion and Quality Act (2003)[21] aim to overcome health inequalities and guarantee equality of access to public health care services. Similarly, the Health and

For peer review only - http://bmjopen.bmj.com/site/about/guidelines.xhtml

Social Care Act 2012[22] in England mandates each local area in England to take steps to address inequalities in healthcare outcomes and tackling health inequalities is seen as critical to ensuring the long term sustainability of the UK national health service[23].

There are promising interventions to reduce avoidable hospital admissions in areas including education, self-management, rehabilitation and telemedicine[13]. However, existing systematic reviews have examined *only* the effects of interventions on reducing levels of hospital admissions *overall*, as opposed to the effects on *inequalities* in avoidable hospital admissions. The differential effectiveness of interventions across social groups in unknown, including a lack of evidence on any interventions aimed at reducing inequalities or targeted in their delivery to disadvantaged groups[4 13].

It is critical for policy-making in this area that evidence of the effectiveness of different types of interventions at tackling inequalities is systematically reviewed. There is currently a lack of accessible policy and practice ready evidence on what works in terms of interventions to reduce inequalities in avoidable hospital admissions. Conversely, there is limited understanding of what interventions should be avoided; frequently well intentioned interventions may increase rather than decrease inequalities[24 25]. The organisation and implementation of such interventions is also important to understand. Little is known about the effectiveness of system wide approaches to reducing avoidable admissions[13] or the impact of wider contextual factors outside the direct control of local health and care provision; real world interventions are rarely implemented in isolation, and the complex interaction of interventions with the particular context in which they are embedded determines outcomes[12 26 27]. Internationally, there is a lack of research on potential explanations for the apparent variation in health-care equality performance between local areas required for those working in policy and practice to learn quality improvement lessons[28].

Against this backdrop, this systematic review will address this deficit in the knowledge base by reviewing primary studies of the effectiveness of interventions in reducing socioeconomic inequalities in avoidable admissions. It will consider interventions (across population health and policy-level, community, service-based and integrative domains of action) which might reduce socioeconomic inequalities in avoidable admissions, highlight any gaps in the evidence base and, for any interventions identified, seek to establish how such interventions are organised, implemented and delivered.

Intervention framework

In line with PRISMA-E[29-31] guidelines, as the first stage of this equity-focused review we have developed a framework[29-34] for how inequalities in avoidable hospital admissions might be tackled (figure 1). The framework outlines the pathways through which interventions are hypothesised to operate to result in reducing socioeconomic inequalities in avoidable hospital admissions over time. This framework has adopted a system-level focus to capture the complexity of place-based working[35-37]. It has been developed from existing frameworks for avoidable admissions[5] and for addressing health and healthcare inequalities[38-42]. The intention is for this to be further revised iteratively[34] as evidence from the systematic review emerges and in consultation with patient, public, and policy and practice partners. In this systematic review the interventions will be grouped according to this framework (with recognition that some interventions may be cross-cutting). The term intervention is used to refer to any programme, policy, strategy, initiative, scheme or activity that has been implemented where either:

a) the outcome of the action taken has been measured in terms of avoidable hospital admission across socioeconomic groups, or

For peer review only - http://bmjopen.bmj.com/site/about/guidelines.xhtml

b) the intervention was focused specifically on socioeconomically disadvantaged areas or groups and where the outcome of the intervention was measured in terms of avoidable hospital admissions in this group.

Tackling socioeconomic inequality in healthcare is challenging[43 44], requiring action on a broad scale[1 2 39 42 45], and health and social care systems working together with other agencies[28 38 39 46] to bring about change. There is increasing recognition amongst policy-makers and commissioners that to tackle complex issues such as avoidable admissions effectively and to reduce inequalities in health and healthcare requires integrated policy, strategy and interventions across different domains of action (population health and policy-level, community-based, health and social care service-based as well as those focused on integrating activity). Based on this understanding, this framework has characterised interventions by their domain of action (1-4) and their approach to tackling inequalities (a-d). There are four broad domains of action[16] and the orientation of activity to primary, secondary and tertiary prevention[47] shifts accordingly across the domains of action (figure 1):

1. Population health and policy-level interventions. Legal, fiscal, structural, organisational, environmental, and policy interventions that seek to change health related behaviours or to modify the social and economic determinants of health[48]. This includes interventions with other goals that bring about such changes as a by product[48].
2. Community-based interventions. Community resources and assets, for example local area coordination initiatives.
3. Health and social care service-based interventions. In this context these might include interventions considered in previous reviews of avoidable admissions[13] (for example, disease and medication management and education programmes, vaccination, structured discharge planning, comprehensive geriatric assessment, virtual wards, hospital at home initiatives) as well as primary care (provision, access, quality, continuity), benchmarking, predictive risk-modelling and case-management interventions.
4. Integrative interventions – interventions that create greater synergy and closer working between domains 1 – 3 or within 3 (between for example, primary and secondary healthcare and social care). For example, social prescribing initiatives facilitate closer working between domains 2 and 3.

According to Benach et al.[40] interventions to address inequalities may adopt one of four different approaches:

a) Targeted intervention to those in most disadvantaged socioeconomic group only, focused on closing the health gap (selective: gap focused).

b) Universal intervention, with additional focus on those in the most disadvantaged socioeconomic group to close the health gap (universal: gap focused).

c) Redistributive intervention, a universally applied intervention but where the extent of benefit increases across the social gradient, such that the most socioeconomically advantaged are not expected to benefit at all from the intervention due to lack of need for it (selective: gradient focused).

d) Proportionate universalism, universal intervention benefitting all, but with increasing benefits of intervention across the social gradient (universal: gradient focused).

For peer review only - http://bmjopen.bmj.com/site/about/guidelines.xhtml

Here, we provide three hypothetical illustrative examples of the application of this framework in the context of reducing socioeconomic inequalities in avoidable hospital admissions. Firstly, there may be a social prescribing intervention (domains 2 and 4) which is facilitated through citizens advice services and serves those who have a limiting long-term condition and are in receipt of unemployment or other welfare benefits (approach c). This intervention is designed, financed and implemented in such a way that it is successful in reducing avoidable hospital admissions for these individuals. Secondly, there may be a water fluoridation intervention (domain 1). This intervention benefits the whole population in terms of reductions in hospitalisations resulting from tooth decay but with increasing benefits across the social gradient as need for the intervention is greatest in lower socioeconomic groups where tooth-health is poorest (approach d). Frequently well intentioned interventions may increase rather than decrease inequalities[24 25]. So in a final example, there may be a virtual ward intervention (domain 3) introduced for patients in a local area that fails to adopt any approach to addressing inequalities (a-d), and so whilst the intervention may lead to a reduction in avoidable hospital admissions per se, it may maintain or increase the disparity in avoidable admissions between socioeconomic groups within this locality. The application of the framework to these three hypothetical example interventions would therefore be summarised as follows; social prescribing intervention (2c,4c), water fluoridation (1d), virtual hospital ward intervention (3x), where x represents no approach to addressing inequalities has been adopted for the intervention.

METHODS AND ANALYSIS

The review will be carried out following established criteria for the good conduct and reporting of equity-focused systematic reviews using PRISMA-E guidelines[29-31] and reporting here conforms to the standards of the Preferred Reporting Items for Systematic Reviews and Meta-analysis Protocols (PRISMA-P, see supplementary file 1).

Preliminary searchers were carried out in September and October 2019 and the study registered with the PROSPERO International Prospective Register of Systematic Reviews[49] (registration number CRD42019153666) on 21 October 2019. The review is planned to commence March 2020 and is anticipated to take one year to complete.

Patient and Public Involvement

An advisory panel comprising collaborators from the English public health and healthcare system, national and international research communities, social and voluntary sector, and patient and public representatives will guide the research. The early engagement of patients and members of the public through the NIHR North East Research Design Service consumer panel at the stage of requesting NIHR funding to carry out this research has been instrumental in shaping this systematic review protocol. PPI members helped to strengthen the articulation of the study rationale. We received a powerful mandate from panel members to undertake the research based on their unanimous belief about its critical importance to the healthcare system and wider society. It became apparent during our discussions that the legal commitment to reducing inequality in service provision and outcome in England was not widespread public knowledge, and panel members suggested this was given more prominence in the background rationale.

Through the collaborator advisory panel, patients and the public will contribute, alongside academics, policy and practice partners, to the iterative development of the framework (figure 1). In order to ensure meaningful participation of PPI members as the systematic review develops we will

For peer review only - http://bmjopen.bmj.com/site/about/guidelines.xhtml

meet regularly highlighting areas for input. Outputs from this systematic review, including a workshop event will be developed in close collaboration with both PPI and policy and practice partners.

Systematic review questions

- What interventions (population health, community-based, service-based and integrative) reduce socioeconomic inequalities in avoidable hospital admissions?
- What interventions (population health, community-based, service-based and integrative) maintain or increase socioeconomic inequalities in avoidable hospital admissions?

Objectives

This study has two objectives:

1. To systematically review the effectiveness of interventions (population health, community-based, service-based and integrative) in reducing socioeconomic inequalities in avoidable hospital admissions.
2. For interventions identified through the systematic review, establish where possible, how these specific interventions are organised, implemented and delivered.

Table 1 – Study inclusion and exclusion criteria

	Inclusion	Exclusion
Participants (P)	All persons of any age<br>OECD countries	Non-OECD countries
Intervention (I)	Any intervention (programme, policy, strategy, initiative, scheme or activity) across the range of domains of action (population health and policy-level, community-based, service-based and integrative)	
Comparison (C)	No intervention; usual care or practice; other interventions	
Outcomes (O)	Numbers, rates, ratios, standardise mean differences, or a gradient measure of hospitalisation overall or condition-specific hospital admission/readmission for any ambulatory care sensitive conditions across socioeconomic groupings<br>For interventions targeted exclusively at socioeconomically disadvantaged populations, hospitalisation overall or condition specific hospital admission/readmission for any ambulatory care sensitive condition.	Studies that do not include a sub-analysis by socioeconomic group or a measure of inequality such as an absolute gradient of inequality (N/A to interventions targeted exclusively at socioeconomically disadvantaged populations)

For peer review only - http://bmjopen.bmj.com/site/about/guidelines.xhtml

Study type	Randomised controlled trials<br>Non-randomised or uncontrolled trials<br>Prospective and retrospective cohort studies (with and/or without control groups)<br>Time-series data studies<br>Panel data studies (with and/or with control groups)<br>Case-control studies<br>Other ecological studies	Descriptive studies reporting solely on avoidable admission prevalence (by person, place and time)<br>Qualitative studies<br>Editorial<br>Commentary<br>Expert opinion
Study period	Published in the last 20 years (2000-2020)	Literature published before 2000
Study reporting language	Any language	

Eligibility criteria

Studies will be selected according to the criteria outlined in table 1.

Interventions

The review will examine interventions across the range of domains of action (population health and policy-level, community-based, service-based and integrative) which might reduce socioeconomic inequalities in avoidable hospital admissions in individuals of any age. It will also uncover those interventions which may serve to maintain or increase inequalities.

The review will utilise the intervention framework and group interventions by their domain of action and by their approach to tackling inequalities (figure 1). Where possible the interventions will be grouped according to these criteria with the acknowledgement that some interventions might be cross-cutting.

Outcomes

'Avoidable admissions' are those for which timely and effective ambulatory care can prevent the need for hospitalisation[50]. Different studies use different lists of ambulatory care sensitive conditions, reflecting different types of illness (for example, whether illness is considered chronic or acute), different types of care (for example, primary care, secondary care, community care), and differences in clinical coding definitions[3 50-53]. Avoidable admissions are sometimes referred to as preventable admissions. Our definition of outcome is general and permissive of all this definitional variation within and between countries. For targeted interventions focused exclusively on socioeconomically disadvantaged populations, studies reporting a global measure of hospitalisation and those reporting condition-specific[54] hospital admission measures for any ambulatory care sensitive condition will be included. For interventions not specifically targeted at socioeconomically disadvantaged populations, studies will be included only if the differential effect of the intervention by socioeconomic status (education, income, occupation, social class, deprivation, poverty, or an area-based proxy for deprivation derived from place of residence) with respect to hospitalisations is reported. For example, using a comparison of numbers, rates, ratios, standardise mean differences, or a gradient measure of hospitalisation overall or condition-specific hospital admission/readmission for any chronic ambulatory care sensitive condition across socioeconomic groupings.

For peer review only - http://bmjopen.bmj.com/site/about/guidelines.xhtml

Study exclusion

The search will be limited to studies published in the last 20 years (since 2000) that have been carried out in an Organisation for Economic Co-operation and Development (OECD) country to ensure contemporaneity and a degree of commonality in health system maturity and socioeconomic and demographic context. We recognise that OECD countries will have different characteristics including different health systems and socio-economic situations and we will interpret the interventions that we find taking into account this variation in context.

Study design

A rigorous and inclusive international literature search for experimental, quasi-experimental and causal inference based studies will be conducted. This will include randomised and non-randomised controlled trials, non-controlled trials, prospective and retrospective cohort studies (with and/or without control groups), time series data studies, panel studies (with and/or with control groups), case-control studies, and other ecological studies (for example studies utilising administrative data) of the effectiveness of interventions at reducing inequalities in avoidable admissions to hospital.

Information sources and search strategy

Published studies in any language meeting the inclusion criteria will be sought by searching peer-reviewed literature in electronic databases including (host sites given in parentheses): MEDLINE (Ovid), Embase (Ovid), CINAHL (EBSCO), Cochrane CENTRAL (Wiley), Science Citation Index (Web of Knowledge), Social Science Citation Index (Web of Knowledge), Conference Proceedings Citation Index – Science (Web of Knowledge), Conference Proceedings Citation Index – Social Science (Web of Knowledge). The skills of a trained information scientist (FB) will be used to implement the electronic searches (see supplementary file 2, preliminary search strategy Medline). All searches will be re-run prior to final analyses and any further studies identified retrieved for inclusion.

Web of Knowledge Citation Indices will be used to undertake full citation searches of included studies. Electronic database searches will be supplemented with website searches, included but not restricted to the Kings Fund and the Nuffield Trust. We will request relevant information on unpublished and in-progress research from key experts in the field, identified through the collaborator network for this systematic review.

Study selection

All articles identified through the search will be uploaded into Eppi-reviewer 4 software[55]. Duplicates will be removed automatically and manually. Two reviewers (SS and BN) will screen titles and abstracts (if available) of the retrieved articles to assess eligibility for inclusion. The initial 10% of papers will be double screened independently (SS, BN), and then reviewed to ensure a consistent approach to applying the inclusion criteria is being adopted. After the initial selection based on title and abstract screen, full texts of potentially eligible articles will be obtained and screened independently by both reviewers to assess eligibility for final inclusion. Disagreements at any stage will be resolved through consultation with a third reviewer (FB).

Data extraction

Data for included studies will be extracted by two reviewers (SS, BN), using a bespoke form within Eppi-reviewer 4 software[55]. The data extraction of one reviewer will be checked by the other and

For peer review only - http://bmjopen.bmj.com/site/about/guidelines.xhtml

visa-versa. Any discrepancies will be resolved through discussion between the reviewers and, if consensus is not reached, with the wider project team (FB, RT, RC, ML, CB). The data extraction form will be piloted using the first five eligible articles and modified if required (see supplementary file 3). The data extracted from each article will include, but not restricted to, author, publication year, funding source, study design, study setting including characteristics of the health system of the country, study period, number of included participants/areas, study population characteristics (including for example age and ethnicity), intervention domain (1-4), intervention approach (a-d), intervention description, definition and measurement of socioeconomic status, outcome measure(s), covariates used for adjustment (certain study types), reported limitations, key conclusions. It is anticipated studies will characterise socioeconomic groupings differently, and measure equity in a variety of ways so the data collection tool will be designed to ensure all ways of capturing socioeconomic status and differential outcomes across groups will be captured. If relevant information cannot be retrieved from the published articles, where possible, the manuscript authors will be contacted to request additional data.

For included studies, data on the organisation, implementation and delivery of these interventions will be sought from the manuscript and any associated papers through a linked citation search. We will use data collected from the OECD Health System Characteristic survey[56] to explore potential differences between countries and we will classify them based on the most-common health coverage that is implemented e.g. autonomic coverage, compulsory coverage, voluntary coverage or no coverage. The TIDieR[57] or TIDieR-PHP[48] checklist will be used to extract relevant contextual information. Any associated process evaluations (quantitative and qualitative) exploring how and why such interventions work will be reviewed. Examples of the implementation components that will be examined include theoretical underpinning, implementation context, experience level of the intervention team (planners and implementers), consultation and/or collaboration processes (planning and delivery stages) and resources (for example time, money, staff and equipment). The systematic review evidence combined with the implementation analysis will provide insights into what works to reduce socioeconomic inequalities in avoidable admissions to hospital, why and how.

Risk of bias assessment

The methodological quality of the included studies will be appraised independently by two reviewers (SS, BN) using the Effective Public Health Practice Project (EPHPP) tool[58], chosen because the same tool can be used across a range of quantitative study designs. Any disagreements in reviewer quality assessment judgements will be resolved through discussion between the reviewers, and if necessary with a third reviewer (FB). Using the EPHPP tool, the two reviewers will make their own scientific value judgements about whether quasi-experimental methodology studies are sufficiently well designed to draw robust causal inferences, referring controversial cases to the wider review team". In rare cases, for example, robust causal inferences can be derived from uncontrolled before-after studies (and indeed from cross sectional correlations). The quality appraisal criteria will be used for descriptive purposes, to highlight variations between studies and to contribute to the assessment of overall strength of evidence in this topic area.

Analyses and synthesis

Data will be presented narratively as well as in tables (where possible) to describe the study designs, population and findings and to address each of the research questions. The main analysis will examine the effects of interventions across the domains of action on socioeconomic inequalities in avoidable hospital admission, categorising the approach adopted to address inequalities for each intervention, using the multi-dimensional framework outlined in Figure 1. Absolute and relative

For peer review only - http://bmjopen.bmj.com/site/about/guidelines.xhtml

differences in outcome between socioeconomic groups, at baseline and follow-up, or across place and time (depending on study design), will be considered. Data will be summarised statistically using meta-analysis of effect measures across socioeconomic sub-groups, where appropriate, and if studies are deemed sufficiently homogeneous to combine. Similarly, for studies exclusively focussed (in terms of study setting) on socioeconomically disadvantaged population groups, the feasibility of conducting meta-analysis for studies within this category will be assessed. Where there are sufficient numbers of studies, funnel plots and, where applicable, appropriate statistical tests will be used to assess publication bias and small study effects. Eppi Reviewer 4[55] will be used to assist in the analysis.

Where applicable and feasible, we will synthesise results for different population sub-groups separately (for example in regards to children compared to the elderly, acute compared to chronic ambulatory care sensitive conditions) where the differential impact of interventions across socioeconomic groups on hospitalisations was considered[54]. Where data permit, we will conduct further demographic subgroup analysis of the socioeconomic patterning of effect by age, gender and ethnicity. We will report our analyses in accordance with the PRISMA-E guidelines[29-31].

DISCUSSION

The review will consider interventions which reduce socioeconomic inequalities in avoidable admission to hospital. The review will also serve as a mapping exercise of the types of interventions that have been evaluated in relation to tackling inequalities in avoidable admissions, thereby highlighting any gaps in the evidence base.

For included studies, further scrutiny of any published information about them, will seek to uncover how these interventions are organised, implemented and delivered. Context is increasingly recognised as important; real world interventions are rarely implemented in isolation, and the complex interaction of interventions with the particular social context in which they are embedded determines outcomes[12 26]. However, the assessment of context and implementation has not featured strongly in previous reviews[13]. It is essential for policy and practice partners to learn not only that an intervention works to reduce inequalities in avoidable hospital per se, but why and how it managed to do this. This will enable local decision makers to compare the contextual situation to their own to make an assessment of both feasibility and likelihood of success of adopting a similar intervention in their specific socio-political, economic, cultural landscape. The ability of the review to provide this information to assist in implementation will be constrained by the extent to which this contextual information is examined and reported on in the primary studies. For example, an intervention to address socioeconomic inequalities may work for a specific ethnic group (or other sub-population) but will not work in another, and if a sub-analysis by ethnicity of the differential effectiveness of the intervention across socioeconomic groups is not carried out, or an insufficient description of the targeted population who were exposed to the intervention is not provided, this nuance will be lost.

The study design inclusion criteria in the review are broad, given that whilst trials of service-based (domain 3), and even community-based (domain 2) interventions may be likely, we expect a lack of experimental studies in relation to population health and policy-level (domain 1) and many integrative interventions (domain 4) given these tend not to be easily or routinely evaluated using experimental study designs[41 59].

Only a minority of intervention studies published report any outcome by a social determinant of health and locating these studies has previously been hampered by the absence of validated equity

For peer review only - http://bmjopen.bmj.com/site/about/guidelines.xhtml

search filters[60]. Reflecting this, the PRISMA-E guidance recommended that systematic reviewers avoid filtering searches on equity terms due to poor indexing which would lead to relevant studies being missed and advised hand-searching on these terms instead[30]. However, in 2018 an equity-focused search strategy was built and assessed for sensitivity against a gold-standard set of equity focused studies[60]. Our review adopts this newly developed and validated comprehensive search strategy for identifying studies focused on equity issues and is therefore at the forefront of developments in equity-based systematic reviewing practice. Nevertheless, a limitation remains that by not conducting full-text hand-searching on equity related terms the review may miss studies which have not made any reference to this aspect of their analysis in either the title or abstract.

Our extensive search strategy, combined with the inclusive study design criteria, will ensure that a sizeable literature will be located for synthesis. A 2012 series of reviews of interventions to reduce unplanned hospital admissions found 1530 controlled trials[13]. Whilst we recognise that the literature on the effects of interventions on socioeconomic inequalities in avoidable hospital admissions is likely to be smaller, we will maximise the likelihood of locating relevant studies by taking a more inclusive approach to study design and evaluate interventions targeted at socioeconomically disadvantaged groups or areas as well as studies that include comparative data on the differential effects of an intervention across socioeconomic groups. The size of the available evidence base will also be extended, because we will adopt a place-based, whole systems perspective, considering several domains of action (population health and policy-level, community-based, service-based and integrative).

ETHICS AND DISSEIMINATION

Ethical approval is not required for this protocol or for the systematic review because it does not involve primary data collection.

We anticipate the findings for this review will contribute to an improved understanding of interventions which reduce socioeconomic inequalities in avoidable hospital admissions. Once the evidence has been synthesised a workshop will be held with invited health and social care commissioners and providers, the public health practice community who hold responsibilities for reducing health and healthcare inequalities; relevant social, voluntary and charitable sector organisations; patient and public representatives as well as national and international research community representatives to discuss the results, further refine the framework for action, aid the write-up of the study findings and facilitate the translation of the findings into practice. The research will be disseminated via national and international academic and practitioner cross-over conferences, and a paper submitted to a leading journal in this field. Furthermore, a policy-orientated summary paper will be published on an open access basis so that it is freely available to practitioners and the public.

AUTHOR CONTRIBUTIONS

SS, CB, RC, RT and ML conceived this systematic review and all authors were involved in the planning and design of the study. SS registered the protocol in PROSPERO. BN and SS with support from FB undertook preliminary literature searches in Medline. SS drafted and revised the manuscript, and all authors have read, reviewed and contributed to revising and approving the final manuscript. SS obtained the research funding. SS is the guarantor.

For peer review only - http://bmjopen.bmj.com/site/about/guidelines.xhtml

FUNDING STATEMENT

This work was supported by the National Institute for Health Research (NIHR) and Health Education England (HEE) Integrated Clinical Academic Lecturer Fellowship (ref CA-CL-2018-04-ST2-010) and Research Capability Funding, NHS North East Commissioning Support Service. The systematic review is part of **UNFAIR**: **UN**derstanding **F**actors that explain **A**voidable hospital admission **I**nequalities - **R**esearch study (http://bit.ly/UNFAIRstudy)

The funders had no role in developing the systematic review protocol. This publication presents independent research funded by the National Institute for Health Research (NIHR) and Health Education England (HEE). The views expressed are those of the author(s) and not necessarily those of the NHS, the NIHR or the Department of Health and Social Care.

COMPETING INTERESTS

None declared.

Figure 1 – Framework for addressing socio-economic (SE) inequalities in avoidable hospital admissions

Supplementary file 1 – PRISMA-P 2015 checklist

Supplementary file 2 – preliminary MEDLINE search strategy

Supplementary file 3 – data collection form

For peer review only

For peer review only - http://bmjopen.bmj.com/site/about/guidelines.xhtml

REFERENCES

1. Marmot M. Fair Society, Healthy Lives: The Marmot Review. London: Marmot Review; 2010

2. Cookson R, Propper C, Asaria M, et al. Socio-Economic Inequalities in Health Care in England. *Fiscal Studies* 2016;37(3-4):371-403. doi: 10.1111/j.1475-5890.2016.12109

3. OECD/EU (2018), Health at a Glance: Europe 2018: State ofHealth in the EU Cycle, OECD Publishing, Paris. Available from: https://doi.org/10.1787/health_glance_eur-2018-en (accessed 30 Oct 2019)

4. Purdy S, Huntley A. Predicting and preventing avoidable hospital admissions: A review. *Journal of the Royal College of Physicians of Edinburgh* 2013;43(4):340-44. doi: 10.4997/JRCPE.2013.415

5. Cookson R, Asaria M, Ali S, et al. Health equity monitoring for healthcare quality assurance. *Social Science & Medicine* 2018;198:148-56. doi: https://doi.org/10.1016/j.socscimed.2018.01.004

6. Asaria M, Doran T, Cookson R. The costs of inequality: whole-population modelling study of lifetime inpatient hospital costs in the English National Health Service by level of neighbourhood deprivation. *Journal of Epidemiology and Community Health* 2016;70(10):990-96. doi: 10.1136/jech-2016-207447

7. Roland M, Abel G. Reducing emergency admissions: are we on the right track? *BMJ : British Medical Journal* 2012;345 doi: 10.1136/bmj.e6017

8. Dalla Zuanna T, Spadea T, Milana M, et al. Avoidable hospitalization among migrants and ethnic minority groups: a systematic review. *European Journal of Public Health* 2017;27(5):861-68. doi: 10.1093/eurpub/ckx113

9. NHS England. CCG Improvement and Assessment Framework (CCG IAF) Data Extract 2016/17 & 2017/18 2018 Available from: https://www.england.nhs.uk/publication/ccg-iaf-data-extract/(accessed 30 Oct 2019)

10. Sheringham J, Asaria M, Barratt H, et al. Are some areas more equal than others? Socioeconomic inequality in potentially avoidable emergency hospital admissions within English local authority areas. *Journal of Health Services Research & Policy* 2017;22(2):83-90. doi: 10.1177/1355819616679198

11. Elias MJ, Le Grand. Health care and cost containment in the European Union: Routledge 2019.

12. Purdy S. Avoiding hopsital admissions: What does the research evidence say? London: Kings Fund; 2010.

13. Purdy S, Paranjothy, S., Huntley, A., Thomas, R., Mann, M., Huws, D., Brindle, P., Elwyn, G. Interventions to reduce unplanned hopsital admissions: a series of systematic reviews. NIHR Research for Patient Benefit no. PB-PG-1208-18013: University of Brisol, NHS Bristol, Cardiff University, 2012.

14. NHS England. Five year forward view. London: NHS England; 2014.

15. NHS England. New care models: Vanguards - developing a blueprint for the future of NHS and care services, 2016.

16. Baker A, Bently C, Carr D, Connolly AM, Heasman M, Johnson C, Laurent C, Tran A. Reducing health inequalities: system, scale and sustainability. London: Public Health England; 2017 PHE publications gateway number: 2017226, Crown copyright. Open Government Licence v3.0;2017.

17. WHO. Ljubljana Statement on Health Equity: Accelerating Progress Towards Health and Prosperous Lives for All in the WHO European Region. WHO Regional Office for Europe High-level conference, Ljubljana, 2019.

18. WHO. Health 2020: A European policy framework and strategy for the 21st century. Copenhagen: World Health Organisation, Regional Office for Europe, 2013.

19. Spain the Royal Decree Law 7/2018. Real Decreto-ley 7/2018, de 27 de julio, sobre el acceso universal al Sistema Nacional de Salud. Published in Boletín Oficial del Estado (Número: 183, 30/07/2018, Disposición nº 10752, Páginas: 76258-76264), 2018

For peer review only

For peer review only - http://bmjopen.bmj.com/site/about/guidelines.xhtml

20. The General Health Act (1986). Published in Boletín Oficial del Estado. Ley 14/1986, de 25 de abril, general de sanida, 1986.
21. National Health System Cohesion and Quality Act (2003), Published in Boletín Oficial del Estado. Ley 16/2003, de 28 de mayo, de cohesión y calidad del Sistema Nacional de Salud, 2003.
22. Great Britain. Health and Social Care Act. London: The Stationary Office; 2012.
23. NHS England. The NHS Long Term Plan. London: NHS England; 2019.
24. White MA, Jean; Heywood, Peter. How and why do interventions that increase health overall widen inequalities within populations? In: Babones SJ, ed. Social inequality and public health. Bristol: Policy Press 2009:65-82.
25. Lorenc T, Petticrew M, Welch V, et al. What types of interventions generate inequalities? Evidence from systematic reviews. *Journal of Epidemiology and Community Health* 2013;67(2):190-93. doi: 10.1136/jech-2012-201257
26. Greenhalgh T, Humphrey C, Hughes J, et al. How Do You Modernize a Health Service? A Realist Evaluation of Whole-Scale Transformation in London. *Milbank Quarterly* 2009;87(2):391-416. doi: 10.1111/j.1468-0009.2009.00562.x
27. Varcoe C, Bungay V, Browne AJ, et al. EQUIP Emergency: study protocol for an organizational intervention to promote equity in health care. *BMC Health Serv Res* 2019;19(1):687. doi: 10.1186/s12913-019-4494-2
28. Cookson R AM, Ali S, Ferguson B, Fleetcroft R, Goddard M, et al. . Health Equity Indicators for the English NHS: a longitudinal whole-population study at the small-area level. *Health Serv Deliv Res* 2016;4(26)
29. Tugwell P, Petticrew M, Kristjansson E, et al. Assessing equity in systematic reviews: realising the recommendations of the Commission on Social Determinants of Health. *BMJ* 2010;341 doi: 10.1136/bmj.c4739
30. Welch V, Petticrew M, Petkovic J, et al. Extending the PRISMA statement to equity-focused systematic reviews (PRISMA-E 2012): explanation and elaboration. *Journal of Clinical Epidemiology*;70:68-89. doi: 10.1016/j.jclinepi.2015.09.001
31. Welch V, Petticrew M, Tugwell P, et al. PRISMA-Equity 2012 Extension: Reporting Guidelines for Systematic Reviews with a Focus on Health Equity. *PLOS Medicine* 2012;9(10):e1001333. doi: 10.1371/journal.pmed.1001333
32. Ray P, Trisha G, Gill H, et al. Realist review - a new method of systematic review designed for complex policy interventions. *Journal of Health Services Research & Policy* 2005;10(1_suppl):21-34. doi:10.1258/1355819054308530
33. Anderson LM, Petticrew M, Rehfuess E, et al. Using logic models to capture complexity in systematic reviews. *Research Synthesis Methods* 2011;2(1):33-42. doi: 10.1002/jrsm.32
34. Kneale D, Thomas J, Harris K. Developing and Optimising the Use of Logic Models in Systematic Reviews: Exploring Practice and Good Practice in the Use of Programme Theory in Reviews. *PLOS ONE* 2015;10(11):e0142187. doi: 10.1371/journal.pone.0142187
35. Adam T. Advancing the application of systems thinking in health. *Health Research Policy and Systems* 2014;12(1):50. doi: 10.1186/1478-4505-12-50
36. Rutter H, Savona N, Glonti K, et al. The need for a complex systems model of evidence for public health. *The Lancet*;390(10112):2602-04. doi: 10.1016/S0140-6736(17)31267-9
37. Raine R FR, Barratt H, et al. . Challenges, solutions and future directions in the evaluation of service innovations in health care and public health. *Health Services and Delivery Research* 2016;4(16) doi: 10.3310/hsdr04160
38. Kilbourne AM, Switzer G, Hyman K, et al. Advancing Health Disparities Research Within the Health Care System: A Conceptual Framework. *American Journal of Public Health* 2006;96(12):2113-21. doi: 10.2105/AJPH.2005.077628
39. Baker A. BC, Carr D., Connolly AM., Heasman M., Johnson C., Laurent C., Tran A. Reducing health inequalities: system, scale and sustainability. London: Public Health England: PHE publications

For peer review only - http://bmjopen.bmj.com/site/about/guidelines.xhtml

gateway number: 2017226, Crown copyright. Contains information licenced under the Open Government Licence v3.0, 2017.
40. Benach J, Malmusi D, Yasui Y, et al. A new typology of policies to tackle health inequalities and scenarios of impact based on Rose's population approach. *Journal of Epidemiology and Community Health* 2013;67(3):286-91. doi: 10.1136/jech-2011-200363
41. Bambra CL, Hillier FC, Moore HJ, et al. Tackling inequalities in obesity: a protocol for a systematic review of the effectiveness of public health interventions at reducing socioeconomic inequalities in obesity among adults. *Systematic Reviews* 2013;2(1):27. doi: 10.1186/2046-4053-2-27
42. Whitehead M, Dahlgren G. What can be done about inequalities in health? *The Lancet* 1991;338(8774):1059-63. doi: https://doi.org/10.1016/0140-6736(91)91911-D
43. Asaria M, Ali S, Doran T, et al. How a universal health system reduces inequalities: lessons from England. *Journal of Epidemiology and Community Health* 2016;70(7):637-43. doi: 10.1136/jech-2015-206742
44. Wenzl M, McCuskee S, Mossialos E. Commissioning for equity in the NHS: rhetoric and practice. *British Medical Bulletin* 2015;115(1):5-17. doi: 10.1093/bmb/ldv031
45. Dahlgren G, Whitehead, M. Policies and Strategies to Promote Social Equity in Health. Stockholm: Institute for Futures Studies 1991.
46. Lorna A, Mark L, Vikki E, et al. A Systematic Review of the Effectiveness of Health Service Interventions Aimed at Reducing Inequalities in Health. *Journal of Health Services Research & Policy* 1996;1(2):93-103. doi: 10.1177/135581969600100207
47. Geraint L, Sheringham J, Lopez Bernal J, et al. Mastering public health: a postgraduate guide to examinations and revalidation. Second ed. Great Britain: Boca Raton : CRC Press, Taylor & Francis Group 2015.
48. Campbell M, Katikireddi SV, Hoffmann T, et al. TIDieR-PHP: a reporting guideline for population health and policy interventions. *BMJ* 2018;361:k1079. doi: 10.1136/bmj.k1079
49. Sowden S, Nezafat Maldonado B, Beyer F, Cookson R, Thomson R, Lambert M, Bambra C. Reducing inequalities in avoidable hospital admissions: a systematic review. PROSPERO 2019 CRD42019153666 Available from: https://www.crd.york.ac.uk/prospero/display_record.php?ID=CRD42019153666 (accessed 30 Oct 2019)
50. Billings JW, Weinick RM Monitoring the Health Care Safety Net Book II: A Data Book for States and Counties 2003 https://permanent.access.gpo.gov/lps48692/databk2.pdf (accessed 26 Feb 2020)
51. Health and Social Care Information Centre, CCG Outcome Indicator 3.1: Emergency admissions for acute conditions that should not usually require hospital admission - technical specification, 2018, England.
52. Health and Social Care Information Centre, CCG Outcome Indicator 2.6: Unplanned hopsitalisation for chronic ambulatory care senstive conditions, 2018, England.
53. Agency for Healthcare Research and Quality, Guide to Prevention Quality Indicators: Hospital Admission for Ambulatory Care Sensitive Conditions USA: Department of Health and Human Services, Agency for Healthcare Resaearch and Quality; 2001 https://www.ahrq.gov/downloads/pub/ahrqqi/pqiguide.pdf (accessed 26 Feb 2020)
54. NHS England CCG Improvement and Assessment Framework (CCG IAF) 2017/18: Technical annex (version 1.1). London: NHS England; 2017.
55. EPPI-Centre, Social Science Research Unit, UCL Institute of Education, University of London, UK. Eppi-Reviewer 4 systematic review software, Available from: https://eppi.ioe.ac.uk/CMS/Default.aspx?alias=eppi.ioe.ac.uk/cms/er4& (accessed 30 Oct 2019)
56. OECD, OECD Health System Characeristics survey, 2016, https://qdd.oecd.org/subject.aspx?Subject=hsc (accessed 13 Feb 2020)
57. Hoffmann TC, Glasziou PP, Boutron I, et al. Better reporting of interventions: template for intervention description and replication (TIDieR) checklist and guide. *BMJ : British Medical Journal* 2014;348:g1687. doi: 10.1136/bmj.g1687

For peer review only - http://bmjopen.bmj.com/site/about/guidelines.xhtml

58. Thomas BH, Ciliska D, Dobbins M, Micucci S. Effective Public Health Practice Proect: Quality Assessment tool for quantitative studies: McMaster University school of nursing https//:merst.ca/ephpp (accessed 11 Oct 2019).
59. Ogilvie D, Adams J, Bauman A, et al. Using natural experimental studies to guide public health action: turning the evidence-based medicine paradigm on its head. *Journal of Epidemiology and Community Health* 2019:jech-2019-213085. doi: 10.1136/jech-2019-213085
60. Prady SL, Uphoff EP, Power M, et al. Development and validation of a search filter to identify equity-focused studies: reducing the number needed to screen. *BMC Medical Research Methodology* 2018;18(1):106. doi: 10.1186/s12874-018-0567-x

For peer review only

For peer review only - http://bmjopen.bmj.com/site/about/guidelines.xhtml

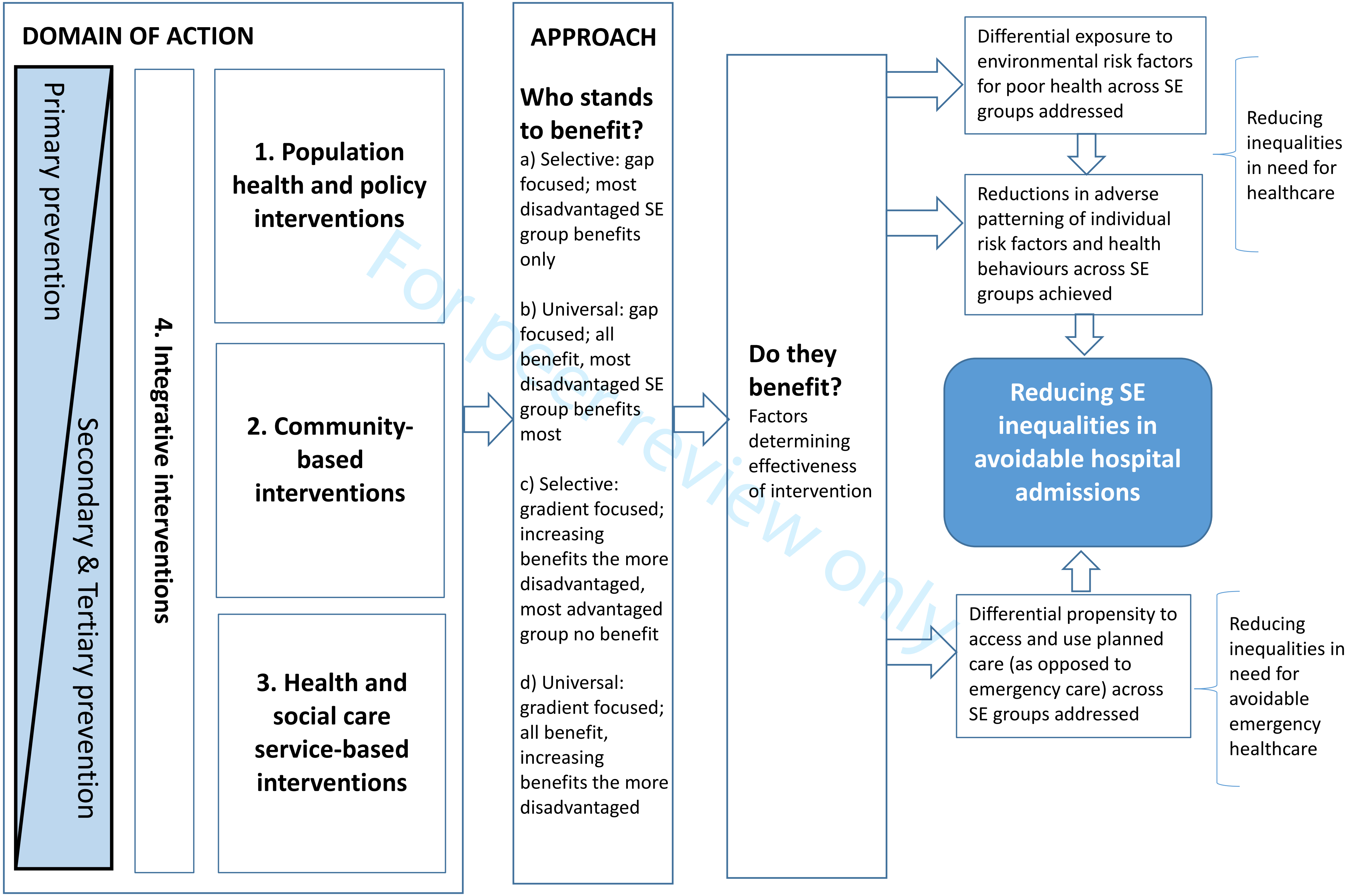

For peer review only

Reporting checklist for protocol of a systematic review.

Based on the PRISMA-P guidelines.

Moher D, Shamseer L, Clarke M, Ghersi D, Liberati A, Petticrew M, Shekelle P, Stewart LA. Preferred Reporting Items for Systematic Review and Meta-Analysis Protocols (PRISMA-P) 2015 statement. Syst Rev. 2015;4(1):1.

		Reporting Item	Page Number
**Title**			
Identification	#1a	Identify the report as a protocol of a systematic review	1
Update	#1b	If the protocol is for an update of a previous systematic review, identify as such	N/A
**Registration**			
	#2	If registered, provide the name of the registry (such as PROSPERO) and registration number	2
**Authors**			
Contact	#3a	Provide name, institutional affiliation, e-mail address of all protocol authors; provide physical mailing address of corresponding author	1
Contribution	#3b	Describe contributions of protocol authors and identify the guarantor of the review	12
**Amendments**			
	#4	If the protocol represents an amendment of a previously completed or published protocol, identify as such and list changes; otherwise, state plan for documenting important protocol amendments	N/A
**Support**			
Sources	#5a	Indicate sources of financial or other support for the review	12

Sponsor	#5b	Provide name for the review funder and / or sponsor	12
Role of sponsor or funder	#5c	Describe roles of funder(s), sponsor(s), and / or institution(s), if any, in developing the protocol	12
**Introduction**			
Rationale	#6	Describe the rationale for the review in the context of what is already known	3-6
Objectives	#7	Provide an explicit statement of the question(s) the review will address with reference to participants, interventions, comparators, and outcomes (PICO)	7
**Methods**			
Eligibility criteria	#8	Specify the study characteristics (such as PICO, study design, setting, time frame) and report characteristics (such as years considered, language, publication status) to be used as criteria for eligibility for the review	7-8
Information sources	#9	Describe all intended information sources (such as electronic databases, contact with study authors, trial registers or other grey literature sources) with planned dates of coverage	9
Search strategy	#10	Present draft of search strategy to be used for at least one electronic database, including planned limits, such that it could be repeated	Suppl file 2
Study records - data management	#11a	Describe the mechanism(s) that will be used to manage records and data throughout the review	10
Study records - selection process	#11b	State the process that will be used for selecting studies (such as two independent reviewers) through each phase of the review (that is, screening, eligibility and inclusion in meta-analysis)	9
Study records - data collection process	#11c	Describe planned method of extracting data from reports (such as piloting forms, done independently, in duplicate), any processes for obtaining and confirming data from investigators	9-10

For peer review only

Data items	#12	List and define all variables for which data will be sought (such as PICO items, funding sources), any pre-planned data assumptions and simplifications	7-8
Outcomes and prioritization	#13	List and define all outcomes for which data will be sought, including prioritization of main and additional outcomes, with rationale	7-8
Risk of bias in individual studies	#14	Describe anticipated methods for assessing risk of bias of individual studies, including whether this will be done at the outcome or study level, or both; state how this information will be used in data synthesis	10
Data synthesis	#15a	Describe criteria under which study data will be quantitatively synthesised	10-11
Data synthesis	#15b	If data are appropriate for quantitative synthesis, describe planned summary measures, methods of handling data and methods of combining data from studies, including any planned exploration of consistency (such as I2, Kendall's τ)	10-11
Data synthesis	#15c	Describe any proposed additional analyses (such as sensitivity or subgroup analyses, meta-regression)	11
Data synthesis	#15d	If quantitative synthesis is not appropriate, describe the type of summary planned	10-11
Meta-bias(es)	#16	Specify any planned assessment of meta-bias(es) (such as publication bias across studies, selective reporting within studies)	10-11
Confidence in cumulative evidence	#17	Describe how the strength of the body of evidence will be assessed (such as GRADE)	10-11

Notes:

- 10: Suppl file 2 The PRISMA-P checklist is distributed under the terms of the Creative Commons Attribution License CC-BY 4.0. This checklist was completed on 31. October 2019 using https://www.goodreports.org/, a tool made by the EQUATOR Network in collaboration with Penelope.ai

For peer review only

Supplementary file 2 – preliminary MEDLINE search strategy

Element	Search items
Hospital admissions [AND]	Hospitals/statistics & numerical data or Emergency Service, Hospital/statistics & numerical data* or Hospitalization/ec, td [Economics, Trends] or Patient Readmission/ or Patient Admission"/ec, td [Economics, Trends] or Emergency hospital admission*.ti,ab or emergency hospitali#ation.ti,ab or unplanned hospitali#ation.ti,ab or (overnight stay adj5 admission*).ti,ab or (primary care adj5 admission*).ti,ab or (ambulatory care adj5 admission*).ti,ab.or (admission* adj5 emergenc*).ti,ab.or (Emergency Treatment adj5 admission*).ti,ab.or . ((emergency care adj5 admission*) or readmission*).mp or (emergency room adj5 admission*).ti,ab. or emergency admission*.mp.or overnight stay.mp. or emergency medical admission*.mp.or (hospital* adj5 readmission rates).mp.or ((unscheduled or unplanned or un-planned or unanticipated or unexpected) adj5 (admission* or readmission* or hospitali#ation or care)).mp or (admissions adj5 hospital days).mp.or . (hospital admission* adj5 emergenc*).mp.
Equity [AND}	Combined equity filter MEDLINE – see additional file 1[1]
Intervention [AND]	exp."Health Care Quality, Access.mp. or Health Care.mp. or exp "Delivery of Health Care"/ or Chronic Disease/therapy* or State Medicine/statistics & numerical data or programme evaluation.mp or intervention$.tw. or evaluat$.tw. or policy.tw or strategy.tw. or access to care.mp.or access to services.mp. or scheme.tw or program*.tw or initaitve.tw or ambulatory care/ or aftercare/ or after-hours care/ or case management/ or "continuity of patient care"/ or patient education/ or health services accessibility/ or "deliver of health care"/ or Home Care Services, Hospital-Based/ or Managed Care Program*/ or Health Knowledge, Attitudes, Practice/ or *"Outcome Assessment (Health Care)"/ or "Drug Utili$ation Review"/ or Intermediate Care Facvilities/ or *"Self Care"/ or Community Health Services/ or "Patient Discharge"/ or Health Services Research/ or Primary Health Care/ or Physicians, Family/ or Treatment Outcome/ or Risk Assessment/ or Telemedicine/ or Quality of care.mp. or (out-of-hours or OOH).mp. or access to care.mp or access to services.mp or continuity of care.mp or medication review.mp or organisation of care.mp or outreach.mp or community matron.mp or walk in centres.mp or telemonitoring.mp or "hospital at home".mp or "virtual wards".mp or self management.mp or assessment units.mp or observation wards.mp or GPS in A&E.mp or ddischarge plan*.or mp primary care.mp or telephone follow-up.mp or home telecare.mp or on-line health.mp (e0health or ehealth).mp or home telemedicine.mp or general practitioner*.mp or health Promotion/ or health promotion.ti,ab. or health behaviour.ti,ab. or health behavior.ti,ab. or (policy and (social or public or urban or environmental or fiscal)).ti,ab. or urban planning.ti,ab. or city planning.ti,ab. or built environment.ti,ab. or social environment.ti,ab. or physical environment.ti,ab. or cultural environment.ti,ab. or urban environment.ti,ab. or neighbourhood.ti,ab. or community.ti,ab. or societal.ti,ab. or social interventions.ti,ab. or community interventions.ti,ab. or individual level.ti,ab. or lifestyle.ti,ab. or individual.ti,ab. or tax$.ti,ab. or subsid$.ti,ab. or price$.ti,ab. or health education.ti,ab. or social marketing.ti,ab. or (diet and (advice or counselling)).ti,ab. or (exercise and (advice or counselling)).ti,ab. or cash transfer$.ti,ab. or lifestyle counselling.ti,ab. or behavioural counselling.ti,ab. or behavioral counselling.ti,ab. or workplace.ti,ab. or campaign$.ti,ab. or (access adj1 facilities).ti,ab
Study type [AND]	(evaluat$ or effective$ or Intervention or RCT or experiment$ or randomi?ed controlled trial$ or clinical randomi?ed controlled trial$ or cluster randomi?ed controlled trial$ or double blind randomi?ed controlled trial$ or randomi?ed consent design or single blind randomi?ed controlled trial$ or randomi?ed or placebo or random$ or trial or quasi?experiment$ or pre$test or post$test or trial or time series or evaluat$ or intervention$ or "before and after" or intervention$ or commun! ity trial or non?randomi?ed or repeat$ or repeat$ measures).ti,ab. or (exp Clinical Trial/ or exp Randomized Controlled Trial/ or exp Randomization/ or exp Double-Blind Method/ or exp Single-Blind Method/ or exp Cross-Over Studies/) or clinical trial.ti,ab. or latin square.ti,ab. or random$.ti,ab. or exp Evaluation/ or clinical trial.ti,ab. or clinical trial.pt. or (before adj1 after adj1 (stud$ or trial$ or design$)).ti,ab. or random$.ti,ab. or (quasi?experimental or pseudo?experimental).ti,ab. or (nonrandomi?ed or non?randomi?ed or pseudo?randomi?sed or quasi?randomi?ed).ti,ab. or ((population level or population based or population orientated or population oriented or

	community level or community based or community orientated or community oriented) adj3 (intervention$ or prevention or policy or policies or program$ or project$)).ti,ab.
OECD countries only [NOT]	(Algeria$ or Egypt$ or Liby$ or Morocc$ or Tunisia$ or Western Sahara$ or Angola$ or Benin or Botswana$ or Burkina Faso or Burundi or Cameroon or Cape Verde or Central African Republic or Chad or Comoros or Congo or Djibouti or Eritrea or Ethiopia$ or Gabon or Gambia$ or Ghana or Guinea or Keny$ or Lesotho or Liberia or Madagasca$ or Malawi or Mali or Mauritania or Mauritius or Mayotte or Mozambiq$ or Namibia$ or Niger or Nigeria$ or Reunion or Rwand$ or Saint Helena or Senegal or Seychelles or Sierra Leone or Somalia or South Africa$ or Sudan or Swaziland or Tanzania or Togo or Ugand$ or Zambia$ or Zimbabw$ or China or Chinese or Hong Kong or Macao or Mongolia$ or Taiwan$ or Belarus or Moldov$ or Russia$ or Ukraine or Afghanistan or Armenia$ or Azerbaijan or Bahrain or Cyprus or Cypriot or Georgia$ or Iran$ or Iraq$ or Israel$ or Jordan$ or Kazakhstan or Kuwait or Kyrgyzstan or Leban$ or Oman or Pakistan$ or Palestin$ or Qatar or Saudi Arabia or Syria$ or Tajikistan or Turkmenistan or United Arab Emirates or Uzbekistan or Yemen or Bangladesh$ or Bhutan or British Indian Ocean Territory or Brunei Darussalam or Cambodia$ or India$ or Indonesia$ or Lao or People's Democratic Republic or Malaysia$ or Maldives or Myanmar or Nepal or Philippin$ or Singapore or Sri Lanka or Thai$ or Timor Leste or Vietnam or Albania$ or Andorra or Bosnia$ or Herzegovina$ or Bulgaria$ or Croatia$ or Estonia or Faroe Islands or Greenland or Liechtenstein or Lithuani$ or Macedonia or Malta or maltese or Romania or Serbia$ or Montenegro or Slovenia or Svalbard or Argentina$ or Belize or Bolivia$ or Brazil$ or chile or Chilean or Colombia$ or Costa Rica$ or Cuba or Ecuador or El Salvador or French Guiana or Guatemala$ or Guyana or Haiti or Honduras or Jamaica$ or Nicaragua$ or Panama or Paraguay or Peru or Puerto Rico or Suriname or Uruguay or Venezuela or developing countr$ or south America$).ti,sh. [mp=title, abstract, original title, name of substance word, subject heading word, floating sub-heading word, keyword heading word, organism supplementary concept word, protocol supplementary concept word, rare disease supplementary concept word, unique identifier, synonyms]
Filter applied	Humans only
Filter applied	1999-2019

1. Prady SL, Uphoff EP, Power M, et al. Development and validation of a search filter to identify equity-focused studies: reducing the number needed to screen. *BMC Medical Research Methodology* 2018;18(1):106. doi: 10.1186/s12874-018-0567-x

For peer review only

Manuscript ID bmjopen-2019-035429, entitled "Reducing inequalities in avoidable hospital admissions: explanatory framework and systematic review protocol."

DATA EXTRACTION FORM

This is an example of the data extraction form that we will use in the systematic review.
This process will be done in EPPI-reviewer[1]

**STUDY DETAILS**	
Author	
Publication year	
Funding source	
Country	e.g. USA, Mexico, etc.
Further location details / study setting details	e.g. city, state, region etc.
Health care system characteristics	
Most common type health coverage*<br><br>*from latest OECD survey of health system characteristics	☐ autonomic coverage<br>☐ compulsory coverage,<br>☐ voluntary coverage<br>☐ no coverage
Study period	e.g. 3 years
Study type<br>(tick the appropriate option)	☐ Randomised controlled trials<br>☐ Non-randomised or uncontrolled trials<br>☐ Prospective and retrospective cohort studies (with and/or without control groups)<br>☐ Prospective repeat cross-sectional (panel) studies (with and/or with control groups)<br>☐ Ecological studies<br>☐ Economic evaluation<br>☐ Other (Please specify): ______________
Description of study methodology	
Confounders<br>*Other variables controlled for/consider in the analysis*	e.g. sex, ethnicity, age etc.
Blinding<br>*Details of this was done*	☐ No<br>☐ Yes (details): ______________________________<br>☐ N/A
**POPULATION**	
Age range	e.g. adults aged 18 and over
number of included participants/areas	
Clustering	e.g. individuals in areas etc.
Gender	☐ Men<br>☐ Women

For peer review only

	☐ Other: ______
General study population characteristics (eligibility criteria for participation)	
Targeted or universal intervention?	☐ Targeted intervention<br>☐ Universal intervention
If targeted, explanation of target group	e.g. homeless groups, those in receipt of Medicaid, etc.
If universal, description of subdivision of outcome by SES. *What measure/marker of SES used and what subdivision made.*	e.g. most deprived quintile/decile of population vs rest
Comparison group	e.g. another city, same group before and after, or no comparison etc
**INTERNVETION**	
Intervention Approach** *Please see definitions of each intervention below*	☐ Population health and policy intervention<br>☐ Community based intervention<br>☐ Health and social care service<br>☐ Integrative intervention
Description of intervention *(programme, policy, strategy, initiative, scheme or activity)*	e.g. fluoridation of drinking water, out of hospital support package, etc.
Details of provider of the intervention	e.g. national health provider, local government, private entity, etc.
Were there variations in the intervention between target groups?	☐ No<br>☐ Yes
Who provided the intervention?	e.g. nurse, doctor, social worker, etc.
How was the intervention delivered?	e.g. telephone, face to face, etc.
Intervention date/period	
Other variables e.g. time	
**OUTCOME**	
Outcome reported in study	☐ Hospital admission/hospitalisation<br>☐ Hospital readmission<br>☐ Emergency hospital admission<br>☐ Avoidable hospital admission

For peer review only

	☐ Unspecified<br>Definition____
Unit of analysis	e.g. Individual patient, lower super output area/neighbourhood, local authority, district etc.
Global or condition specific?	☐ Global<br>☐ Specific condition: ____________________
Detailed description of outcome variable	e.g. admission for complication of diabetes in patents with existing diagnosis of diabetes.
Justification for avoidable classification	e.g. One of ICD10 codes included in list of ambulatory care sensitive conditions
**Outcome Measure:**	
For the targeted interventions…	
No. of admission in intervention group	
Rate of intervention group admissions in Intervention group	
No. of admissions in control group if applicable	
Rate of admissions in control group if applicable	
Has sub-analysis of the outcome by SES and then by e.g. ethnicity, gender etc. been reported? And figures here.	☐ Yes (other variables)____________________<br>☐ No<br>☐ Data______________________________
For universal interventions:	
What measure of SES is used?	e.g. IMD 2015 quintile of deprivation
No. of admission across SES groups	
Rate of admissions across SES groups	
OR across SES groups	
RR	
SMD	
Pooled proportions	
Measure of gradient	e.g. absolute gradient of inequality, gini coefficient etc
Other measures	
Has sub-analysis of SES and then breakdown by other variables	☐ Yes (over variables):________<br>☐ No<br>☐ Data_____________________

For peer review only

been undertaken (e.g. ethnicity, gender)	
Outcome measures in control group *if applicable*	
Who benefits the most?	☐ Targeted intervention to those in most disadvantaged socioeconomic group only, focused on closing the health gap (selective: gap focused)<br>☐ Universal intervention, with additional focus on those in the most disadvantaged socioeconomic group to close the health gap (universal: gap focused).<br>☐ Redistributive intervention, a universally applied intervention but where the extent of benefit increases across the social gradient, such that the most socioeconomically advantaged are not expected to benefit at all from the intervention due to lack of need for it (selective: gradient focused).<br>☐ Proportionate universalism, universal intervention benefitting all, but with increasing benefits of intervention across the social gradient (universal: gradient focused).
Evidence for above statement	Interpretation of e.g. ORs etc. extracted
Comments/Additional relevant info	
**IMPLEMENTION**<br>*This information will be useful for assessing generalisability of findings/validity of pooling studies to do metanalysis. Details will be sought from the manuscripts and any associated papers through a linked citation search*	
Date of implementation	
Delivery dates	
Theoretical underpinning for intervention:	
Implementation context:	
Members of intervention team	
Experience level of the intervention team (planners and implementers)	
Consultation and/or collaboration processes (planning and delivery stages)	
Resources (for example time, money, staff and equipment)	
The TIDieR[52] or TIDieR-PHP[47] checklists will be used to extract further relevant information<br>*See checklists below*<br>.	

For peer review only

1) EPPI-Centre, Social Science Research Unit, UCL Institute of Education, University of London, UK. Eppi-Reviewer 4 systematic review software, Available from: https://eppi.ioe.ac.uk/CMS/Default.aspx?alias=eppi.ioe.ac.uk/cms/er4& (accessed 30 Oct 2019)

**Intervention Approaches

Population health and policy intervention

Legal, fiscal, structural, organisational, environmental, and policy interventions that seek to change health related behaviours or to modify the social and economic determinants of health. This includes interventions with other goals that bring about such changes as a by product.

Community-based intervention
Community resources and assets, for example local area coordination initiatives.

Health and social care service
In this context these might include interventions considered in previous reviews of avoidable admissions (for example, disease and medication management and education programmes, vaccination, structured discharge planning, comprehensive geriatric assessment, virtual wards, hospital at home initiatives) as well as primary care (provision, access, quality, continuity), benchmarking, predictive risk-modelling and case-management interventions.

Integrative intervention
Interventions that create greater synergy and closer working between domains 1 – 3 or within 3 (between for example, primary and secondary healthcare and social care). For example, social prescribing initiatives facilitate closer working between domains 2 and 3.

For peer review only

Campbell M, Katikireddi SV, Hoffmann T, et al. TIDieR-PHP: a reporting guideline for population health and policy interventions. *BMJ* 2018;361:k1079.

**Item**	**Item description**	**Page in manuscript where item is reported**	**Other***
1 Brief name	Provide the name or a phrase that describes the intervention		
2 Why	Describe the logic, mechanisms, or rationale of the intervention, clearly linking intervention elements to the expected effects on immediate or longer term outcomes (or both)		
3 What materials	Describe any materials used in the intervention (including online appendices or URLs for further details). For example:<br>—informational materials (may include those provided to recipients of the intervention or in training of intervention providers)<br>—nature and value of any benefit provided (eg, cash, voucher, meal)<br>—any physical resources or infrastructure provided as part of the intervention		
4 What and how	Describe how the intervention was planned, established, and intended to be delivered. Depending on the type of intervention, it may be useful to consider:<br>—how sources of funding for the intervention and the service providers were obtained, how users were enrolled and the		

Item	Item description	Page in manuscript where item is reported	Other*
	service delivered<br>—how any payments were made or benefits delivered, how qualifying conditions were implemented<br>—the entity being regulated, the scope of the regulation, permitted level of use; procedures for monitoring or enforcing compliance, and any sanctions for non-compliance<br>—how people were exposed to the intervention, whether it was provided to individuals or larger populations<br>—any underpinning legislation including name, date passed, and legislative body		
5 Who provided	Describe the provider of the intervention, including legal status and powers, field organisations and staff responsible for planning, implementation, monitoring and enforcement. Where relevant, describe intervention provider expertise and training (general or specific to the intervention)		
6 Where	Describe the type of location (eg, school, community centre) and the geographical scope of the intervention (eg, national, regional, city-wide). Where relevant, describe the historical, cultural, socioeconomic,		

**Item**	**Item description**	**Page in manuscript where item is reported**	**Other***
	or political background to the intervention		
7 When and how often	Describe when the intervention was implemented, how long it remained in place, and, if applicable, the number, duration, and scheduling of occasions		
8.1 Planned variation	Describe and provide the reason for any variation or tailoring that was planned or allowed for in the design of the intervention. Examples include differences between locations, geographical areas, population subgroups, or over time		
8.2 Unplanned variation	Describe and provide the reason for any unplanned variation or modifications in the intervention (eg, between different locations, geographical areas, population subgroups, or over time) that were made after the intervention commenced		
9.1 How well	Describe any strategies used or actions taken to maintain fidelity of the intervention (ie, to ensure that the intervention was delivered as intended)		
9.2 How well—delivery	Describe the fidelity of the intervention (ie, the extent to which the intervention was delivered as intended)		

- *If the information is not provided in the primary paper, give details of where this information is available (eg, protocol, other published papers (provide citation details), or a website (provide the URL))

Hoffmann TC, Glasziou PP, Boutron I, et al. Better reporting of interventions: template for intervention description and replication (TIDieR) checklist and guide. *BMJ : British Medical Journal* 2014;348:g1687. doi: 10.1136/bmj.g1687

Item No	Item	Pg no. in manuscript where reported
**Brief name**		
1	Provide the name or a phrase that describes the intervention	
**Why**		
2	Describe any rationale, theory, or goal of the elements essential to the intervention	
**What**		
3	Materials: Describe any physical or informational materials used in the intervention, including those provided to participants or used in intervention delivery or in training of intervention providers. Provide information on where the materials can be accessed (such as online appendix, URL)	
4	Procedures: Describe each of the procedures, activities, and/or processes used in the intervention, including any enabling or support activities	
**Who provided**		
5	For each category of intervention provider (such as psychologist, nursing assistant), describe their expertise, background, and any specific training given	
**How**		
6	Describe the modes of delivery (such as face to face or by some other mechanism, such as internet or telephone) of the intervention and whether it was provided individually or in a group	
**Where**		
7	Describe the type(s) of location(s) where the intervention occurred, including any necessary infrastructure or relevant features	
**When and How Much**		
8	Describe the number of times the intervention was delivered and over what period of time including the number of	

For peer review only

Item No	Item	Pg no. in manuscript where reported
	sessions, their schedule, and their duration, intensity, or dose	
**Tailoring**		
9	If the intervention was planned to be personalised, titrated or adapted, then describe what, why, when, and how	
**Modifications**		
10	If the intervention was modified during the course of the study, describe the changes (what, why, when, and how)	
**How well**		
11	Planned: If intervention adherence or fidelity was assessed, describe how and by whom, and if any strategies were used to maintain or improve fidelity, describe them	
12	Actual: If intervention adherence or fidelity was assessed, describe the extent to which the intervention was delivered as planned	

For peer review only

BMJ Open

Interventions to reduce inequalities in avoidable hospital admissions: explanatory framework and systematic review protocol

Journal:	*BMJ Open*
Manuscript ID	bmjopen-2019-035429.R2
Article Type:	Protocol
Date Submitted by the Author:	02-Apr-2020
Complete List of Authors:	Sowden, Sarah; Newcastle University, Population Health Sciences Institute<br>Nezafat-Maldonado, Behrouz; Newcastle University, Population Health Sciences Institute<br>Wildman, Josephine; Newcastle University, Population Health Sciences Institute<br>Cookson, Richard; University of York, Centre for Health Economics<br>Thomson, Richard; Newcastle University, Population Health Sciences Institute<br>Lambert, Mark; Public Health England, North East Centre<br>Beyer, Fiona; Newcastle University, Population Health Sciences Institute<br>Bambra, Clare; Newcastle University, Population Health Sciences Institute
**Primary Subject Heading**:	Public health
Secondary Subject Heading:	Health policy, Health services research
Keywords:	PREVENTIVE MEDICINE, PUBLIC HEALTH, Health policy < HEALTH SERVICES ADMINISTRATION & MANAGEMENT, Quality in health care < HEALTH SERVICES ADMINISTRATION & MANAGEMENT, Organisation of health services < HEALTH SERVICES ADMINISTRATION & MANAGEMENT, ACCIDENT & EMERGENCY MEDICINE

SCHOLARONE™
Manuscripts

I, the Submitting Author has the right to grant and does grant on behalf of all authors of the Work (as defined in the below author licence), an exclusive licence and/or a non-exclusive licence for contributions from authors who are: i) UK Crown employees; ii) where BMJ has agreed a CC-BY licence shall apply, and/or iii) in accordance with the terms applicable for US Federal Government officers or employees acting as part of their official duties; on a worldwide, perpetual, irrevocable, royalty-free basis to BMJ Publishing Group Ltd ("BMJ") its licensees and where the relevant Journal is co-owned by BMJ to the co-owners of the Journal, to publish the Work in this journal and any other BMJ products and to exploit all rights, as set out in our licence.

The Submitting Author accepts and understands that any supply made under these terms is made by BMJ to the Submitting Author unless you are acting as an employee on behalf of your employer or a postgraduate student of an affiliated institution which is paying any applicable article publishing charge ("APC") for Open Access articles. Where the Submitting Author wishes to make the Work available on an Open Access basis (and intends to pay the relevant APC), the terms of reuse of such Open Access shall be governed by a Creative Commons licence – details of these licences and which Creative Commons licence will apply to this Work are set out in our licence referred to above.

Other than as permitted in any relevant BMJ Author's Self Archiving Policies, I confirm this Work has not been accepted for publication elsewhere, is not being considered for publication elsewhere and does not duplicate material already published. I confirm all authors consent to publication of this Work and authorise the granting of this licence.

For peer review only

Interventions to reduce inequalities in avoidable hospital admissions: explanatory framework and systematic review protocol

Dr Sarah Sowden
https://orcid.org/0000-0001-9359-3463
sarah.sowden@newcastle.ac.uk
+44 (0)1912083410

Population Health Sciences Institute
4th Floor, Baddiley-Clark Building
Newcastle University
Newcastle upon Tyne
NE2 4AX

Dr Behrouz Nezafat Maldonado behrouz.nezafat-maldonado@ncl.ac.uk Population Health Sciences Institute, Newcastle University, Newcastle upon Tyne, UK.
Dr Josephine Wildman josephine.wildman@newcastle.ac.uk Population Health Sciences Institute, Newcastle University, Newcastle upon Tyne, UK
Professor Richard Cookson richard.cookson@york.ac.uk Centre for Health Economics, York University, York, UK.
Professor Richard Thomson richard.thomson@ncl.ac.uk Population Health Sciences Institute, Newcastle University, Newcastle upon Tyne, UK.
Dr Mark Lambert mark.lambert2@nhs.net Public Health England (North East Centre), Newcastle upon Tyne, UK.
Dr Fiona Beyer fiona.beyer@ncl.ac.uk Population Health Sciences Institute, Newcastle University, Newcastle upon Tyne, UK.
Professor Clare Bambra clare.bambra@ncl.ac.uk Population Health Sciences Institute, Newcastle University, Newcastle upon Tyne, UK.

For peer review only

For peer review only - http://bmjopen.bmj.com/site/about/guidelines.xhtml

ABSTRACT

Introduction

Internationally there is pressure to contain costs due to rising numbers of hospital admissions. Alongside age, socioeconomic disadvantage is the strongest risk factor for avoidable hospital admission. This equity-focused systematic review is required for policy makers to understand what has been shown to work to reduce inequalities in hospital admissions, what does not work, and where the current gaps in the evidence-base are.

Methods and analysis

An initial framework shows how interventions are hypothesised to reduce socioeconomic inequalities in avoidable hospital admissions. Studies will be included if the intervention focuses exclusively on socioeconomically disadvantaged populations or if the study reports differential effects by socioeconomic status (education, income, occupation, social class, deprivation, poverty, or an area-based proxy for deprivation derived from place of residence) with respect to hospital admission or readmission (overall or condition-specific for those classified as ambulatory care sensitive).

Studies involving individuals of any age, undertaken in OECD countries, published from 2000 to 29 February 2020 in any language will be included. Electronic searches will include MEDLINE, Embase, CINAHL, Cochrane CENTRAL and the Web of Knowledge platform. Electronic searches will be supplemented with full citation searches of included studies, website searches and retrieval of relevant unpublished information.

Study inclusion, data extraction and quality appraisal will be conducted by two reviewers. Narrative synthesis will be conducted and also meta-analysis where possible. The main analysis will examine the effectiveness of interventions at reducing socioeconomic inequalities in hospital admissions. Interventions will be characterised by their domain of action and approach to addressing inequalities. For included studies, contextual information on where, for whom and how these interventions are organised, implemented and delivered will be examined where possible.

Ethics and dissemination

Ethical approval was not required for this protocol. The research will be disseminated via peer-reviewed publication, conferences, and an open-access policy-orientated paper.

PROSPERO registration number: CRD42019153666

STRENGTHS AND LIMITATIONS OF THIS STUDY

- The study employs a rigorous international gold-standard methodology (PRISMA-E) to undertake an equity-focused systematic review and therefore will provide a comprehensive overview of existing literature on interventions that reduce socioeconomic inequalities in avoidable hospital admissions.
- By following PRISMA-E guidelines this study includes a framework to explain how interventions might reduce inequalities in hospital admissions which will be useful to decision makers.

For peer review only - http://bmjopen.bmj.com/site/about/guidelines.xhtml

- The review adopts a newly developed and validated comprehensive search strategy for identifying studies focused on equity issues and is therefore at the forefront of developments in equity-based systematic reviewing practice.
- One limitation is that we will not conduct full-text hand-searching and so may miss studies which have not made any reference to equity-related terms in either the title or abstract.
- Another limitation is that studies may be too heterogeneous to obtain combined effect estimates through meta-analysis.

KEYWORDS

Healthcare Disparities, Health Equity, Socioeconomic factors, Hospitalization, Systematic review

INTRODUCTION

Due to pervasive socioeconomic inequalities in health[1], people in disadvantaged neighbourhoods are more in need of care. Often, however, they have worse access to, experience of, and outcomes from healthcare – even in universal health systems[2]. Potentially avoidable hospital admission for chronic conditions are an international concern and, for example, account for over 37 million bed days each year across the European Union (EU)[3]. Social and economic inequality is a likely contributor to such admissions - in all countries regardless of the health system financing scheme, population characteristics or coverage rates. Alongside age, socioeconomic disadvantage is known to be the strongest risk factor for avoidable hospital admission[4]. To take England as an example, in 2015 263,894 excess avoidable emergency admissions were associated with socioeconomic inequality[5] and excess hospitalisations associated with socioeconomic inequality cost the NHS £4.8 billion per year[6]. Reducing pressures on hospital services therefore requires addressing the socioeconomic differences in health risks and behaviours that give rise to inequalities in avoidable hospital admissions[7].

Different ethnic groups experience different avoidable admission rates, and the interrelationship between ethnicity and socioeconomic status is complex and context specific[8]. There are national variations in rates of avoidable admissions[3] as well as local variation in avoidable admission socioeconomic inequality[9,10] in Organisation for Economic Co-operation and Development (OECD) countries.

Policy context

There is growing pressure to contain costs and prevent disruptions in elective hospital care due to a rising number of hospital admissions across OECD countries[11]. Getting health and social care systems working better together to reduce avoidable admissions[7 12 13] is a common, key priority in diverse health systems[14 15]. Addressing inequalities and improving the integration of primary and secondary services has a high policy profile in the UK[16]and internationally[17]. For example, WHO Europe Health 2020 framework aims to improve health for all and reduce health inequalities through investment in health, tacking major disease burdens, strengthening people-centred health systems and public health capacity and creating supportive environments and resilient communities[18].

Some countries have explicit statutory duties to address health and healthcare inequalities. In Spain, the Royal Decree Law 7/2018[19], recognises access to the National Health System as a fundamental right of every person in Spain. In addition, Spanish health regulations such as The General Health Act (1986)[20] and the National Health System Cohesion and Quality Act (2003)[21] aim to overcome health inequalities and guarantee equality of access to public health care services. Similarly, the Health and Social Care Act 2012[22] in England mandates each local area in England to take steps to address

For peer review only - http://bmjopen.bmj.com/site/about/guidelines.xhtml

inequalities in healthcare outcomes and tackling health inequalities is seen as critical to ensuring the long term sustainability of the UK national health service[23].

There are promising interventions to reduce avoidable hospital admissions in areas including education, self-management, rehabilitation and telemedicine[13]. However, existing systematic reviews have examined *only* the effects of interventions on reducing levels of hospital admissions *overall*, as opposed to the effects on *inequalities* in avoidable hospital admissions. The differential effectiveness of interventions across socio-economic groups in unknown, including a lack of evidence on any interventions aimed at reducing inequalities or targeted in their delivery to disadvantaged groups[4 13].

It is critical for policy-making in this area that evidence of the effectiveness of different types of interventions at tackling inequalities is systematically reviewed. There is currently a lack of accessible policy and practice ready evidence on what works in terms of interventions to reduce inequalities in avoidable hospital admissions. Conversely, there is limited understanding of what interventions should be avoided; frequently well intentioned interventions may increase rather than decrease inequalities[24 25]. The organisation and implementation of such interventions is also important to understand. Little is known about the effectiveness of system wide approaches to reducing avoidable admissions[13] or the impact of wider contextual factors outside the direct control of local health and care provision; real world interventions are rarely implemented in isolation, and the complex interaction of interventions with the particular context in which they are embedded determines outcomes[12 26 27]. Internationally, there is a lack of research on potential explanations for the apparent variation in health-care equality performance between local areas required for those working in policy and practice to learn quality improvement lessons[28].

Against this backdrop, this systematic review will address this deficit in the knowledge base by reviewing primary studies of the effectiveness of interventions in reducing socioeconomic inequalities in avoidable admissions. It will consider interventions (across population health and policy-level, community, service-based and integrative domains of action) which might reduce socioeconomic inequalities in avoidable admissions, highlight any gaps in the evidence base and, for any interventions identified, seek to establish how such interventions are organised, implemented and delivered.

Intervention framework

In line with PRISMA-E[29-31] guidelines, as the first stage of this equity-focused review we have developed a framework[29-34] for how inequalities in avoidable hospital admissions might be tackled (figure 1). The framework outlines the pathways through which interventions are hypothesised to operate to result in reducing socioeconomic inequalities in avoidable hospital admissions over time. This framework has adopted a system-level focus to capture the complexity of place-based working[35-37]. It has been developed from existing frameworks for avoidable admissions[5] and for addressing health and healthcare inequalities[38-42]. The intention is for this to be further revised iteratively[34] as evidence from the systematic review emerges and in consultation with patient, public, and policy and practice partners. In this systematic review the interventions will be grouped according to this framework (with recognition that some interventions may be cross-cutting). The term intervention is used to refer to any programme, policy, strategy, initiative, scheme or activity that has been implemented where either:

a) the outcome of the action taken has been measured in terms of avoidable hospital admission across socioeconomic groups, or

For peer review only - http://bmjopen.bmj.com/site/about/guidelines.xhtml

b) the intervention was focused specifically on socioeconomically disadvantaged areas or groups and where the outcome of the intervention was measured in terms of avoidable hospital admissions in this group.

Tackling socioeconomic inequality in healthcare is challenging[43 44], requiring action on a broad scale[1 2 39 42 45], and health and social care systems working together with other agencies[28 38 39 46] to bring about change. There is increasing recognition amongst policy-makers and commissioners that to tackle complex issues such as avoidable admissions effectively and to reduce inequalities in health and healthcare requires integrated policy, strategy and interventions across different domains of action (population health and policy-level, community-based, health and social care service-based as well as those focused on integrating activity)[2 39]. Based on this understanding, this framework has characterised interventions by their domain of action (1-4) and their approach to tackling inequalities (a-d). There are four broad domains of action[16] and the orientation of activity to primary, secondary and tertiary prevention[47] shifts accordingly across the domains of action (figure 1):

1. Population health and policy-level interventions. Legal, fiscal, structural, organisational, environmental, and policy interventions that seek to change health related behaviours or to modify the social and economic determinants of health[48]. This includes interventions with other goals that bring about such changes as a by product[48].
2. Community-based interventions. Community resources and assets, for example local area coordination initiatives.
3. Health and social care service-based interventions. In this context these might include interventions considered in previous reviews of avoidable admissions[13] (for example, disease and medication management and education programmes, vaccination, structured discharge planning, comprehensive geriatric assessment, virtual wards, hospital at home initiatives) as well as primary care (provision, access, quality, continuity), benchmarking, predictive risk-modelling and case-management interventions.
4. Integrative interventions – interventions that create greater synergy and closer working between domains 1 – 3 or within 3 (between for example, primary and secondary healthcare and social care). For example, social prescribing initiatives facilitate closer working between domains 2 and 3.

According to Benach et al.[40] interventions to address inequalities may adopt one of four different approaches:

a) Targeted intervention to those in most disadvantaged socioeconomic group only, focused on closing the health gap (selective: gap focused).

b) Universal intervention, with additional focus on those in the most disadvantaged socioeconomic group to close the health gap (universal: gap focused).

c) Redistributive intervention, a universally applied intervention but where the extent of benefit increases across the social gradient, such that the most socioeconomically advantaged are not expected to benefit at all from the intervention due to lack of need for it (selective: gradient focused).

d) Proportionate universalism, universal intervention benefitting all, but with increasing benefits of intervention across the social gradient (universal: gradient focused).

For peer review only - http://bmjopen.bmj.com/site/about/guidelines.xhtml

Here, we provide three hypothetical illustrative examples of the application of this framework in the context of reducing socioeconomic inequalities in avoidable hospital admissions. Firstly, there may be a social prescribing intervention (domains 2 and 4) which is facilitated through citizens advice services and serves those who have a limiting long-term condition and are in receipt of unemployment or other welfare benefits (approach c). This intervention is designed, financed and implemented in such a way that it is successful in reducing avoidable hospital admissions for these individuals. Secondly, there may be a water fluoridation intervention (domain 1). This intervention benefits the whole population in terms of reductions in hospitalisations resulting from tooth decay but with increasing benefits across the social gradient as need for the intervention is greatest in lower socioeconomic groups where tooth-health is poorest (approach d). Frequently well intentioned interventions may increase rather than decrease inequalities[24 25]. So in a final example, there may be a virtual ward intervention (domain 3) introduced for patients in a local area that fails to adopt any approach to addressing inequalities (a-d), and so whilst the intervention may lead to a reduction in avoidable hospital admissions per se, it may maintain or increase the disparity in avoidable admissions between socioeconomic groups within this locality. The application of the framework to these three hypothetical example interventions would therefore be summarised as follows; social prescribing intervention (2c,4c), water fluoridation (1d), virtual hospital ward intervention (3x), where x represents no approach to addressing inequalities has been adopted for the intervention.

METHODS AND ANALYSIS

The review will be carried out following established criteria for the good conduct and reporting of equity-focused systematic reviews using PRISMA-E guidelines[29-31] and reporting here conforms to the standards of the Preferred Reporting Items for Systematic Reviews and Meta-analysis Protocols (PRISMA-P, see supplementary file 1).

Preliminary searchers were carried out in September and October 2019 and the study registered with the PROSPERO International Prospective Register of Systematic Reviews[49] (registration number CRD42019153666) on 21 October 2019. The review is planned to commence March 2020 and is anticipated to take one year to complete.

Patient and Public Involvement

An advisory panel comprising collaborators from the English public health and healthcare system, national and international research communities, social and voluntary sector, and patient and public representatives will guide the research. The early engagement of patients and members of the public through the NIHR North East Research Design Service consumer panel at the stage of requesting NIHR funding to carry out this research has been instrumental in shaping this systematic review protocol. PPI members helped to strengthen the articulation of the study rationale. We received a powerful mandate from panel members to undertake the research based on their unanimous belief about its critical importance to the healthcare system and wider society. It became apparent during our discussions that the legal commitment to reducing inequality in service provision and outcome in England was not widespread public knowledge, and panel members suggested this was given more prominence in the background rationale.

Through the collaborator advisory panel, patients and the public will contribute, alongside academics, policy and practice partners, to the iterative further development of the framework (figure 1). In order to ensure meaningful participation of PPI members as the systematic review develops we will meet regularly highlighting areas for input. Outputs from this systematic review,

For peer review only - http://bmjopen.bmj.com/site/about/guidelines.xhtml

including a workshop event will be developed in close collaboration with both PPI and policy and practice partners.

Systematic review questions

- What interventions (population health, community-based, service-based and integrative) reduce socioeconomic inequalities in avoidable hospital admissions?
- What interventions (population health, community-based, service-based and integrative) maintain or increase socioeconomic inequalities in avoidable hospital admissions?

Objectives

This study has two objectives:

1. To systematically review the effectiveness of interventions (population health, community-based, service-based and integrative) in reducing socioeconomic inequalities in avoidable hospital admissions.
2. For interventions identified through the systematic review, establish where possible, how these specific interventions are organised, implemented and delivered.

Table 1 – Study inclusion and exclusion criteria

	Inclusion	Exclusion
Participants (P)	All persons of any age<br>OECD countries	Non-OECD countries
Intervention (I)	Any intervention (programme, policy, strategy, initiative, scheme or activity) across the range of domains of action (population health and policy-level, community-based, service-based and integrative)	
Comparison (C)	No intervention; usual care or practice; other interventions	
Outcomes (O)	For universal interventions;<br>• Numbers, rates, ratios, standardise mean differences, or a gradient measure<br>• Of hospitalisation overall or condition-specific hospitaladmission/readmission for any ambulatory care sensitive conditions<br>• Across socioeconomic groupings<br>For interventions targeted exclusively at socioeconomically disadvantaged populations;<br>• hospitalisation overall or condition specific hospital admission/readmission for any ambulatory care sensitive condition.	Studies that do not include a sub-analysis of effectiveness by socioeconomic group or a measure of inequality such as an absolute gradient of inequality (N/A to interventions targeted exclusively at socioeconomically disadvantaged populations)

For peer review only - http://bmjopen.bmj.com/site/about/guidelines.xhtml

Study type	• Randomised controlled trials<br>• Non-randomised or uncontrolled trials<br>• Prospective and retrospective cohort studies (with and/or without control groups)<br>• Time-series data studies<br>• Panel data studies (with and/or with control groups)<br>• Case-control studies<br>• Other ecological studies	• Descriptive studies reporting solely on avoidable admission prevalence (by person, place and time)<br>• Qualitative studies<br>• Editorial<br>• Commentary<br>• Expert opinion
Study period	Published in the last 20 years (2000-2020)	Literature published before 2000
Study reporting language	Any language	

Eligibility criteria

Studies will be selected according to the criteria outlined in table 1.

Interventions

The review will examine interventions across the range of domains of action (population health and policy-level, community-based, service-based and integrative) which might reduce socioeconomic inequalities in avoidable hospital admissions in individuals of any age. It will also uncover those interventions which may serve to maintain or increase inequalities.

The review will utilise the intervention framework and group interventions by their domain of action and by their approach to tackling inequalities (figure 1). Where possible the interventions will be grouped according to these criteria with the acknowledgement that some interventions might be cross-cutting.

Outcomes

'Avoidable admissions' are those for which timely and effective ambulatory care can prevent the need for hospitalisation[50]. Different studies use different lists of ambulatory care sensitive conditions, reflecting different types of illness (for example, whether illness is considered chronic or acute), different types of care (for example, primary care, secondary care, community care), and differences in clinical coding definitions[3 50-53]. Avoidable admissions are sometimes referred to as preventable admissions. Our definition of outcome is general and permissive of all this definitional variation within and between countries. For targeted interventions focused exclusively on socioeconomically disadvantaged populations, studies reporting a global measure of hospitalisation and those reporting condition-specific[54] hospital admission measures for any ambulatory care sensitive condition will be included. For interventions not specifically targeted at socioeconomically disadvantaged populations, studies will be included only if the differential effect of the intervention by socioeconomic status (education, income, occupation, social class, deprivation, poverty, or an area-based proxy for deprivation derived from place of residence) with respect to hospitalisations is reported. For example, using a comparison of numbers, rates, ratios, standardise mean differences, or a gradient measure of hospitalisation overall or condition-specific hospital admission/readmission for any chronic ambulatory care sensitive condition across socioeconomic groupings.

For peer review only

For peer review only - http://bmjopen.bmj.com/site/about/guidelines.xhtml

Study exclusion

The search will be limited to studies published in the last 20 years (since 2000) that have been carried out in an OECD country to ensure contemporaneity and a degree of commonality in health system maturity and socioeconomic and demographic context. We recognise that OECD countries will have different characteristics including different health systems and socio-economic situations and we will interpret the interventions that we find taking into account this variation in context.

Study design

A rigorous and inclusive international literature search for experimental, quasi-experimental and causal inference based studies will be conducted. This will include randomised and non-randomised controlled trials, non-controlled trials, prospective and retrospective cohort studies (with and/or without control groups), time series data studies, panel studies (with and/or with control groups), case-control studies, and other ecological studies (for example studies utilising administrative data) of the effectiveness of interventions at reducing inequalities in avoidable admissions to hospital. In the case of non-RCT studies the authors will make a judgement as to whether any association reported may be considered causal, taking into account the type of study undertaken and following a thorough assessment of study quality and risk of bias (see risk of bias section below).

Information sources and search strategy

Published studies in any language meeting the inclusion criteria will be sought by searching peer-reviewed literature in electronic databases including (host sites given in parentheses): MEDLINE (Ovid), Embase (Ovid), CINAHL (EBSCO), Cochrane CENTRAL (Wiley), Science Citation Index (Web of Knowledge), Social Science Citation Index (Web of Knowledge), Conference Proceedings Citation Index – Science (Web of Knowledge), Conference Proceedings Citation Index – Social Science (Web of Knowledge). All searches will be re-run prior to final analyses and any further studies identified retrieved for inclusion. Please see supplementary file 2 for the preliminary Medline search strategy.

Web of Knowledge Citation Indices will be used to undertake full citation searches of included studies. Electronic database searches will be supplemented with website searches, included but not restricted to the Kings Fund and the Nuffield Trust. We will request relevant information on unpublished and in-progress research from key experts in the field, identified through the collaborator network for this systematic review.

Study selection

All articles identified through the search will be uploaded into Eppi-reviewer 4 software[55]. Duplicates will be removed automatically and manually. Three reviewers (SS, BN and JW) will screen titles and abstracts (if available) of the retrieved articles to assess eligibility for inclusion. The initial 10% of papers will be double screened independently, and then reviewed to ensure a consistent approach to applying the inclusion criteria is being adopted. After the initial selection based on title and abstract screen, full texts of potentially eligible articles will be obtained and screened independently by two reviewers to assess eligibility for final inclusion. Disagreements at any stage will be resolved through consultation with a fourth reviewer (FB).

Data extraction

Data for included studies will be extracted by two reviewers, using a bespoke form within Eppi-reviewer 4 software[55]. The data extraction of one reviewer will be checked by the other and visa-versa. Any discrepancies will be resolved through discussion between the reviewers and, if consensus is not reached, with the wider project team (FB, RT, RC, ML, CB). The data extraction

For peer review only - http://bmjopen.bmj.com/site/about/guidelines.xhtml

form will be piloted using the first five eligible articles and modified if required (see supplementary file 3). The data extracted from each article will include, but not restricted to, author, publication year, funding source, study design, study setting including characteristics of the health system of the country, study period, number of included participants/areas, study population characteristics (including for example age and ethnicity), intervention domain (1-4), intervention approach (a-d), intervention description, definition and measurement of socioeconomic status, outcome measure(s), covariates used for adjustment (certain study types), reported limitations, key conclusions. It is anticipated studies will characterise socioeconomic groupings differently, and measure equity in a variety of ways. Therefore, the data collection tool will be designed to ensure all ways of measuring socioeconomic status and differential outcomes across groups will be recorded. If relevant information cannot be retrieved from the published articles, where possible, the manuscript authors will be contacted to request additional data.

For included studies, data on the organisation, implementation and delivery of these interventions will be sought from the manuscript and any associated papers through a linked citation search. In conjunction with an OECD healthcare system typology[56], we will use data collected from the OECD Health System Characteristic survey[57] to explore potential differences between countries and we will classify them based on the most-common health coverage that is implemented e.g. autonomic coverage, compulsory coverage, voluntary coverage or no coverage. The TIDieR[58] or TIDieR-PHP[48] checklist will be used to extract relevant contextual information. Any associated process evaluations (quantitative and qualitative) exploring how and why such interventions work will be reviewed. Examples of the implementation components that will be examined include theoretical underpinning, implementation context, experience level of the intervention team (planners and implementers), consultation and/or collaboration processes (planning and delivery stages) and resources (for example time, money, staff and equipment). The systematic review evidence combined with the implementation analysis will provide insights into what works to reduce socioeconomic inequalities in avoidable admissions to hospital, why and how.

Risk of bias assessment

The methodological quality of the included studies will be appraised independently by two reviewers using the Effective Public Health Practice Project (EPHPP) tool[59], chosen because the same tool can be used across a range of quantitative study designs. Any disagreements in reviewer quality assessment judgements will be resolved through discussion between the reviewers, and if necessary with a fourth reviewer (FB). Using the EPHPP tool, the two reviewers will make their own scientific value judgements about whether quasi-experimental methodology studies are sufficiently well designed to draw robust causal inferences, referring controversial cases to the wider review team". In rare cases, for example, robust causal inferences can be derived from uncontrolled before-after studies (and indeed from cross sectional correlations). The quality appraisal criteria will be used for descriptive purposes, to highlight variations between studies and to contribute to the assessment of overall strength of evidence in this topic area.

Analyses and synthesis

Data will be presented narratively as well as in tables (where possible) to describe the study designs, population and findings and to address each of the research questions. The main analysis will examine the effects of interventions across the domains of action on socioeconomic inequalities in avoidable hospital admission, categorising the approach adopted to address inequalities for each intervention, using the multi-dimensional framework outlined in Figure 1. Absolute and relative differences in outcome between socioeconomic groups, at baseline and follow-up, or across place and time (depending on study design), will be considered. Data will be summarised statistically

For peer review only - http://bmjopen.bmj.com/site/about/guidelines.xhtml

using meta-analysis of effect measures across socioeconomic sub-groups, where appropriate, and if studies are deemed sufficiently homogeneous to combine. Similarly, for studies exclusively focussed (in terms of study setting) on socioeconomically disadvantaged population groups, the feasibility of conducting meta-analysis for studies within this category will be assessed. Where there are sufficient numbers of studies, funnel plots and, where applicable, appropriate statistical tests will be used to assess publication bias and small study effects. Eppi Reviewer 4[55] will be used to assist in the analysis.

Where applicable and feasible, we will synthesise results for different population sub-groups separately (for example in regards to children compared to the elderly, acute compared to chronic ambulatory care sensitive conditions) where the differential impact of interventions across socioeconomic groups on hospitalisations was considered[54]. Where data permit, we will conduct further demographic subgroup analysis of the socioeconomic patterning of effect by age, gender and ethnicity. We will report our analyses in accordance with the PRISMA-E guidelines[29-31].

DISCUSSION

The review will consider interventions which reduce socioeconomic inequalities in avoidable admission to hospital. The review will also serve as a mapping exercise of the types of interventions that have been evaluated in relation to tackling inequalities in avoidable admissions, thereby highlighting any gaps in the evidence base.

For included studies, further scrutiny of any published information about them, will seek to uncover how these interventions are organised, implemented and delivered. Context is increasingly recognised as important; real world interventions are rarely implemented in isolation, and the complex interaction of interventions with the particular social context in which they are embedded determines outcomes[12 26]. However, the assessment of context and implementation has not featured strongly in previous reviews[13]. It is essential for policy and practice partners to learn not only that an intervention works to reduce inequalities in avoidable hospital per se, but why and how it managed to do this. This will enable local decision makers to compare the contextual situation to their own to make an assessment of both feasibility and likelihood of success of adopting a similar intervention in their specific socio-political, economic, cultural landscape. The ability of the review to provide this information to assist in implementation will be constrained by the extent to which this contextual information is examined and reported on in the primary studies. For example, an intervention to address socioeconomic inequalities may work for a specific ethnic group (or other sub-population) but will not work in another, and if a sub-analysis by ethnicity of the differential effectiveness of the intervention across socioeconomic groups is not carried out, or an insufficient description of the targeted population who were exposed to the intervention is not provided, this nuance will be lost.

The study design inclusion criteria in the review are broad, given that whilst trials of service-based (domain 3), and even community-based (domain 2) interventions may be likely, we expect a lack of experimental studies in relation to population health and policy-level (domain 1) and many integrative interventions (domain 4) given these tend not to be easily or routinely evaluated using experimental study designs[41 60].

Only a minority of intervention studies published report any outcome by a social determinant of health and locating these studies has previously been hampered by the absence of validated equity search filters[61]. Reflecting this, the PRISMA-E guidance recommended that systematic reviewers avoid filtering searches on equity terms due to poor indexing which would lead to relevant studies

For peer review only - http://bmjopen.bmj.com/site/about/guidelines.xhtml

being missed and advised hand-searching on these terms instead[30]. However, in 2018 an equity-focused search strategy was built and assessed for sensitivity against a gold-standard set of equity focused studies[61]. Our review adopts this newly developed and validated comprehensive search strategy for identifying studies focused on equity issues and is therefore at the forefront of developments in equity-based systematic reviewing practice. Nevertheless, a limitation remains that by not conducting full-text hand-searching on equity related terms the review may miss studies which have not made any reference to this aspect of their analysis in either the title or abstract.

Our extensive search strategy, combined with the inclusive study design criteria, will ensure that a sizeable literature will be located for synthesis. A 2012 series of reviews of interventions to reduce unplanned hospital admissions found 1530 controlled trials[13]. Whilst we recognise that the literature on the effects of interventions on socioeconomic inequalities in avoidable hospital admissions is likely to be smaller, we will maximise the likelihood of locating relevant studies by taking a more inclusive approach to study design and evaluate interventions targeted at socioeconomically disadvantaged groups or areas as well as studies that include comparative data on the differential effects of an intervention across socioeconomic groups. The size of the available evidence base will also be extended, because we will adopt a place-based, whole systems perspective, considering several domains of action (population health and policy-level, community-based, service-based and integrative).

ETHICS AND DISSEMINATION

Ethical approval is not required for this protocol or for the systematic review because it does not involve primary data collection.

We anticipate the findings for this review will contribute to an improved understanding of interventions which reduce socioeconomic inequalities in avoidable hospital admissions. Once the evidence has been synthesised a workshop will be held with invited health and social care commissioners and providers, the public health practice community who hold responsibilities for reducing health and healthcare inequalities; relevant social, voluntary and charitable sector organisations; patient and public representatives as well as national and international research community representatives to discuss the results, further refine the framework for action, aid the write-up of the study findings and facilitate the translation of the findings into practice. The research will be disseminated via national and international academic and practitioner cross-over conferences, and a paper submitted to a leading journal in this field. Furthermore, a policy-orientated summary paper will be published on an open access basis so that it is freely available to practitioners and the public.

AUTHOR CONTRIBUTIONS

SS, CB, RC, RT and ML conceived this systematic review. SS, CB, RC, RT, ML, BN, JW and FB were involved in the planning and design of the study. SS registered the protocol in PROSPERO. BN and SS with support from FB undertook preliminary literature searches in Medline. SS drafted and revised the manuscript. SS, CB, RC, RT, ML, BN, JW and FB have read, reviewed and contributed to revising and approving the final manuscript. SS obtained the research funding. SS is the guarantor.

FUNDING STATEMENT

This work was supported by the National Institute for Health Research (NIHR) and Health Education England (HEE) Integrated Clinical Academic Lecturer Fellowship (ref CA-CL-2018-04-ST2-010) and Research Capability Funding, NHS North East Commissioning Support Service. The systematic review

For peer review only - http://bmjopen.bmj.com/site/about/guidelines.xhtml

is part of **UNFAIR**: **UN**derstanding **F**actors that explain **A**voidable hospital admission **I**nequalities - **R**esearch study (http://bit.ly/UNFAIRstudy)

The funders had no role in developing the systematic review protocol. This publication presents independent research funded by the National Institute for Health Research (NIHR) and Health Education England (HEE). The views expressed are those of the author(s) and not necessarily those of the NHS, the NIHR or the Department of Health and Social Care.

COMPETING INTERESTS

None declared.

Figure 1 – Framework for addressing socio-economic (SE) inequalities in avoidable hospital admissions

Supplementary file 1 – PRISMA-P 2015 checklist

Supplementary file 2 – preliminary MEDLINE search strategy

Supplementary file 3 – data collection form

For peer review only - http://bmjopen.bmj.com/site/about/guidelines.xhtml

REFERENCES

1. Marmot M. Fair Society, Healthy Lives: The Marmot Review. London: Marmot Review; 2010
2. Cookson R, Propper C, Asaria M, et al. Socio-Economic Inequalities in Health Care in England. *Fiscal Studies* 2016;37(3-4):371-403. doi: 10.1111/j.1475-5890.2016.12109
3. OECD/EU (2018), Health at a Glance: Europe 2018: State ofHealth in the EU Cycle, OECD Publishing, Paris. Available from: https://doi.org/10.1787/health_glance_eur-2018-en (accessed 30 Oct 2019)
4. Purdy S, Huntley A. Predicting and preventing avoidable hospital admissions: A review. *Journal of the Royal College of Physicians of Edinburgh* 2013;43(4):340-44. doi: 10.4997/JRCPE.2013.415
5. Cookson R, Asaria M, Ali S, et al. Health equity monitoring for healthcare quality assurance. *Social Science & Medicine* 2018;198:148-56. doi: https://doi.org/10.1016/j.socscimed.2018.01.004
6. Asaria M, Doran T, Cookson R. The costs of inequality: whole-population modelling study of lifetime inpatient hospital costs in the English National Health Service by level of neighbourhood deprivation. *Journal of Epidemiology and Community Health* 2016;70(10):990-96. doi: 10.1136/jech-2016-207447
7. Roland M, Abel G. Reducing emergency admissions: are we on the right track? *BMJ : British Medical Journal* 2012;345 doi: 10.1136/bmj.e6017
8. Dalla Zuanna T, Spadea T, Milana M, et al. Avoidable hospitalization among migrants and ethnic minority groups: a systematic review. *European Journal of Public Health* 2017;27(5):861-68. doi: 10.1093/eurpub/ckx113
9. NHS England. CCG Improvement and Assessment Framework (CCG IAF) Data Extract 2016/17 & 2017/18 2018 Available from: https://www.england.nhs.uk/publication/ccg-iaf-data-extract/(accessed 30 Oct 2019)
10. Sheringham J, Asaria M, Barratt H, et al. Are some areas more equal than others? Socioeconomic inequality in potentially avoidable emergency hospital admissions within English local authority areas. *Journal of Health Services Research & Policy* 2017;22(2):83-90. doi: 10.1177/1355819616679198
11. Elias MJ, Le Grand. Health care and cost containment in the European Union: Routledge 2019.
12. Purdy S. Avoiding hopsital admissions: What does the research evidence say? London: Kings Fund; 2010.
13. Purdy S, Paranjothy, S., Huntley, A., Thomas, R., Mann, M., Huws, D., Brindle, P., Elwyn, G. Interventions to reduce unplanned hopsital admissions: a series of systematic reviews. NIHR Research for Patient Benefit no. PB-PG-1208-18013: University of Brisol, NHS Bristol, Cardiff University, 2012.
14. NHS England. Five year forward view. London: NHS England; 2014.
15. NHS England. New care models: Vanguards - developing a blueprint for the future of NHS and care services, 2016.
16. Baker A, Bently C, Carr D, Connolly AM, Heasman M, Johnson C, Laurent C, Tran A. Reducing health inequalities: system, scale and sustainability. London: Public Health England; 2017 PHE publications gateway number: 2017226, Crown copyright. Open Government Licence v3.0;2017.
17. WHO. Ljubljana Statement on Health Equity: Accelerating Progress Towards Health and Prosperous Lives for All in the WHO European Region. WHO Regional Office for Europe High-level conference, Ljubljana, 2019.
18. WHO. Health 2020: A European policy framework and strategy for the 21st century. Copenhagen: World Health Organisation, Regional Office for Europe, 2013.
19. Spain the Royal Decree Law 7/2018. Real Decreto-ley 7/2018, de 27 de julio, sobre el acceso universal al Sistema Nacional de Salud. Published in Boletín Oficial del Estado (Número: 183, 30/07/2018, Disposición nº 10752, Páginas: 76258-76264), 2018
20. The General Health Act (1986). Published in Boletín Oficial del Estado. Ley 14/1986, de 25 de abril, general de sanida, 1986.

For peer review only - http://bmjopen.bmj.com/site/about/guidelines.xhtml

21. National Health System Cohesion and Quality Act (2003), Published in Boletín Oficial del Estado. Ley 16/2003, de 28 de mayo, de cohesión y calidad del Sistema Nacional de Salud, 2003.

22. Great Britain. Health and Social Care Act. London: The Stationary Office; 2012.

23. NHS England. The NHS Long Term Plan. London: NHS England; 2019.

24. White MA, Jean; Heywood, Peter. How and why do interventions that increase health overall widen inequalities within populations? In: Babones SJ, ed. Social inequality and public health. Bristol: Policy Press 2009:65-82.

25. Lorenc T, Petticrew M, Welch V, et al. What types of interventions generate inequalities? Evidence from systematic reviews. *Journal of Epidemiology and Community Health* 2013;67(2):190-93. doi: 10.1136/jech-2012-201257

26. Greenhalgh T, Humphrey C, Hughes J, et al. How Do You Modernize a Health Service? A Realist Evaluation of Whole-Scale Transformation in London. *Milbank Quarterly* 2009;87(2):391-416. doi: 10.1111/j.1468-0009.2009.00562.x

27. Varcoe C, Bungay V, Browne AJ, et al. EQUIP Emergency: study protocol for an organizational intervention to promote equity in health care. *BMC Health Serv Res* 2019;19(1):687. doi: 10.1186/s12913-019-4494-2

28. Cookson R AM, Ali S, Ferguson B, Fleetcroft R, Goddard M, et al. . Health Equity Indicators for the English NHS: a longitudinal whole-population study at the small-area level. *Health Serv Deliv Res* 2016;4(26)

29. Tugwell P, Petticrew M, Kristjansson E, et al. Assessing equity in systematic reviews: realising the recommendations of the Commission on Social Determinants of Health. *BMJ* 2010;341 doi: 10.1136/bmj.c4739

30. Welch V, Petticrew M, Petkovic J, et al. Extending the PRISMA statement to equity-focused systematic reviews (PRISMA-E 2012): explanation and elaboration. *Journal of Clinical Epidemiology*;70:68-89. doi: 10.1016/j.jclinepi.2015.09.001

31. Welch V, Petticrew M, Tugwell P, et al. PRISMA-Equity 2012 Extension: Reporting Guidelines for Systematic Reviews with a Focus on Health Equity. *PLOS Medicine* 2012;9(10):e1001333. doi: 10.1371/journal.pmed.1001333

32. Ray P, Trisha G, Gill H, et al. Realist review - a new method of systematic review designed for complex policy interventions. *Journal of Health Services Research & Policy* 2005;10(1_suppl):21-34. doi:10.1258/1355819054308530

33. Anderson LM, Petticrew M, Rehfuess E, et al. Using logic models to capture complexity in systematic reviews. *Research Synthesis Methods* 2011;2(1):33-42. doi: 10.1002/jrsm.32

34. Kneale D, Thomas J, Harris K. Developing and Optimising the Use of Logic Models in Systematic Reviews: Exploring Practice and Good Practice in the Use of Programme Theory in Reviews. *PLOS ONE* 2015;10(11):e0142187. doi: 10.1371/journal.pone.0142187

35. Adam T. Advancing the application of systems thinking in health. *Health Research Policy and Systems* 2014;12(1):50. doi: 10.1186/1478-4505-12-50

36. Rutter H, Savona N, Glonti K, et al. The need for a complex systems model of evidence for public health. *The Lancet*;390(10112):2602-04. doi: 10.1016/S0140-6736(17)31267-9

37. Raine R FR, Barratt H, et al. . Challenges, solutions and future directions in the evaluation of service innovations in health care and public health. *Health Services and Delivery Research* 2016;4(16) doi: 10.3310/hsdr04160

38. Kilbourne AM, Switzer G, Hyman K, et al. Advancing Health Disparities Research Within the Health Care System: A Conceptual Framework. *American Journal of Public Health* 2006;96(12):2113-21. doi: 10.2105/AJPH.2005.077628

39. Baker A. BC, Carr D., Connolly AM., Heasman M., Johnson C., Laurent C., Tran A. Reducing health inequalities: system, scale and sustainability. London: Public Health England: PHE publications gateway number: 2017226, Crown copyright. Contains information licenced under the Open Government Licence v3.0, 2017.

For peer review only - http://bmjopen.bmj.com/site/about/guidelines.xhtml

40. Benach J, Malmusi D, Yasui Y, et al. A new typology of policies to tackle health inequalities and scenarios of impact based on Rose's population approach. *Journal of Epidemiology and Community Health* 2013;67(3):286-91. doi: 10.1136/jech-2011-200363
41. Bambra CL, Hillier FC, Moore HJ, et al. Tackling inequalities in obesity: a protocol for a systematic review of the effectiveness of public health interventions at reducing socioeconomic inequalities in obesity among adults. *Systematic Reviews* 2013;2(1):27. doi: 10.1186/2046-4053-2-27
42. Whitehead M, Dahlgren G. What can be done about inequalities in health? *The Lancet* 1991;338(8774):1059-63. doi: https://doi.org/10.1016/0140-6736(91)91911-D
43. Asaria M, Ali S, Doran T, et al. How a universal health system reduces inequalities: lessons from England. *Journal of Epidemiology and Community Health* 2016;70(7):637-43. doi: 10.1136/jech-2015-206742
44. Wenzl M, McCuskee S, Mossialos E. Commissioning for equity in the NHS: rhetoric and practice. *British Medical Bulletin* 2015;115(1):5-17. doi: 10.1093/bmb/ldv031
45. Dahlgren G, Whitehead, M. Policies and Strategies to Promote Social Equity in Health. Stockholm: Institute for Futures Studies 1991.
46. Lorna A, Mark L, Vikki E, et al. A Systematic Review of the Effectiveness of Health Service Interventions Aimed at Reducing Inequalities in Health. *Journal of Health Services Research & Policy* 1996;1(2):93-103. doi: 10.1177/135581969600100207
47. Geraint L, Sheringham J, Lopez Bernal J, et al. Mastering public health: a postgraduate guide to examinations and revalidation. Second ed. Great Britain: Boca Raton : CRC Press, Taylor & Francis Group 2015.
48. Campbell M, Katikireddi SV, Hoffmann T, et al. TIDieR-PHP: a reporting guideline for population health and policy interventions. *BMJ* 2018;361:k1079. doi: 10.1136/bmj.k1079
49. Sowden S, Nezafat Maldonado B, Beyer F, Cookson R, Thomson R, Lambert M, Bambra C. Reducing inequalities in avoidable hospital admissions: a systematic review. PROSPERO 2019 CRD42019153666 Available from: https://www.crd.york.ac.uk/prospero/display_record.php?ID=CRD42019153666 (accessed 30 Oct 2019)
50. Billings JW, Weinick RM Monitoring the Health Care Safety Net Book II: A Data Book for States and Counties 2003 https://permanent.access.gpo.gov/lps48692/databk2.pdf (accessed 26 Feb 2020)
51. Health and Social Care Information Centre, CCG Outcome Indicator 3.1: Emergency admissions for acute conditions that should not usually require hospital admission - technical specification, 2018, England.
52. Health and Social Care Information Centre, CCG Outcome Indicator 2.6: Unplanned hopsitalisation for chronic ambulatory care senstive conditions, 2018, England.
53. Agency for Healthcare Research and Quality, Guide to Prevention Quality Indicators: Hospital Admission for Ambulatory Care Sensitive Conditions USA: Department of Health and Human Services, Agency for Healthcare Resaearch and Quality; 2001 https://www.ahrq.gov/downloads/pub/ahrqqi/pqiguide.pdf (accessed 26 Feb 2020)
54. NHS England CCG Improvement and Assessment Framework (CCG IAF) 2017/18: Technical annex (version 1.1). London: NHS England; 2017.
55. EPPI-Centre, Social Science Research Unit, UCL Institute of Education, University of London, UK. Eppi-Reviewer 4 systematic review software, Available from: https://eppi.ioe.ac.uk/CMS/Default.aspx?alias=eppi.ioe.ac.uk/cms/er4& (accessed 30 Oct 2019)
56. Reibling N, Ariaans M, Wendt C. Worlds of Healthcare: A Healthcare System Typology of OECD Countries. *Health Policy* 2019;123(7):611-20. doi: https://doi.org/10.1016/j.healthpol.2019.05.001
57. OECD, OECD Health System Characteristics survey, 2016, https://qdd.oecd.org/subject.aspx?Subject=hsc (accessed 13 Feb 2020)
58. Hoffmann TC, Glasziou PP, Boutron I, et al. Better reporting of interventions: template for intervention description and replication (TIDieR) checklist and guide. *BMJ : British Medical Journal* 2014;348:g1687. doi: 10.1136/bmj.g1687

For peer review only - http://bmjopen.bmj.com/site/about/guidelines.xhtml

59. Thomas BH, Ciliska D, Dobbins M, Micucci S. Effective Public Health Practice Proect: Quality Assessment tool for quantitative studies: McMaster University school of nursing https//:merst.ca/ephpp (accessed 11 Oct 2019).
60. Ogilvie D, Adams J, Bauman A, et al. Using natural experimental studies to guide public health action: turning the evidence-based medicine paradigm on its head. *Journal of Epidemiology and Community Health* 2019:jech-2019-213085. doi: 10.1136/jech-2019-213085
61. Prady SL, Uphoff EP, Power M, et al. Development and validation of a search filter to identify equity-focused studies: reducing the number needed to screen. *BMC Medical Research Methodology* 2018;18(1):106. doi: 10.1186/s12874-018-0567-x

For peer review only

For peer review only - http://bmjopen.bmj.com/site/about/guidelines.xhtml

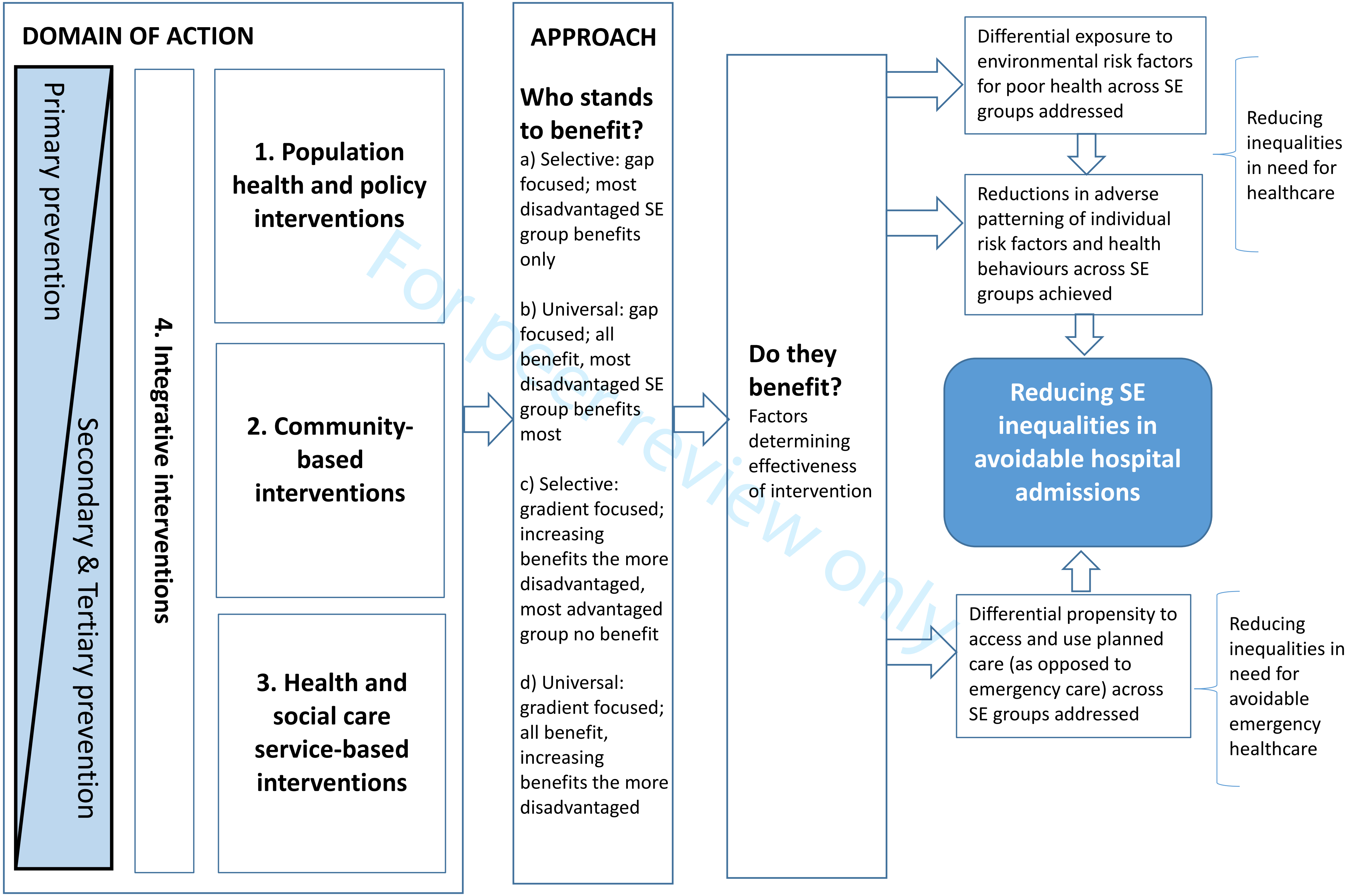

For peer review only

Reporting checklist for protocol of a systematic review.

Based on the PRISMA-P guidelines.

Moher D, Shamseer L, Clarke M, Ghersi D, Liberati A, Petticrew M, Shekelle P, Stewart LA. Preferred Reporting Items for Systematic Review and Meta-Analysis Protocols (PRISMA-P) 2015 statement. Syst Rev. 2015;4(1):1.

		Reporting Item	Page Number
**Title**			
Identification	#1a	Identify the report as a protocol of a systematic review	1
Update	#1b	If the protocol is for an update of a previous systematic review, identify as such	N/A
**Registration**			
	#2	If registered, provide the name of the registry (such as PROSPERO) and registration number	2
**Authors**			
Contact	#3a	Provide name, institutional affiliation, e-mail address of all protocol authors; provide physical mailing address of corresponding author	1
Contribution	#3b	Describe contributions of protocol authors and identify the guarantor of the review	12
**Amendments**			
	#4	If the protocol represents an amendment of a previously completed or published protocol, identify as such and list changes; otherwise, state plan for documenting important protocol amendments	N/A
**Support**			
Sources	#5a	Indicate sources of financial or other support for the review	12

For peer review only

Sponsor	#5b	Provide name for the review funder and / or sponsor	12
Role of sponsor or funder	#5c	Describe roles of funder(s), sponsor(s), and / or institution(s), if any, in developing the protocol	12
**Introduction**			
Rationale	#6	Describe the rationale for the review in the context of what is already known	3-6
Objectives	#7	Provide an explicit statement of the question(s) the review will address with reference to participants, interventions, comparators, and outcomes (PICO)	7
**Methods**			
Eligibility criteria	#8	Specify the study characteristics (such as PICO, study design, setting, time frame) and report characteristics (such as years considered, language, publication status) to be used as criteria for eligibility for the review	7-8
Information sources	#9	Describe all intended information sources (such as electronic databases, contact with study authors, trial registers or other grey literature sources) with planned dates of coverage	9
Search strategy	#10	Present draft of search strategy to be used for at least one electronic database, including planned limits, such that it could be repeated	Suppl file 2
Study records - data management	#11a	Describe the mechanism(s) that will be used to manage records and data throughout the review	10
Study records - selection process	#11b	State the process that will be used for selecting studies (such as two independent reviewers) through each phase of the review (that is, screening, eligibility and inclusion in meta-analysis)	9
Study records - data collection process	#11c	Describe planned method of extracting data from reports (such as piloting forms, done independently, in duplicate), any processes for obtaining and confirming data from investigators	9-10

For peer review only

Data items	#12	List and define all variables for which data will be sought (such as PICO items, funding sources), any pre-planned data assumptions and simplifications	7-8
Outcomes and prioritization	#13	List and define all outcomes for which data will be sought, including prioritization of main and additional outcomes, with rationale	7-8
Risk of bias in individual studies	#14	Describe anticipated methods for assessing risk of bias of individual studies, including whether this will be done at the outcome or study level, or both; state how this information will be used in data synthesis	10
Data synthesis	#15a	Describe criteria under which study data will be quantitatively synthesised	10-11
Data synthesis	#15b	If data are appropriate for quantitative synthesis, describe planned summary measures, methods of handling data and methods of combining data from studies, including any planned exploration of consistency (such as I2, Kendall's τ)	10-11
Data synthesis	#15c	Describe any proposed additional analyses (such as sensitivity or subgroup analyses, meta-regression)	11
Data synthesis	#15d	If quantitative synthesis is not appropriate, describe the type of summary planned	10-11
Meta-bias(es)	#16	Specify any planned assessment of meta-bias(es) (such as publication bias across studies, selective reporting within studies)	10-11
Confidence in cumulative evidence	#17	Describe how the strength of the body of evidence will be assessed (such as GRADE)	10-11

Notes:

- 10: Suppl file 2 The PRISMA-P checklist is distributed under the terms of the Creative Commons Attribution License CC-BY 4.0. This checklist was completed on 31. October 2019 using https://www.goodreports.org/, a tool made by the EQUATOR Network in collaboration with Penelope.ai

For peer review only

Supplementary file 2 – preliminary MEDLINE search strategy

Element	Search items
Hospital admissions [AND]	Hospitals/statistics & numerical data or Emergency Service, Hospital/statistics & numerical data* or Hospitalization/ec, td [Economics, Trends] or Patient Readmission/ or Patient Admission"/ec, td [Economics, Trends] or Emergency hospital admission*.ti,ab or emergency hospitali#ation.ti,ab or unplanned hospitali#ation.ti,ab or (overnight stay adj5 admission*).ti,ab or (primary care adj5 admission*).ti,ab or (ambulatory care adj5 admission*).ti,ab.or (admission* adj5 emergenc*).ti,ab.or (Emergency Treatment adj5 admission*).ti,ab.or . ((emergency care adj5 admission*) or readmission*).mp or (emergency room adj5 admission*).ti,ab. or emergency admission*.mp.or overnight stay.mp. or emergency medical admission*.mp.or (hospital* adj5 readmission rates).mp.or ((unscheduled or unplanned or un-planned or unanticipated or unexpected) adj5 (admission* or readmission* or hospitali#ation or care)).mp or (admissions adj5 hospital days).mp.or . (hospital admission* adj5 emergenc*).mp.
Equity [AND}	Combined equity filter MEDLINE – see additional file 1[1]
Intervention [AND]	exp."Health Care Quality, Access.mp. or Health Care.mp. or exp "Delivery of Health Care"/ or Chronic Disease/therapy* or State Medicine/statistics & numerical data or programme evaluation.mp or intervention$.tw. or evaluat$.tw. or policy.tw or strategy.tw. or access to care.mp.or access to services.mp. or scheme.tw or program*.tw or initaitve.tw or ambulatory care/ or aftercare/ or after-hours care/ or case management/ or "continuity of patient care"/ or patient education/ or health services accessibility/ or "deliver of health care"/ or Home Care Services, Hospital-Based/ or Managed Care Program*/ or Health Knowledge, Attitudes, Practice/ or *"Outcome Assessment (Health Care)"/ or "Drug Utili$ation Review"/ or Intermediate Care Facvilities/ or *"Self Care"/ or Community Health Services/ or "Patient Discharge"/ or Health Services Research/ or Primary Health Care/ or Physicians, Family/ or Treatment Outcome/ or Risk Assessment/ or Telemedicine/ or Quality of care.mp. or (out-of-hours or OOH).mp. or access to care.mp or access to services.mp or continuity of care.mp or medication review.mp or organisation of care.mp or outreach.mp or community matron.mp or walk in centres.mp or telemonitoring.mp or "hospital at home".mp or "virtual wards".mp or self management.mp or assessment units.mp or observation wards.mp or GPS in A&E.mp or ddischarge plan*.or mp primary care.mp or telephone follow-up.mp or home telecare.mp or on-line health.mp (e0health or ehealth).mp or home telemedicine.mp or general practitioner*.mp or health Promotion/ or health promotion.ti,ab. or health behaviour.ti,ab. or health behavior.ti,ab. or (policy and (social or public or urban or environmental or fiscal)).ti,ab. or urban planning.ti,ab. or city planning.ti,ab. or built environment.ti,ab. or social environment.ti,ab. or physical environment.ti,ab. or cultural environment.ti,ab. or urban environment.ti,ab. or neighbourhood.ti,ab. or community.ti,ab. or societal.ti,ab. or social interventions.ti,ab. or community interventions.ti,ab. or individual level.ti,ab. or lifestyle.ti,ab. or individual.ti,ab. or tax$.ti,ab. or subsid$.ti,ab. or price$.ti,ab. or health education.ti,ab. or social marketing.ti,ab. or (diet and (advice or counselling)).ti,ab. or (exercise and (advice or counselling)).ti,ab. or cash transfer$.ti,ab. or lifestyle counselling.ti,ab. or behavioural counselling.ti,ab. or behavioral counselling.ti,ab. or workplace.ti,ab. or campaign$.ti,ab. or (access adj1 facilities).ti,ab
Study type [AND]	(evaluat$ or effective$ or Intervention or RCT or experiment$ or randomi?ed controlled trial$ or clinical randomi?ed controlled trial$ or cluster randomi?ed controlled trial$ or double blind randomi?ed controlled trial$ or randomi?ed consent design or single blind randomi?ed controlled trial$ or randomi?ed or placebo or random$ or trial or quasi?experiment$ or pre$test or post$test or trial or time series or evaluat$ or intervention$ or "before and after" or intervention$ or commun! ity trial or non?randomi?ed or repeat$ or repeat$ measures).ti,ab. or (exp Clinical Trial/ or exp Randomized Controlled Trial/ or exp Randomization/ or exp Double-Blind Method/ or exp Single-Blind Method/ or exp Cross-Over Studies/) or clinical trial.ti,ab. or latin square.ti,ab. or random$.ti,ab. or exp Evaluation/ or clinical trial.ti,ab. or clinical trial.pt. or (before adj1 after adj1 (stud$ or trial$ or design$)).ti,ab. or random$.ti,ab. or (quasi?experimental or pseudo?experimental).ti,ab. or (nonrandomi?ed or non?randomi?ed or pseudo?randomi?sed or quasi?randomi?ed).ti,ab. or ((population level or population based or population orientated or population oriented or

	community level or community based or community orientated or community oriented) adj3 (intervention$ or prevention or policy or policies or program$ or project$)).ti,ab.
OECD countries only [NOT]	(Algeria$ or Egypt$ or Liby$ or Morocc$ or Tunisia$ or Western Sahara$ or Angola$ or Benin or Botswana$ or Burkina Faso or Burundi or Cameroon or Cape Verde or Central African Republic or Chad or Comoros or Congo or Djibouti or Eritrea or Ethiopia$ or Gabon or Gambia$ or Ghana or Guinea or Keny$ or Lesotho or Liberia or Madagasca$ or Malawi or Mali or Mauritania or Mauritius or Mayotte or Mozambiq$ or Namibia$ or Niger or Nigeria$ or Reunion or Rwand$ or Saint Helena or Senegal or Seychelles or Sierra Leone or Somalia or South Africa$ or Sudan or Swaziland or Tanzania or Togo or Ugand$ or Zambia$ or Zimbabw$ or China or Chinese or Hong Kong or Macao or Mongolia$ or Taiwan$ or Belarus or Moldov$ or Russia$ or Ukraine or Afghanistan or Armenia$ or Azerbaijan or Bahrain or Cyprus or Cypriot or Georgia$ or Iran$ or Iraq$ or Israel$ or Jordan$ or Kazakhstan or Kuwait or Kyrgyzstan or Leban$ or Oman or Pakistan$ or Palestin$ or Qatar or Saudi Arabia or Syria$ or Tajikistan or Turkmenistan or United Arab Emirates or Uzbekistan or Yemen or Bangladesh$ or Bhutan or British Indian Ocean Territory or Brunei Darussalam or Cambodia$ or India$ or Indonesia$ or Lao or People's Democratic Republic or Malaysia$ or Maldives or Myanmar or Nepal or Philippin$ or Singapore or Sri Lanka or Thai$ or Timor Leste or Vietnam or Albania$ or Andorra or Bosnia$ or Herzegovina$ or Bulgaria$ or Croatia$ or Estonia or Faroe Islands or Greenland or Liechtenstein or Lithuani$ or Macedonia or Malta or maltese or Romania or Serbia$ or Montenegro or Slovenia or Svalbard or Argentina$ or Belize or Bolivia$ or Brazil$ or chile or Chilean or Colombia$ or Costa Rica$ or Cuba or Ecuador or El Salvador or French Guiana or Guatemala$ or Guyana or Haiti or Honduras or Jamaica$ or Nicaragua$ or Panama or Paraguay or Peru or Puerto Rico or Suriname or Uruguay or Venezuela or developing countr$ or south America$).ti,sh. [mp=title, abstract, original title, name of substance word, subject heading word, floating sub-heading word, keyword heading word, organism supplementary concept word, protocol supplementary concept word, rare disease supplementary concept word, unique identifier, synonyms]
Filter applied	Humans only
Filter applied	1999-2019

1. Prady SL, Uphoff EP, Power M, et al. Development and validation of a search filter to identify equity-focused studies: reducing the number needed to screen. *BMC Medical Research Methodology* 2018;18(1):106. doi: 10.1186/s12874-018-0567-x

For peer review only

Manuscript ID bmjopen-2019-035429, entitled "Reducing inequalities in avoidable hospital admissions: explanatory framework and systematic review protocol."

DATA EXTRACTION FORM

This is an example of the data extraction form that we will use in the systematic review. This process will be done in EPPI-reviewer[a].

**STUDY DETAILS**	
Author	
Publication year	
Funding source	
Country	e.g. USA, Mexico, etc.
Further location details / study setting details	e.g. city, state, region etc.
Health care system characteristics	
Most common type health coverage[b]	☐ autonomic coverage<br>☐ compulsory coverage,<br>☐ voluntary coverage<br>☐ no coverage
OECD Healthcare System typology[c]	☐ Supply and choice oriented public type<br>☐ Performance and primary-care oriented public type<br>☐ Regulation-oriented public type<br>☐ Low supply and low performance mixed type<br>☐ Supply and performance oriented private type
Study period	e.g. 3 years
Study type (tick the appropriate option)	☐ Randomised controlled trials<br>☐ Non-randomised or uncontrolled trials<br>☐ Prospective and retrospective cohort studies (with and/or without control groups)<br>☐ Prospective repeat cross-sectional (panel) studies (with and/or with control groups)<br>☐ Ecological studies<br>☐ Economic evaluation<br>☐ Other (Please specify): _____________
Description of study methodology	
Confounders<br>*Other variables controlled for/consider in the analysis*	e.g. sex, ethnicity, age etc.
Blinding<br>*Details of this was done*	☐ No<br>☐ Yes (details): ______________________________<br>☐ N/A
**POPULATION**	
Age range	e.g. adults aged 18 and over
number of included participants/areas	

For peer review only

Clustering	e.g. individuals in areas etc.
Gender	☐ Men<br>☐ Women<br>☐ Other: _______
General study population characteristics (eligibility criteria for participation)	
Targeted or universal intervention?	☐ Targeted intervention<br>☐ Universal intervention
If targeted, explanation of target group	e.g. homeless groups, those in receipt of Medicaid, etc.
If universal, description of subdivision of outcome by SES. *What measure/marker of SES used and what subdivision made.*	e.g. most deprived quintile/decile of population vs rest
Comparison group	e.g. another city, same group before and after, or no comparison etc
**INTERNVETION**	
Intervention Approach* *Please see definitions of each intervention below*	☐ Population health and policy intervention<br>☐ Community based intervention<br>☐ Health and social care service<br>☐ Integrative intervention
Description of intervention *(programme, policy, strategy, initiative, scheme or activity)*	e.g. fluoridation of drinking water, out of hospital support package, etc.
Details of provider of the intervention	e.g. national health provider, local government, private entity, etc.
Were there variations in the intervention between target groups?	☐ No<br>☐ Yes
Who provided the intervention?	e.g. nurse, doctor, social worker, etc.
How was the intervention delivered?	e.g. telephone, face to face, etc.
Intervention date/period	
Other variables e.g. time	
**OUTCOME**	

For peer review only

Outcome reported in study	☐ Hospital admission/hospitalisation<br>☐ Hospital readmission<br>☐ Emergency hospital admission<br>☐ Avoidable hospital admission<br>☐ Unspecified<br>Definition____
Unit of analysis	e.g. Individual patient, lower super output area/neighbourhood, local authority, district etc.
Global or condition specific?	☐ Global<br>☐ Specific condition: ___________________
Detailed description of outcome variable	e.g. admission for complication of diabetes in patents with existing diagnosis of diabetes.
Justification for avoidable classification	e.g. One of ICD10 codes included in list of ambulatory care sensitive conditions
**Outcome Measure:**	
For the targeted interventions…	
No. of admission in intervention group	
Rate of intervention group admissions in Intervention group	
No. of admissions in control group if applicable	
Rate of admissions in control group if applicable	
Has sub-analysis of the outcome by SES and then by e.g. ethnicity, gender etc. been reported? And figures here.	☐ Yes (other variables)______________________<br>☐ No<br>☐ Data_____________________________
For universal interventions:	
What measure of SES is used?	e.g. IMD 2015 quintile of deprivation
No. of admission across SES groups	
Rate of admissions across SES groups	
OR across SES groups	
RR	
SMD	
Pooled proportions	
Measure of gradient	e.g. absolute gradient of inequality, gini coefficient etc
Other measures	

For peer review only

Has sub-analysis of SES and then breakdown by other variables been undertaken (e.g. ethnicity, gender)	☐ Yes (over variables):_________<br>☐ No<br>☐ Data_______________________
Outcome measures in control group *if applicable*	
Who benefits the most?	☐ Targeted intervention to those in most disadvantaged socioeconomic group only, focused on closing the health gap (selective: gap focused)<br>☐ Universal intervention, with additional focus on those in the most disadvantaged socioeconomic group to close the health gap (universal: gap focused).<br>☐ Redistributive intervention, a universally applied intervention but where the extent of benefit increases across the social gradient, such that the most socioeconomically advantaged are not expected to benefit at all from the intervention due to lack of need for it (selective: gradient focused).<br>☐ Proportionate universalism, universal intervention benefitting all, but with increasing benefits of intervention across the social gradient (universal: gradient focused).
Evidence for above statement	Interpretation of e.g. ORs etc. extracted
Comments/Additional relevant info	
**IMPLEMENTION**<br>*This information will be useful for assessing generalisability of findings/validity of pooling studies to do metanalysis. Details will be sought from the manuscripts and any associated papers through a linked citation search*	
Date of implementation	
Delivery dates	
Theoretical underpinning for intervention:	
Implementation context:	
Members of intervention team	
Experience level of the intervention team (planners and implementers)	
Consultation and/or collaboration processes (planning and delivery stages)	
Resources (for example time, money, staff and equipment)	

For peer review only

The TIDieR[d] or TIDieR-PHP[e] checklists will be used to extract further relevant information<br>*See checklists below*<br>.

a) EPPI-Centre, Social Science Research Unit, UCL Institute of Education, University of London, UK. Eppi-Reviewer 4 systematic review software, Available from: https://eppi.ioe.ac.uk/CMS/Default.aspx?alias=eppi.ioe.ac.uk/cms/er4& (accessed 30 Oct 2019)
b) 57. OECD, OECD Health System Characeristics survey, 2016, https://qdd.oecd.org/subject.aspx?Subject=hsc (accessed 13 Feb 2020)
c) 56. Reibling N, Ariaans M, Wendt C. Worlds of Healthcare: A Healthcare System Typology of OECD Countries. *Health Policy* 2019;123(7):611-20. doi: https://doi.org/10.1016/j.healthpol.2019.05.001
d) 58. Hoffmann TC, Glasziou PP, Boutron I, et al. Better reporting of interventions: template for intervention description and replication (TIDieR) checklist and guide. *BMJ : British Medical Journal* 2014;348:g1687. doi: 10.1136/bmj.g1687
e) 48. Campbell M, Katikireddi SV, Hoffmann T, et al. TIDieR-PHP: a reporting guideline for population health and policy interventions. *BMJ* 2018;361:k1079. doi: 10.1136/bmj.k1079

*Intervention Approaches

<u>Population health and policy intervention</u>

Legal, fiscal, structural, organisational, environmental, and policy interventions that seek to change health related behaviours or to modify the social and economic determinants of health. This includes interventions with other goals that bring about such changes as a by product.

<u>Community-based intervention</u>
Community resources and assets, for example local area coordination initiatives.

<u>Health and social care service</u>
In this context these might include interventions considered in previous reviews of avoidable admissions (for example, disease and medication management and education programmes, vaccination, structured discharge planning, comprehensive geriatric assessment, virtual wards, hospital at home initiatives) as well as primary care (provision, access, quality, continuity), benchmarking, predictive risk-modelling and case-management interventions.

<u>Integrative intervention</u>
Interventions that create greater synergy and closer working between domains 1 – 3 or within 3 (between for example, primary and secondary healthcare and social care). For example, social prescribing initiatives facilitate closer working between domains 2 and 3.

For peer review only

Campbell M, Katikireddi SV, Hoffmann T, et al. TIDieR-PHP: a reporting guideline for population health and policy interventions. *BMJ* 2018;361:k1079.

Item	Item description	Page in manuscript where item is reported	Other*
1 Brief name	Provide the name or a phrase that describes the intervention		
2 Why	Describe the logic, mechanisms, or rationale of the intervention, clearly linking intervention elements to the expected effects on immediate or longer term outcomes (or both)		
3 What materials	Describe any materials used in the intervention (including online appendices or URLs for further details). For example:<br>—informational materials (may include those provided to recipients of the intervention or in training of intervention providers)<br>—nature and value of any benefit provided (eg, cash, voucher, meal)<br>—any physical resources or infrastructure provided as part of the intervention		
4 What and how	Describe how the intervention was planned, established, and intended to be delivered. Depending on the type of intervention, it may be useful to consider:<br>—how sources of funding for the intervention and the service providers were obtained, how users were enrolled and the		

Item	Item description	Page in manuscript where item is reported	Other*
	service delivered —how any payments were made or benefits delivered, how qualifying conditions were implemented —the entity being regulated, the scope of the regulation, permitted level of use; procedures for monitoring or enforcing compliance, and any sanctions for non-compliance —how people were exposed to the intervention, whether it was provided to individuals or larger populations —any underpinning legislation including name, date passed, and legislative body		
5 Who provided	Describe the provider of the intervention, including legal status and powers, field organisations and staff responsible for planning, implementation, monitoring and enforcement. Where relevant, describe intervention provider expertise and training (general or specific to the intervention)		
6 Where	Describe the type of location (eg, school, community centre) and the geographical scope of the intervention (eg, national, regional, city-wide). Where relevant, describe the historical, cultural, socioeconomic,		

Item	Item description	Page in manuscript where item is reported	Other*
	or political background to the intervention		
7 When and how often	Describe when the intervention was implemented, how long it remained in place, and, if applicable, the number, duration, and scheduling of occasions		
8.1 Planned variation	Describe and provide the reason for any variation or tailoring that was planned or allowed for in the design of the intervention. Examples include differences between locations, geographical areas, population subgroups, or over time		
8.2 Unplanned variation	Describe and provide the reason for any unplanned variation or modifications in the intervention (eg, between different locations, geographical areas, population subgroups, or over time) that were made after the intervention commenced		
9.1 How well	Describe any strategies used or actions taken to maintain fidelity of the intervention (ie, to ensure that the intervention was delivered as intended)		
9.2 How well—delivery	Describe the fidelity of the intervention (ie, the extent to which the intervention was delivered as intended)		

- *If the information is not provided in the primary paper, give details of where this information is available (eg, protocol, other published papers (provide citation details), or a website (provide the URL))

Hoffmann TC, Glasziou PP, Boutron I, et al. Better reporting of interventions: template for intervention description and replication (TIDieR) checklist and guide. *BMJ : British Medical Journal* 2014;348:g1687. doi: 10.1136/bmj.g1687

Item No	Item	Pg no. in manuscript where reported
**Brief name**		
1	Provide the name or a phrase that describes the intervention	
**Why**		
2	Describe any rationale, theory, or goal of the elements essential to the intervention	
**What**		
3	Materials: Describe any physical or informational materials used in the intervention, including those provided to participants or used in intervention delivery or in training of intervention providers. Provide information on where the materials can be accessed (such as online appendix, URL)	
4	Procedures: Describe each of the procedures, activities, and/or processes used in the intervention, including any enabling or support activities	
**Who provided**		
5	For each category of intervention provider (such as psychologist, nursing assistant), describe their expertise, background, and any specific training given	
**How**		
6	Describe the modes of delivery (such as face to face or by some other mechanism, such as internet or telephone) of the intervention and whether it was provided individually or in a group	
**Where**		
7	Describe the type(s) of location(s) where the intervention occurred, including any necessary infrastructure or relevant features	
**When and How Much**		
8	Describe the number of times the intervention was delivered and over what period of time including the number of	

For peer review only

Item No	Item	Pg no. in manuscript where reported
	sessions, their schedule, and their duration, intensity, or dose	
**Tailoring**		
9	If the intervention was planned to be personalised, titrated or adapted, then describe what, why, when, and how	
**Modifications**		
10	If the intervention was modified during the course of the study, describe the changes (what, why, when, and how)	
**How well**		
11	Planned: If intervention adherence or fidelity was assessed, describe how and by whom, and if any strategies were used to maintain or improve fidelity, describe them	
12	Actual: If intervention adherence or fidelity was assessed, describe the extent to which the intervention was delivered as planned	

For peer review only
